# Catalyst-Dependent Chemoselectivity in the Dirhodium-Catalyzed Cyclization Reactions Between Enodiazoacetamide and Nitrosoarene: A Theoretical Study

**DOI:** 10.3389/fchem.2019.00586

**Published:** 2019-08-23

**Authors:** Yan Zhang, Yongsheng Yang, Ruyu Zhu, Xingyu Wang, Ying Xue

**Affiliations:** Key Lab of Green Chemistry and Technology, Ministry of Education, College of Chemistry, Sichuan University, Chengdu, China

**Keywords:** DFT, mechanism, cyclization, dirhodium, chemoselectivity

## Abstract

N-O heterocycle compounds play an important role in various fields. Doyle et al. (Cheng et al., 2017) have recently reported an efficient catalyst-controlled selective cyclization reactions of *tert*-butyldimethylsilyl (TBS)-protected enoldiazoacetamides with nitrosoarenes in which the multifunctionalized products 5-isoxazolones and 1,3-oxazin-4-ones were formed through [3+2]- and [5+1]-cyclizations using Rh_2_(oct)_4_ and Rh_2_(cap)_4_ as catalysts, respectively. The present work studied the mechanism of the reactions in question and the origins of the catalyst-dependent chemoselectivity by the density functional theory (DFT) calculations at the M06-D3/SMD/6-311+G(d,p)//B3LYP-D3/6-31G(d,p) level of theory. The computed results illustrate the importance of the different dirhodium catalysts to gain diversified N-O heterocycle compounds and suggest the specific interaction between the reactants by the real catalyst model. Meanwhile, it is the steric hindrance and electronic effect of the ligands of dirhodium catalysts that control the reaction mechanism. Furthermore, the other auxiliary theoretical analysis, natural bond orbital calculation and distortion/interaction analysis, make the electronic effects, and steric hindrance more distinct.

**Graphical Abstract:**
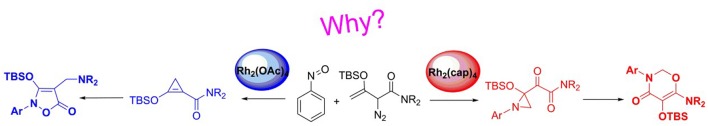
**GRAPHICAL ABSTRACT** Catalyst-dependent chemoselectivity.

## Introduction

**Scheme 1:**
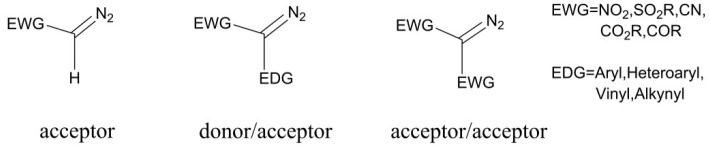
Three classes of diazo compounds.

**Scheme 2:**
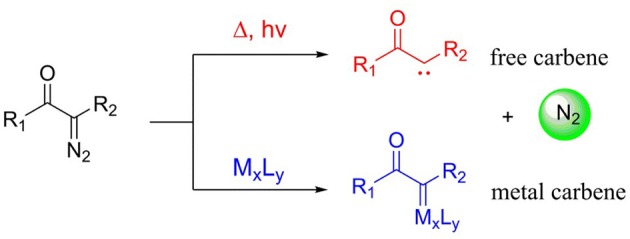
The process of diazo compound becoming to carbene.

Diazo compounds play a significant role in organic synthetic field, and these compounds are always classified as three typical classes: acceptor, donor/acceptor, and acceptor/acceptor, according to the electronic effect of the substituents (Davies and Beckwith, 2003; Zhu et al., 2008), as shown in Scheme 1. However, the thermal stability of these diazo compounds is not good, which is depended on the electronic effect of the substituents connected with the diazo carbon atom, and the acceptor/acceptor type is the most stable one (Regitz and Maas, 1986; Mass, 2009). Because of their poor thermal stability, diazocompounds can become to the active carbene with one molecule nitrogen expulsed in the presence of heat, light, or metal catalyst. And under the circumstance of the heat and light, the diazo compounds will come into being free carbene, which is limitedly applied owing to the extremely unstable character. However, the metal carbenes generated by the interaction of metal catalyst with diazo compoundsare more stable and also well-applied in organic synthesis (Doyle et al., 1998; Mass, 2009), and these two ways of carbene formation are shown in Scheme 2. As for the formation of metal carbene, this is a nucleophilic addition step between metal catalyst and diazo compound (Holzwarth et al., 2012). Due to their good stability, high activity, and significant selectivities (chemo-, regio-, and stereoselectivities), those metal carbenes have been paid more attention by chemists in recent years (Denton and Davies, 2009). Moreover, the active metal carbenes were widely used in the reactions of C-H bond functionalization, C-H insertion, alkene cyclopropanation, and ylide formation (Doyle et al., 1998; Davies and Beckwith, 2003; Li and Davies, 2009).

Due to the difference of substituents in diazo compound, the corresponding metal carbenes show different characteristics. Moreover, the diverse metal carbenes have certain pertinence to different reactions. For example, the metal carbene generated by the reaction of metal catalyst with acceptor-substituted diazo compound has a great stereoselectivity for alkene cyclopropanation reactions (Hu et al., 2002; Zhu et al., 2008), while the characters of donor/acceptor-substituted carbenes, like vinyldiazoesters, and aryldiazoesters, have been greatly improved and can be applied to more reactions (Davies, 1999; Davies and Beckwith, 2003). However, the acceptor/acceptor metal carbenes take on low activity and poor enatioselectivity for asymmetric cyclopropanation reactions (Doyle, 1986; Davies and Beckwith, 2003; Zhu et al., 2008). Therefore, the selection of substituents is so important for chemical reaction.

On the other hand, the diverse metal catalysts interacted with diazo compounds gain a number of metal carbenes, such as copper-carbene (Salomon and Kochi, 1973; Zhao et al., 2012), iron-carbene (Holzwarth et al., 2012), gold-carbene (Ye et al., 2010), rhodium-carbene (Denton and Davies, 2009; Shi et al., 2013), and other transition metal carbenes. Among the various metal carbenes, the rhodium-carbene with high catalytic activity is the most prominent. By the way, most of rhodium catalysts are dimers and have good symmetry, like the cage compound. Besides the aforementioned C-H bond functionalization, C-H insertion, and alkene cyclopropanation reactions, rhodium-carbene compounds have also significant reactivity for cycloaddition reactions, especially for the synthesis of five- and six-membered heterocyclic compounds (Pirrung and Blume, 1999; Mehta and Muthusamy, 2002; Reddy and Davies, 2007). Such as the illudins, epoxysorbicilinol, brevicomin, and other important natural products, they all can be successfully synthesized by the cycloaddition reaction of rhodium carbene compounds (Muthusamy et al., 2007). Among multitudinous dirhodium(II) carbenoids, the simplest one is the dirhodium acetate carbenoid (Rh_2_(OAc)_4_). Furthermore, by changing the ligands of the dirhodium catalysts, the electron-rich or electron-deficient dirhodium catalysts can be obtained. And then various dirhoduim(II) carbenes are generated and widely used in organic synthesis. Through the electronic and steric hindrance effects of the ligands, the corresponding dirhoduim(II) carbene owns the better selectivity characters (Vitale et al., 2006; Zhang et al., 2018). For example, Rh_2_(cap)_4_ catalyst is formed through the substituting to four acetate ligands in Rh_2_(OAc)_4_ using caprolactamate groups and then becomes Rh_2_(cap)_4_-carbenoid compound. Noticeably, the most important thing is that divergent catalysts can catalyze the same substrates to produce different products through different reactions, as reported recently by Dolye group (Cheng et al., 2017). They reported the cyclization reactions between *tert*-butyldimethylsilyl (TBS)-protected enoldiazoacetamides and nitrosoarenes catalyzed by divergent dirhodium compounds, in which when the catalyst was Rh_2_(OAc)_4_, Rh_2_(oct)_4_, Rh_2_(tpa)_4_, or Rh_2_(pfb)_4_, the final main product was observed to be the five-membered ring compound by [3+2]-cycloaddition, while there was a six-membered ring product through [5+1]-cycloaddition with the Rh_2_(cap)_4_ being the catalyst. Hence, it can be seen that the diverse dirhodium catalysts act a significant role in synthetic chemistry.

**Scheme 3:**
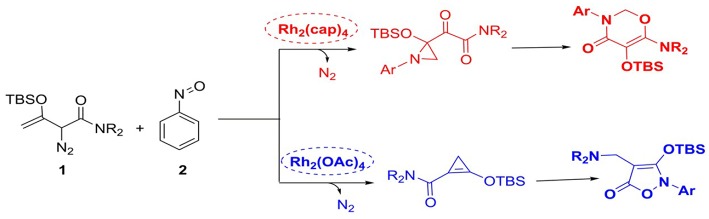
The cycloaddition reactions of enodiazoacetamide and nitrosoarene catalyzed by divergent dirhodium catalysts.

With the recent development of theoretical methods, the combination of quantum chemical calculations with the experimental researches has made the significant advances in the chemistry. Among the theoretical computational study, the density functional theory (DFT) is the most prominent method (Kohn and Sham, 1965; Dreizler and Gross, 2012; Gross and Dreizler, 2013; Koch and Holthausen, 2015). Theoretical study can explain the experimental phenomena from the microscopic molecular and atomic levels, and reveal the reaction details. Therefore, more and more chemists solve the chemical problems by using the combing of experiment with computational study. Based on this, our group had studied a series of reactions catalyzed by dirhodium via the theoretical study and explained the experimental phenomena very well. At now, we have previously reported [3+3]-, [3+2]-cycloadditions catalyzed by dirhodium carbene compound (Yang et al., 2014, 2016; Zhang et al., 2018), and other computational study on reactions catalyzed by rhodium. Hence, based on our previous research, we are interested in the reactions catalyzed by divergent dirhodium catalysts reported by Dolye group (Cheng et al., 2017). In their experiments (shown in Scheme 3), they detected the presence of certain intermediates, but the mechanism remained unclear. And the most important thing is that there is no specific data to verify the hypothesis mechanism proposed by the experiment author and explain the role of two different catalysts in the system. Therefore, for the deeper understanding of the reaction, we shall adopt DFT method to investigate the reaction mechanism and reveal the special functions of divergent catalysts to produce diverse outcomes in molecular level.

## Computational Details

The theoretical calculations were performed by the use of B3LYP density functional method (Lee et al., 1988; Becke, 1993a,b), augmented with D3 empirical dispersion correction (Grimme et al., 2010; Grimme, 2011). As for geometry optimization in the gas phase, the 6-31G (d, p) basis set was used for all non-metal elements (C, H, O, N, and Si), while the 1997 Stuttgart relativistic small-core effective core-potential combined with a 4f function [ζ_f_ (Rh) = 1.350] [Stuttgart RSC 1997 ECP+4f] for rhodium element (Kaupp et al., 1991; Bergner et al., 1993; Hansen et al., 2009). This mixed basis set is donated to BS1. In order to confirm the stationary points as minima without imaginary frequencies or the transition states with only one imaginary frequency, the harmonic frequency calculations were employed at the level of B3LYP-D3/BS1. Moreover, the transition states connected with correct reactants and appropriate products were verified by the intrinsic reaction coordinate (IRC) analysis (Fukui, 1970, 1981).

Because the M06 functional (Zhao and Truhlar, 2008; Deng et al., 2017; Wu et al., 2018; Zhang et al., 2018) is more accurate in energy calculation, the latter single-point energies were calculated for all the B3LYP-D3/BS1-optimized structures in chloroform (the solvent used experimentally, ε = 4.71) with the SMD solvation model (Marenich et al., 2009; Ribeiro et al., 2011), one typical model of polarisable continuum model (PCM; Miertuš et al., 1981; Tomasi et al., 2005) at the level of M06-D3/BS2. BS2 denotes a mixed basis set of 6-311+G(d,p) for all non-metal elements and [Stuttgart RSC 1997 ECP+4f] for rhodium atom. These M06-D3/SMD/BS2 electronic energies were then added to the free energy correction evaluated from the unscaled vibrational frequencies on the B3LYP-D3/BS1 optimized geometries in the gas phase at 298.15 K and 1 atm to obtain the Gibbs free energies in chloroform solvent. Nature bond orbital (NBO) analysis was done with the B3LYP-D3/BS1 method. All the DFT calculations were performed by the use of Gaussian 09 program (Frisch et al., 2009). And the non-covalent interaction (NCIplot) analysis was carried out with program Multiwfn (Lu and Chen, 2012).

## Results and Discussion

### Rh_2_(OAc)_4_-catalyzed [3+2]-cycloaddition

**Scheme 4:**
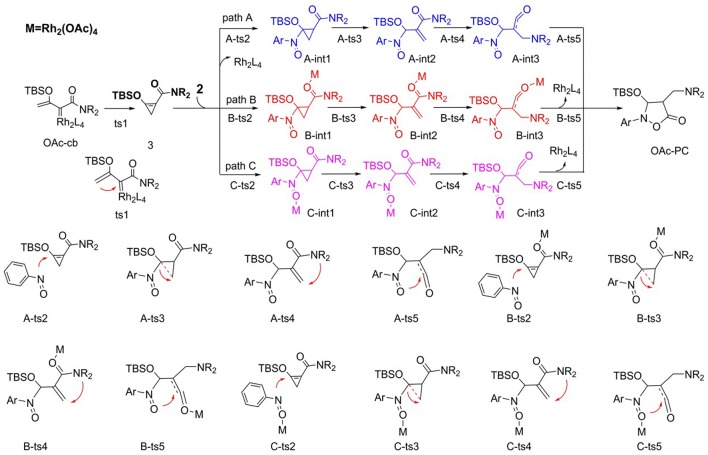
The supposed reaction mechanism of [3+2]-cycloaddition catalyzed by Rh_2_(OAc)_4_.

We first studied the [3+2]-cycloaddition reaction catalyzed by Rh_2_(OAc)_4_, for which the following mechanisms were hypothesized according to our previous study and the mechanism provided by the experimental author (Cheng et al., 2017), as shown in Scheme 4. There are three pathways, named paths A, B, and C, respectively, and the mechanism starts with the Rh_2_(OAc)_4_-carbene (**OAc-cb**) generated by the interaction of Rh_2_(OAc)_4_ with diazo compound **1**. Then the carbenoid comes into being the three-membered cyclopropene compound **3** by intramolecular cyclization via transition state **ts1**, which is the common first step of paths A, B, and C. By the way, it's worth mentioning that this cyclopropene **3** was detected in the [3+2]-cycloaddition process of Rh_2_(OAc)_4_-catalyzed diazo compound as the intermediate (Cheng et al., 2017), therefore, the reaction mechanism must go through this step. Next, three pathways for the following steps are taken into consideration after cyclopropane formation. In fact, the processes of these three pathways are the same, which are differentiated by the reaction sites of catalyst.

For path A, after the formation of cyclopropene, the dirhodium catalyst separated away, and there is no catalyst in the subsequent steps. The N atom of nitro of nitrosoarene **2** interacts with the C = C bond of cyclopropene compound via transition state **A-ts2** and then generates intermediate **A-int1**. Then the ring of three-membered intermediate breaks and turns into intermediate **A-int2** through **A-ts3** transition state. And the following step is NR_2_ group transferring to the vinyl group by the four-membered ring transition state **A-ts4** and then electron rearrangement gives **A-int3**. Finally, the ketene analog **A-int3** comes into being the five-membered ring product which goes through the interaction of O atom of nitro with C atom of C = O bond by the way of intramolecular ring closed. As mentioned earlier, the processes of path B and path C are similar to path A, but these two pathways both have the dirhodium catalyst interaction. In path B, the Rh_2_(OAc)_4_ interacts with the O atom of C = O group of **3**, which is adjacent with NR_2_ group from beginning to end. However, the dirhodium catalyst always reacts with the O atom of the nitro group in **2** in path C.

**Figure 1:**
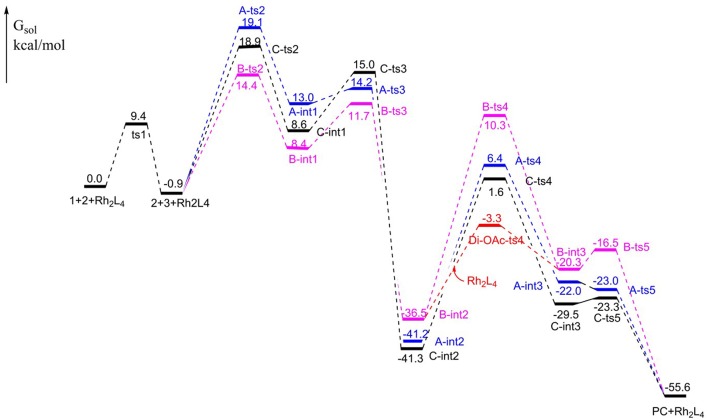
The free energy profiles for [3+2]-cycloaddition catalyzed by Rh_2_(OAc)_4_.

The free energy profiles of aforementioned pathways are depicted in Figure 1 and the corresponding free energy barriers are shown in Table 1. As shown in free energy profile, the blue line is path A, the pink color represents path B and the black one indicates path C. By the way, the energy barriers in Table 1 are more simple and clear. At first glance, the free energies of activation of **ts4** are too high among all three pathways. Separately, the generation of cyclopropene **3** from Rh_2_(OAc)_4_ carbene compound needs free energy barrier of 9.4 kcal/mol. For the step of cyclopropene addition, the free energy barrier is 20.0, 15.3, and 19.8 kcal/mol for paths A, B, and C orderly. Compared with the **ts2**, the free energy barrier of **ts3** in three pathways is much lower, being 1.2, 3.3, 6.4 kcal/mol, respectively, and this means that three-membered ring breaking is much easier to occur. However, the most difficult step is NR_2_ group transfer, with the high free energy barrier in 47.6, 46.8, 42.9 kcal/mol, respectively. Finally, owing to the high activity of ketene analog **A-int3**, it can quickly react to final product without free energy barrier (−1.0 kcal/mol) in path A, however, there need 3.8 and 6.2 kcal/mol free energy barriers in path B and path C. That's to say, the interaction of Rh_2_(OAc)_4_ with ketene analog **A-int3** can make it more stable. In addition, with the high free energy barrier of **ts4**, the transfer step of NR_2_ group cannot take place like this in all three pathways.

**Table 1:** The free energy barriers (Δ*G*^≠^, kcal/mol) of three pathways of Rh_2_(OAc)_4_-catalyzed [3+2]-cycloaddition reaction.

	**Free energy barrier** **Δ*****G***^****≠****^				
**Pathway**	**ts1**	**ts2**	**ts3**	**ts4**	**ts5**
A	9.4	20.0	1.2	47.6	−1.0
B	9.4	15.3	3.3	46.8	3.8
C	9.4	19.8	6.4	42.9	6.2
Di-OAc				**33.2**	

*Bold value used to highlight the optimal way*.

On the base of the previous calculation results, we have tried our best to decrease the free energy barrier of **ts4** formation and we find that the two molecule Rh_2_(OAc)_4_ catalyst, one interacting with O atom in nitro group and the other one reacting with O atom of C = O group, named **Di-OAc-ts4** (the red color in Figure 1), can make the barrier lower, with 33.2 kcal/mol (this is based on path B, and 38.0 kcal/mol on path C) which is the lowest barrier that we found (see some other transition state structures in Figure S1 of Supporting information). To sum up, the better pathway is path B with the rhodium catalyst interacting with the C = O group. Hence, the whole mechanism of [3+2]-cycloaddition catalyzed by Rh_2_(OAc)_4_ includes mainly five steps, formation of cyclopropane (**ts1**), cyclopropene addition (**B-ts2**), three-membered ring open (**B-ts3**), NR_2_ group transfer (**Di-OAc-ts4**), and five-membered outcome generated (**B-ts5**), and the process of NR_2_ group transfer with two molecular catalyst to stable the reaction system is the rate-limiting step.

**Figure 2:**
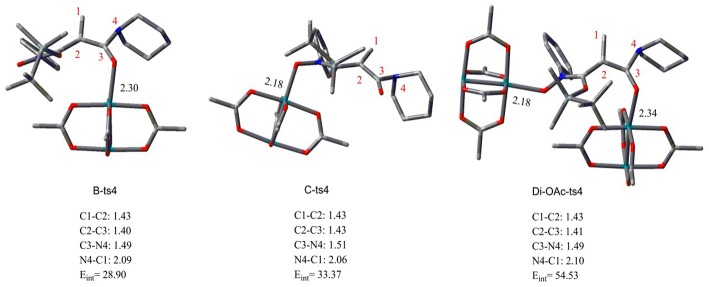
The structures of transition states **B-ts4**, **C-ts4**, and **Di-OAc-ts4** (bond length in Å and energy in kcal/mol).

Aimed at the NR_2_ group transfer step, the structures of the **B-ts4**, **C-ts4**, and **Di-OAc-ts4** are compared clearly, the results are vividly depicted in Figure 2. In order to make the structure more distinct, we just show the molecular skeleton. Firstly, in terms of reaction center, the Rh_2_(OAc)_4_ catalyst has little effect on it, and the bond length of the reaction center hardly changes. However, the distances of Rh-O in **B-ts4**, **C-ts4**, and **Di-OAc-ts4** are more different. So as you see from Figure 2, the Rh-O bond of **B-ts4** is 2.30 Å, **C-ts4** is 2.18 Å, and two bond lengths in **Di-OAc-ts4** are 2.18 Å and 2.34 Å as well. In terms of the bond length, the shorter bond in **C-ts4**, we can get that, suggests that there is a stronger interaction in **C-ts4** than in **B-ts4**. And the relative free energy also accords with the result, that the free energy of **C-ts4** is 8.7 kcal/mol lower than **B-ts4**. Moreover, the value of interaction energy (*E*_int_) also verifies the conclusion. And *E*_int_ is 28.90 kcal/mol and 33.37 kcal/mol in **B-ts4** and **C-ts4**, respectively. Noticeably, the interaction energy of two-molecule catalysts in **Di-OAc-ts4** is 54.53 kcal/mol, it can be seen that the **Di-OAc-ts4** is the most stable one with the huge interaction energy.

### Rh_2_(cap)_4_-catalyzed [5+1]-cycloaddition

**Scheme 5:**
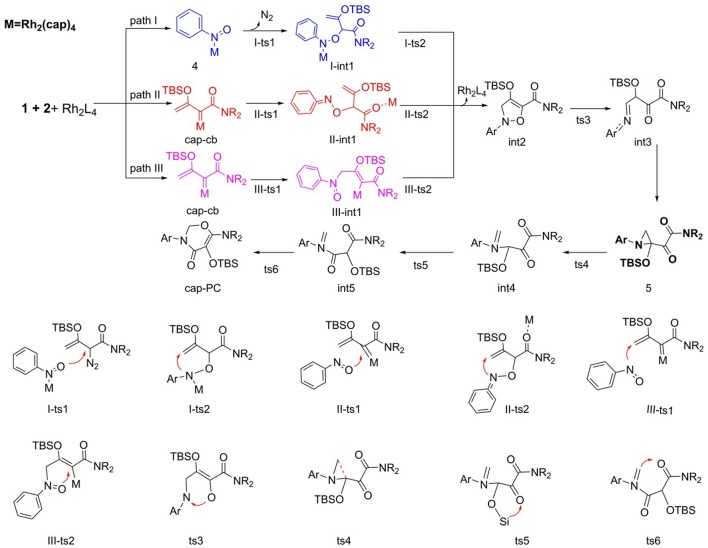
The supposed reaction mechanism of [5+1]-cycloaddition catalyzed by Rh_2_(cap)_4_.

Regard to mechanism of the [5+1]-cycloaddition reaction catalyzed by Rh_2_(cap)_4_, we put forward the following pathways, as shown in Scheme 5. In this part, start with reactants (**1** and **2**), and Rh_2_(cap)_4_ catalyst. Here, the [5+1]-cycloaddition reaction may generate five-membered ring intermediate **int2** and then expands into a six-membered ring product. Hence, we hypothesized three different pathways in the process of **int2** formation and one path to ring enlargement.

As to the formation of five-membered ring intermediate **int2**, three pathways are path I, path II, and path III, respectively. For path I, firstly, the Rh_2_(cap)_4_ interacts with N atom of nitro of reactant **2** to form compound **4**. Then the O atom of nitro in **4** reacts with the diazo group of reactant **1**, and nitrogen goes away to generate **A-int1** by the transition state **I-ts1**. Next, the N atom of nitro attacks the terminal alkenyl leading to the formation of the five-membered ring intermediate **int2** by the transition state **I-ts2**. Obviously, the other two ways are more different, and they begin with the **cap-cb** generated by the interaction of Rh_2_(cap)_4_ with reactant **1**. In Path II, the first step is that O atom of nitro reacts with carbene center via transition state **II-ts1**, and catalyst transfers to interact with C = O group to form **II-int1**. Then N atom attacks the terminal alkenyl group to produce **int2** through five-membered ring closed, which called carbene addition step. Differently, the order in path III is reverse. Firstly, the N atom of nitro interacts with the terminal alkenyl group via **III-ts1**, and then ring closes to form **int2** which we called alkenyl addition step. During the formation of **int2** by closed-ring process, the dirhodium catalyst leaves away the reaction system.

As shown in Scheme 5, after the various ways to **int2**, the initial N-O bond will break to generate **int3** by N-O bond heterolysis. Next, the intermediate **int3** goes directly to compound **5**, which was detected in the experiment (Cheng et al., 2017). And the following step is three-membered ring open to turn into **int4** with the transition state **ts4**. Then, the TBS (*tert*-butyldimethylsilyl) group transfers to the adjacent carbonyl group by the way of five-membered ring transition state **ts5**. Finally, the alkenyl group formed by three-membered ring open reacts with the carbonyl group connected with NR_2_ group to give the six-membered ring product. Owing to the departure of the Rh_2_(cap)_4_ catalyst in the formation of **int2**, the steps from **int2** to final product are no catalyst interaction.

**Figure 3:**
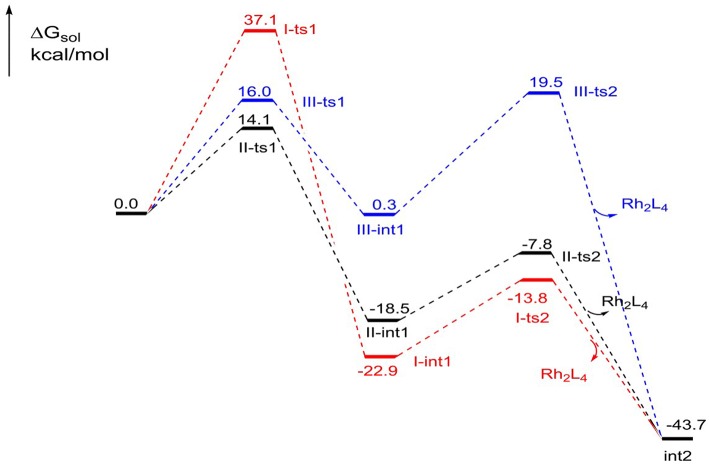
The free energy profiles of three pathways in the formation of the five-membered ring compound **int2**.

The free energy profiles of the three foregoing addition pathways to **int2** formation are shown in Figure 3. The red, black, and bluelines represent path I, path II, and path III, respectively. At first glance, the step of N_2_ away has high energy barrier in 37.1 kcal/mol in path I, and the next intramolecular closed-loop step is in 9.1 kcal/mol. Compared to other two ways, it is more difficult to take place. For the path II, the free energy barrier of carbene center addition step is 14.1 kcal/mol, and it is 23.0 kcal/mol lower than **I-ts1**, and 1.9 kcal/mol lower than **III-ts1**. Moreover, the closed-ring step owns a free energy barrier of 10.7 kcal/mol. Meanwhile, the free energy barriers of the two steps in third path are 16.0 and 19.2 kcal/mol, respectively, and the second step is 8.5 kcal/mol higher than **II-ts2** in path II. By comparison, for the formation of the five-membered intermediate **int2**, the path II is the most favorable way with the lowest free energy barrier. This result overturns the experiment author's hypothesis, which suggests the mechanism is going like path I (Cheng et al., 2017).

The relative free energies to separated reactants for the intermediates and transition states in subsequent steps are shown in Table 2. As we can see, the free energy barrier from **int2** to **ts3**, five-membered ring open, is 20.5 kcal/mol. It's worth noting that there is no free energy barrier from **int3** to three-membered N-heterocycle compound **5**. The scanning of this process was performed and no transition state was located in its potential energy surface. Next, the ring-opening of **5** has a free energy barrier of 22.1 kcal/mol. What's more, the free energy barrier of TBS group transfer is in 16.2 kcal/mol and the formation of final six-membered product needs the free energy barrier of 5.7 kcal/mol. In brief, among the free energy barrier values of the whole reaction mechanism, the ring-breaking step of N-heterocycle compound **5** (from **5** to **int4** via **ts4** in Scheme 5) has the most high free energy barrier, 22.1 kcal/mol, and is the rate-limiting step of the [5+1]-cycloaddition catalyzed by Rh_2_(cap)_4_. By the way, the step of catalyst-deciding is carbene addition.

**Table 2:** The relative free energies (Δ*G*, kcal/mol) of intermediates and transition states (from **int2** to **PC**) to separated reactants.

**Relative free energies**					
**Structure**	**int2**	**ts3**	**int3**	**5**	**ts4**
Δ*G*	−47.3	−26.8	−33.6	−76.8	−54.7
**Structure**	**int4**	**ts5**	**int5**	**ts6**	**PC**
Δ*G*	−62.4	−46.2	−62.3	−56.6	−80.33

**Figure 4:**
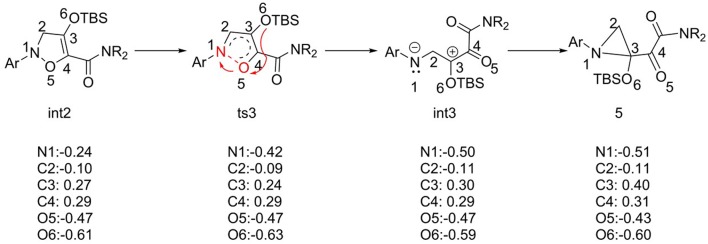
The NBO charge analysis of structures from **int2** to compound **5**.

For the barrier-free process from **int3** to **5**, the natural bond orbital (NBO) analysis was carried out, and the charge changes from **int2** to compound **5** are shown in Figure 4. According to charge changes, the results suggest that the process of N-O bond breaking goes through the heterolysis way, and the electrons transfer to N1 atom. This way makes the charge of N atom more negative (−0.26 e^−^), and O5 atom positive. And then electron rearrangement, electrons transfer from O6 atom to O5 atom, makes charge of O5 change invariant. However, the intermediate **int3** is not stable enough, and it comes into being compound **5**, with the huge energy release of 43.2 kcal/mol, which is the interaction of positive and negative charges. We speculated that the most possible reason is the difference in stability between the two structures.

### Comparison of Dirhodium(II)-carbene Formation Steps From Rh_2_(OAc)_4_ and Rh_2_(cap)_4_

For aforementioned [3+2]-cycloaddition and [5+1]-cycloaddition reactions, there is bewilderment unknown: why the different catalysts can obtain different products for the same reactants. In order to understand the origin of catalyst-dependent chemoselectivity in these dirhodium-catalyzed cyclization reactions, we will analyze and compare the two [3+2]-cycloaddition and [5+1]-cycloaddition reactions catalyzed by different catalysts Rh_2_(OAc)_4_ and Rh_2_(cap)_4_ in several ways.

**Figure 5:**
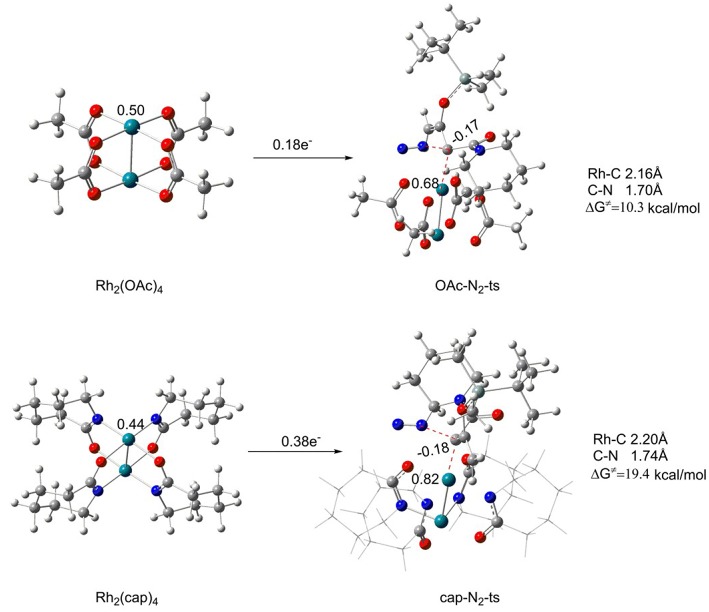
The initial carbenoid formation step of Rh_2_(OAc)_4_ and Rh_2_(cap)_4_.

Firstly, the Rh_2_(II)-carbene formation step is taken into consideration. In this process, reactant **1** reacts with different catalysts Rh_2_(OAc)_4_ and Rh_2_(cap)_4_, respectively, to form the corresponding Rh_2_(II)-carbene with the elimination of N_2_ molecule. The free energy barriers and transition structures are shown in Figure 5. Beginning with the two catalysts, the charges of center rhodium are different on account of the electronic effect differences of the ligands in catalysts. And the charge of center rhodium in Rh_2_(OAc)_4_ is 0.50, while 0.44 for Rh_2_(cap)_4_. Then the catalyst reacts with reactant **1**, the formation of transition state **OAc-N**_**2**_**-ts** (for Rh_2_(OAc)_4_) needs the free energy of activation (Δ*G*^≠^) of 10.3 kcal/mol, and the free energy barrier of **cap-N**_**2**_**-ts** (for Rh_2_(cap)_4_) is 19.4 kcal/mol, which is 9.1 kcal/mol higher than the former. There is little difference between the bond lengths of the reaction centers in transition states. The bond distances of Rh-C and C-N bonds are 2.16 Å and 1.70 Å in **OAc-N**_**2**_**-ts**, respectively. Similarly, the Rh-C bond is 2.20 Å, and C-N bond is 1.74 Å for transition state **cap-N**_**2**_**-ts**. However, the main distinction is the electron transfer number. During the course of Rh_2_(OAc)_4_ becoming to **OAc-N**_**2**_**-ts**, there is 0.18 e^−^ (from 0.50 to 0.68) transferring from catalyst to reactant **1**. Moreover, the electron transfer number is 0.38 e^−^ in the Rh_2_(cap)_4_ catalyzed process. So it can be concluded that the dirhodium catalyst in this step is in the type of electron donor. Hence, the electronic effect of the ligand in catalyst is most important for the catalyst activity.

### [5+1]-cycloaddition Reaction Catalyzed by Rh_2_(OAc)_4_

**Figure 6:**
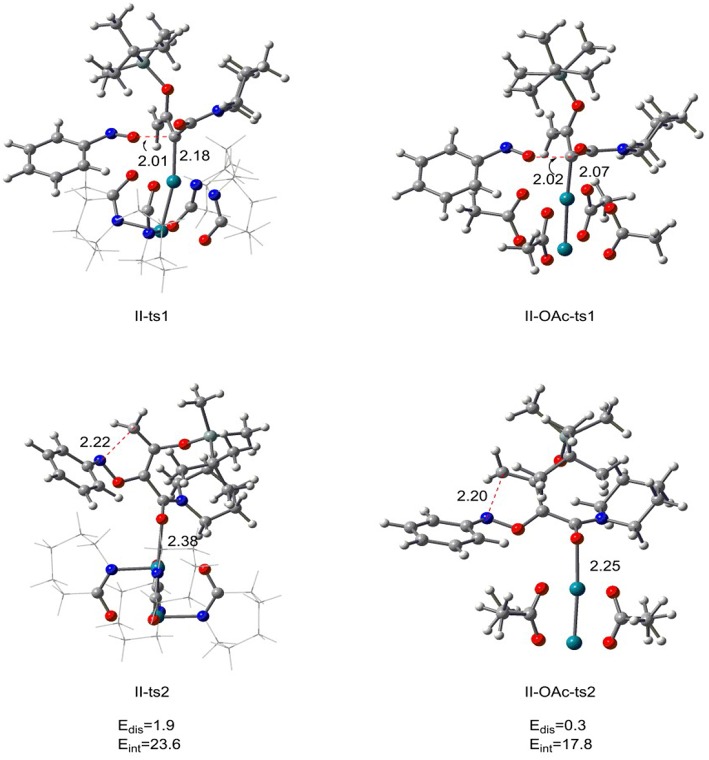
The transition state structures of **II-ts1**, **II-ts2** in [5+1]-cycloadditioncatalyzed by Rh_2_(cap)_4_ and **II-OAc-ts1**, **II-OAc-ts2** in **[5+1]-**cycloadditioncatalyzed by Rh_2_(OAc)_4_ along with the distortion/interaction analysis (bond length is in Å, energies are given in kcal/mol).

From the previous discussion, we know that the optimal catalyst interaction pathway of Rh_2_(cap)_4_-catalyzed [5+1]-cycloaddition reaction is carbene addition way (path II). Therefore, in order to compare the two different catalysts, we changed the Rh_2_(cap)_4_ with Rh_2_(OAc)_4_ in carbene addition step, and the results are displayed in Figure 6. For the first step, the relative free energy of **II-OAc-ts1** is 10.4 kcal/mol which is 3.7 kcal/mol lower than **II-ts1** (14.1 kcal/mol) in [5+1]-cycloaddition reaction catalyzed by Rh_2_(cap)_4_, and this computational result suggests that **II-OAc-ts1** is more stable than **II-ts1**. And this difference is caused by the bond length of Rh-C bond. In Figure 6, the Rh-C bond distance is 2.18 Å in **II-ts1** structure, and 2.07 Å in **II-OAc-ts1**, and the shorter bond means stronger interaction, which makes transition state **II-OAc-ts1** more stable.

However, the bond length is inconsistent with the stability in the five-membered ring closed step. The Rh-O bond of **II-ts2** is 2.38 Å, and 2.25 Å in **II-OAc-ts2**, but the relative free energies of **II-ts2** and **II-OAc-ts2** are −7.8 and 7.0 kcal/mol, respectively, which suggests that transition state **II-ts2** is more stable than **II-OAc-ts2**. In addition, the distortion/interaction analysis (Zhang et al., 2018) was adopted to explain this phenomenon. *E*_dis_ means the total distortion energy [combining catalyst part with reaction part (the system except rhodium catalyst)], and *E*_int_ represents the interaction energy between the catalyst and reaction part. Comparing the distortion energies between two transition states **II-ts2** and **II-OAc-ts2**, there is very small difference of 1.6 kcal/mol. However, the interaction energy *E*_int_ of **II-ts2** is 23.6 kcal/mol, 5.8 kcal/mol higher than that of **II-OAc-ts2**. The stronger interaction between the rhodium catalyst and reaction part in **II-ts2** than in **II-OAc-ts2** makes **II-ts2** more stable, with 14.8 kcal/mol lower than **II-OAc-ts2** in free energy. That's to say, the Rh_2_(cap)_4_ is more conducive to the formation of **int2**, so as to complete the subsequent ring enlargement to form six-element ring products. In other words, this computational result reveals it is Rh_2_(cap)_4_ that catalyzes [5+1]-cycloaddition.

### [3+2]-cycloaddition Reaction Catalyzed by Rh_2_(cap)_4_

**Figure 7:**
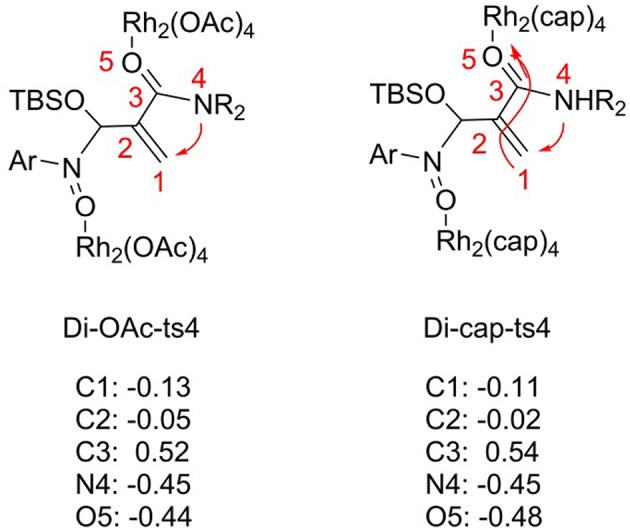
The NBO charges of **Di-OAc-ts4** and **Di-cap-ts4**.

**Figure 8:**
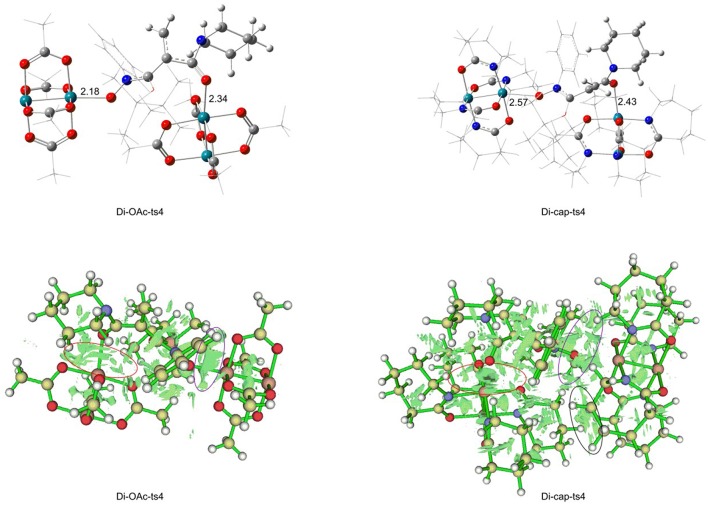
The non-covalent interaction analysis of Di-OAc-ts4 and Di-cap-ts4.

For the [3+2]-cycloaddition catalyzed by Rh_2_(OAc)_4_, the rate-limiting step is NR_2_ group transfer with two molecular catalysts interacted. In order to explore the catalyst role, we changed the Rh_2_(OAc)_4_ with Rh_2_(cap)_4_, and the transition state named **Di-cap-ts4**. The electronic effect and steric hindrance effect analyses are considered, and the results are shown in Figures 7, 8. In terms of electronic effect, the results in Figure 7 suggest that the charge of reaction center with tiny change is caused by the different catalysts. By comparing the direction of electron flow, we can get that the Rh_2_(cap)_4_ catalyst acts as the electron acceptor with the charges of C1, C2, C3 atoms becoming positive, and O5 charge being more negative, as shown by the arrow in **Di-cap-ts4**. By the way, the more negative C1 atom is, the more easy NR_2_ group transfer.

And then the non-covalent interaction (NCIplot) analysis was carried out, the results are shown in Figure 8. Firstly, the bond length of two Rh-O bonds should be illustrated. The Rh-O bond of nitro in **Di-OAc-ts4** structure is 2.18 Å, and the Rh-O bond of carbonyl is 2.34 Å, while the bond length is 2.57 Å, and 2.43 Å in **Di-cap-ts4**, respectively, which shows the **Di-OAc-ts4** is more stable than the one in Rh_2_(cap)_4_ replaced with two shorter bonds. Meanwhile, the result of steric hindrance effect is consistent with the previous conclusion. The steric hindrance of **Di-cap-ts4** is larger than **Di-OAc-ts4**, furthermore, there is a space repulsion between the two molecular catalysts in **Di-cap-ts4** and this repulsion effect main depends on the larger cap ligand. Hence, the huge steric hindrance makes it unstable, in other words, Rh_2_(OAc)_4_ catalyst is more favorable for [3+2]-cycloaddition reaction, and this theoretical result explains why no five-membered ring product appeared when Rh_2_(cap)_4_ was used as the catalyst.

## Conclusion

The DFT B3LYP-D3 method has been employed to investigate reaction mechanism of cyclization reactions between enoldiazoacetamides and nitrosoarens catalyzed by divergent rhodium catalysts. When Rh_2_(OAc)_4_ acts as the catalyst, among three pathways considered, path B with catalyst interacting with O = C group is the most feasible way, and the NR_2_ group transfer step involved in two-molecule Rh_2_(OAc)_4_ catalyst is the rate-limiting step. While, the most favorable path is path II, carbene addition, when the Rh_2_(cap)_4_ was the catalyst, and the N-heterocycle compound ring open step is the rate-limiting step. By the way, the catalyst-deciding step is inconsistent with the rate-limiting step. The important effects of catalyst on this reaction system is mainly due to electronic effect and steric hindrance effect of the ligand in divergent catalysts. For the initial carbene formation step, Rh_2_(cap)_4_ is harder than Rh_2_(OAc)_4_ owing to the electronic effect. The Rh_2_(cap)_4_ cannot catalyze [3+2]-cycloaddition reaction also due to both electronic effect and steric hindrance effect, because the huge steric hindrance between the ligands on the catalyst. Meanwhile, the Rh_2_(OAc)_4_ will not catalyze [5+1]-cycloaddition on account of the stability of transition structure, which decided by the interaction between catalyst, and reaction part. The mechanisms of cycloaddition reactions catalyzed by divergent dirhodium, and the important role of catalysts are firstly studied in detail in this computation paper, which can take on deep understanding of cycloaddition reaction catalyzed by diverse dirhodium catalysts.

## Data Availability

All datasets generated for this study are included in the manuscript and/or the Supplementary File.

## Author Contributions

YX designed the research. YZ, YY, and RZ were involved in the calculations. YZ, YY, and XW analyzed the data. YZ and YX wrote the manuscript.

### Conflict of Interest Statement

The authors declare that the research was conducted in the absence of any commercial or financial relationships that could be construed as a potential conflict of interest.
