# Supplementary material for: Catalyst-Dependent Chemoselectivity in the Dirhodium-Catalyzed Cyclization Reactions Between Enodiazoacetamide and Nitrosoarene: A Theoretical Study
Source: Front Chem. 2019 Aug 23;7:586. doi: 10.3389/fchem.2019.00586 (PMC6716548; doi:10.3389/fchem.2019.00586)
Supplement: Supplementary file 1 [file Data_Sheet_1.docx]

*Supporting information*

**Catalyst-****dependent chemoselectivity in the rhodium-catalyzed cyclization reactions between enodiazoacetamide and nitrosoarene: a theoretical study**

Yan Zhang, Yongsheng Yang, Ruyu Zhu, Xingyu Wang, Ying Xue^*^

College of Chemistry,

Key Lab of Green Chemistry and Technology in Ministry of Education,

Sichuan University, Chengdu 610064,

People’s Republic of China

_________________

Corresponding author. Ying Xue, e-mail: [yxue@scu.edu.cn](mailto:yxue@scu.edu.cn)

Tel: +86 28 85418330.





**Figure S1**. The other two transition states of NR_2_ transfer in Rh_2_(OAc)_4_-catalyzed [3+2]-cycloaddition.

To decrease the energy barrier of NR2 group transfer, we had tried our best to find the possible transition states. There are the other two interaction ways, except the pathways in the text part, as shown in Figure S1. One way has two molecular A-int2s, and the NR_2_ group transfers to the alkenyl group of each other with the eight-membered transition state generating. However, the free energy barrier is so high in 42.0 kcal/mol. Meanwhile, we also stumbled upon a transition state like OAc-ts4-2, but the free energy barrier is also high in 41.3 kcal/mol. Compared with the Di-Rh-ts4, these pathways are difficult to take place.

**Structure coordinates of intermediates and transition states**

1

0 1

C -0.89887100 1.21300600 -0.19159500

C 0.55277700 1.50274300 -0.19632400

C 1.08028800 2.63141300 -0.69258600

O 1.28004600 0.53700800 0.42606500

H 2.13849300 2.83602300 -0.59609400

H 0.46030400 3.36135000 -1.19889900

Si 2.36823600 -0.58309100 -0.25969300

C 2.43091700 -0.33408600 -2.11773400

H 3.06694600 -1.09199500 -2.58722100

H 2.80933200 0.65338400 -2.39554500

H 1.41636600 -0.43621100 -2.51275900

C 4.04877600 -0.23715300 0.57974200

C 1.73712600 -2.29394400 0.18816300

H 2.44393300 -3.07361900 -0.11654100

H 0.78334500 -2.46641200 -0.31668600

H 1.57830700 -2.38618300 1.26736700

C 5.11843400 -1.17583000 -0.01726300

H 6.09075400 -1.00397700 0.46442400

H 5.25390700 -1.01038600 -1.09231400

H 4.86641000 -2.23243600 0.13073600

C 3.93427400 -0.48907600 2.09725000

H 3.68231900 -1.53174200 2.31921400

H 3.16733300 0.14718100 2.55249700

H 4.88824800 -0.26921300 2.59634800

C 4.47038500 1.22758700 0.34866800

H 5.45503300 1.41865800 0.79725300

H 3.75994700 1.92274400 0.80786500

H 4.54551900 1.47239000 -0.71727900

N -2.61455900 -0.52829100 0.06437900

C -3.20319300 0.01599300 1.29187300

H -3.12917600 -0.75616200 2.07346200

H -2.62679600 0.87056700 1.64218600

C -4.67726100 0.37043500 1.06652600

H -4.73805700 1.18658200 0.33595900

H -5.11197400 0.73717100 2.00307700

C -5.44555000 -0.85767800 0.55162700

H -6.48377500 -0.59298000 0.32420800

H -5.47887000 -1.61594100 1.34684600

C -4.75899800 -1.45583600 -0.68548400

H -4.83167600 -0.75227400 -1.52430300

H -5.25760500 -2.38171200 -0.99393400

C -3.27575900 -1.74441700 -0.41853600

H -2.75569700 -2.07776800 -1.31594000

H -3.17381300 -2.53518000 0.34019200

C -1.42065600 -0.15331400 -0.49460900

O -0.79103500 -0.87398100 -1.27012100

N -1.74420800 2.20499000 -0.13637300

N -2.50446000 3.05874800 -0.08576000

2

0 1

O -1.35905800 2.39136800 0.00000000

C 0.00000000 0.59941900 0.00000000

C 1.33298700 0.17695000 0.00000000

C -1.05412100 -0.32578200 0.00000000

C 1.62100400 -1.18682100 0.00000000

H 2.11561400 0.92936300 0.00000000

C -0.76052600 -1.68294700 0.00000000

H -2.07211000 0.04865700 0.00000000

C 0.57489800 -2.11250400 0.00000000

H 2.65134300 -1.52816200 0.00000000

H -1.56329100 -2.41413600 0.00000000

H 0.79646500 -3.17581100 0.00000000

N -0.19157200 2.02846500 0.00000000

3

0 1

C 0.68382700 0.92745900 -0.43299600

C -0.46862500 1.54451700 -0.33991800

C 0.59094400 2.37179400 -0.96475700

O -1.73677400 1.62032400 -0.00185700

H 1.04666800 3.19406600 -0.40073600

H 0.57134800 2.55348800 -2.04512900

Si -2.67451800 0.36615000 0.74755100

C -4.12401600 1.32574200 1.45640200

H -4.87988100 0.64257800 1.85940300

H -4.60283800 1.94310100 0.69069700

H -3.79641200 1.98180000 2.26884900

C -3.22824700 -0.83895100 -0.62071600

C -1.63874700 -0.43186600 2.08417400

H -2.22887500 -1.17832500 2.62672300

H -1.30282600 0.31925600 2.80733900

H -0.75970000 -0.92019600 1.65337700

C -3.81136600 -2.10453500 0.04689200

H -4.18138200 -2.79673000 -0.72128000

H -4.65653500 -1.87539100 0.70819100

H -3.05677700 -2.63955500 0.63295300

C -2.03496700 -1.24156300 -1.51328500

H -1.20171800 -1.65631300 -0.93882700

H -1.64926500 -0.38451300 -2.07554500

H -2.35720200 -1.99496200 -2.24523200

C -4.31510600 -0.17788300 -1.49413400

H -4.61431000 -0.85861200 -2.30250100

H -3.95502000 0.74612600 -1.96074200

H -5.21635700 0.06371400 -0.92012400

N 2.76417200 -0.33271100 -0.59396200

C 3.59066600 0.84541300 -0.85707500

H 4.17904200 0.65140300 -1.76560900

H 2.95540500 1.70471800 -1.05583200

C 4.53940200 1.11571900 0.32145400

H 3.93685400 1.39583000 1.19515600

H 5.18478100 1.96948700 0.08296500

C 5.37940800 -0.12947500 0.64481300

H 6.00093400 0.04655700 1.52986200

H 6.06751500 -0.32302500 -0.19074000

C 4.48090400 -1.35928900 0.84980800

H 3.87176600 -1.23156600 1.75345700

H 5.08628800 -2.26232800 0.99066900

C 3.53756800 -1.55225800 -0.34612300

H 2.83559000 -2.36747800 -0.17758100

H 4.12195900 -1.77257000 -1.25071900

C 1.43400600 -0.31193000 -0.24928300

O 0.86191000 -1.32595400 0.18078800

4

0 1

Rh 0.60621900 -1.66659600 -0.40157400

C 2.27690700 0.19597700 -1.89701400

N 1.55735500 1.15820000 -1.34854300

O 2.02228600 -1.05633600 -1.76913600

C 3.53544700 0.50070600 -2.69854300

H 3.82473400 -0.43971800 -3.17180300

H 3.32354300 1.22257300 -3.49701200

C 4.68773600 1.02562100 -1.81543900

H 5.63189500 0.88312300 -2.35495700

H 4.75235200 0.40389000 -0.91443100

C 4.56495800 2.50582900 -1.42683300

H 4.63760000 3.10959100 -2.34320800

H 5.42726500 2.78583200 -0.80814100

C 3.26705900 2.88992800 -0.70199000

H 3.19619000 2.39702400 0.27454700

H 3.28559600 3.97071200 -0.50991500

C 1.98936100 2.55398300 -1.48888500

H 2.12650400 2.80841400 -2.55076300

H 1.17367600 3.18165100 -1.14314000

C 1.96600100 -0.20970200 1.74722400

N 1.95516900 -1.34560400 1.09465800

O 1.14319100 0.75607100 1.49270000

C 2.98177500 0.08995000 2.83799700

H 2.68031000 1.03540600 3.29388700

H 2.93525800 -0.68246200 3.61734600

C 4.42438000 0.19433800 2.29981000

H 5.03049900 0.73452400 3.03695400

H 4.42046300 0.81054300 1.39287400

C 5.09364300 -1.15842100 2.01675300

H 6.12020800 -0.98146200 1.67219700

H 5.18028300 -1.70677100 2.96640100

C 4.37022200 -2.05778400 1.00391100

H 4.93720000 -2.99255200 0.90499600

H 4.34583100 -1.59565300 0.01074800

C 2.92475900 -2.40323800 1.39250800

H 2.87808100 -2.68716800 2.45555800

H 2.60706500 -3.28504000 0.82741000

C -1.74491800 -1.29704700 1.28417900

N -0.83295500 -2.17796000 0.95164200

O -1.69310100 -0.06011700 0.91996900

C -2.98286300 -1.66873200 2.08343400

H -2.69540600 -2.16928800 3.01763700

H -3.47512500 -0.73110700 2.35023400

C -3.94449500 -2.57405800 1.28518800

H -4.05904000 -2.16119100 0.27498000

H -4.93517700 -2.52813200 1.75336400

C -3.50872400 -4.04490600 1.20446600

H -4.26675300 -4.60888900 0.64666000

H -3.50491100 -4.46141000 2.22283100

C -2.12979800 -4.29121500 0.57369000

H -1.93903300 -5.37230000 0.56773800

H -2.10950400 -3.95012500 -0.46840400

C -0.97861800 -3.58863500 1.31422600

H -0.03139600 -4.07649300 1.06285100

H -1.10175900 -3.70677500 2.40229100

C -1.41803100 -0.92019800 -2.32561800

N -1.17642700 0.31562200 -1.92898600

O -0.81254500 -1.95114900 -1.86094200

C -2.51945800 -1.22610800 -3.33091200

H -2.32336500 -0.72728600 -4.28857300

H -2.47348600 -2.30201000 -3.51163900

C -3.91750600 -0.83126700 -2.80442700

H -3.99990400 -1.15128400 -1.75781700

H -4.66978500 -1.40103800 -3.36317500

C -4.25712000 0.66352200 -2.91892300

H -5.28151300 0.82291500 -2.55908500

H -4.26115500 0.93464400 -3.98490800

C -3.29949000 1.61214600 -2.18257800

H -3.60754900 2.64805800 -2.37641900

H -3.35736900 1.45485300 -1.09816700

C -1.83527100 1.43040400 -2.61223900

H -1.26166900 2.32918500 -2.39432200

H -1.78041500 1.29584000 -3.70150900

Rh -0.09018600 0.66160000 -0.19554900

O -0.87802000 3.51089000 -0.63840900

C -1.66248900 2.85515000 1.35912700

C -1.35088400 2.20349400 2.56008700

C -2.72438300 3.77146800 1.28394900

C -2.10700600 2.48996000 3.69525000

H -0.51683200 1.51539700 2.59051300

C -3.48727500 4.02241200 2.41716300

H -2.93939200 4.25199400 0.33694000

C -3.17705900 3.38478700 3.62550000

H -1.86415400 2.00459800 4.63566900

H -4.32410600 4.71223800 2.36396700

H -3.77315000 3.58713300 4.51088100

N -0.88310300 2.58370500 0.17960800

5

0 1

O -0.81050000 -2.79850600 0.45855500

C 0.04129800 1.91347500 0.30955000

C 0.50598300 2.90501400 1.17834400

C 0.29086300 2.01380300 -1.06685700

C 1.24020000 3.98037700 0.67258200

H 0.29387400 2.83779900 2.24066900

C 1.03260100 3.08366500 -1.55809400

H -0.10871200 1.24768100 -1.72292300

C 1.51482600 4.07043400 -0.69188300

H 1.60147800 4.74656400 1.35250100

H 1.23122100 3.15117600 -2.62396300

H 2.09255900 4.90403200 -1.07901600

N -0.73216800 0.81191000 0.74670500

C -1.01055800 -1.61534100 0.26978000

C -0.14772800 -0.52645200 0.87549800

C -0.64334000 0.23353500 2.07632100

O 1.20944300 -0.68843100 0.84165100

H -1.58723500 0.00910100 2.56806700

H 0.16438400 0.55071900 2.73545700

Si 2.30499800 -1.37287400 -0.26891600

C 1.79127300 -1.03571900 -2.04265400

H 2.38232200 -1.65855900 -2.72354400

H 1.95447000 0.01178000 -2.30936700

H 0.73241100 -1.25705000 -2.20666600

C 3.93550900 -0.49617000 0.18323300

C 2.40088600 -3.22190700 0.05048200

H 3.19903800 -3.68145500 -0.54321400

H 1.45427800 -3.70345100 -0.20754100

H 2.59966200 -3.42863100 1.10632600

C 5.04876900 -0.98208800 -0.76887300

H 6.00007800 -0.49001100 -0.52487200

H 4.82100300 -0.74869500 -1.81534500

H 5.21370600 -2.06332300 -0.69315400

C 4.32302300 -0.83063700 1.63874200

H 4.49581900 -1.90319800 1.78299100

H 3.54295300 -0.51765700 2.34077500

H 5.25024300 -0.31004900 1.91535900

C 3.77733800 1.03206600 0.04702800

H 4.72219000 1.53271700 0.30041600

H 3.00231700 1.42154500 0.71294400

H 3.51178800 1.33221900 -0.97146400

N -3.36224000 -1.14800300 -0.44445400

C -3.85445800 -1.25852500 0.92932500

H -4.67955200 -1.98289400 0.93970400

H -3.06593100 -1.66688100 1.56536400

C -4.34175700 0.10911300 1.43131800

H -3.47892800 0.78335600 1.48885500

H -4.75667500 0.00335100 2.44038700

C -5.38395700 0.70016500 0.46865700

H -5.68369300 1.69937400 0.80249900

H -6.28899600 0.07603900 0.48624900

C -4.83305300 0.75609800 -0.96484600

H -3.99227500 1.45880900 -1.01299900

H -5.59952100 1.11186200 -1.66255500

C -4.33448800 -0.62472800 -1.41129200

H -3.84571500 -0.58569300 -2.38478900

H -5.17035500 -1.33458200 -1.46699900

C -2.04726200 -1.12852200 -0.75412000

O -1.58058400 -0.81794400 -1.85202500

A-int1

0 1

C 1.19424500 -0.33394700 0.44496500

C -0.21223700 -0.25721500 0.63055800

C 0.64787600 -1.08521500 1.58217200

O -1.22737900 -0.72142900 -0.17630800

H 0.55755000 -2.16893000 1.48228400

H 0.75097700 -0.73401200 2.61482400

Si -2.81832900 -0.94695800 0.40390100

C -3.62310900 0.73518700 0.65241500

H -4.68498700 0.62830300 0.89900100

H -3.54272500 1.35224800 -0.24758500

H -3.14114500 1.27380500 1.47412400

C -3.64431700 -1.94032600 -0.98882600

C -2.73387100 -1.87158700 2.03874300

H -3.73469100 -2.04388000 2.44876200

H -2.16843500 -1.27852400 2.76500900

H -2.23904400 -2.84175300 1.93027900

C -5.11322000 -2.23397400 -0.62028600

H -5.59840800 -2.80934000 -1.42016200

H -5.69434400 -1.31462800 -0.48337400

H -5.19530100 -2.82407100 0.30013300

C -2.88904900 -3.27023700 -1.19311900

H -2.91899700 -3.89910800 -0.29581900

H -1.83912700 -3.09782500 -1.45062600

H -3.34530600 -3.84540200 -2.01019600

C -3.59308500 -1.12411900 -2.29769600

H -4.04351000 -1.69566300 -3.12057000

H -2.56268600 -0.88572300 -2.58032000

H -4.14660600 -0.18199800 -2.21393700

N 3.56904000 -0.15134200 -0.01864200

C 3.97024100 -0.91431400 1.15974300

H 4.71567800 -0.32301700 1.71281300

H 3.11692500 -1.05430200 1.81909200

C 4.59024900 -2.26006400 0.75258700

H 3.81160300 -2.87513600 0.28317000

H 4.93338400 -2.79297600 1.64743400

C 5.75010400 -2.04560800 -0.23178600

H 6.14761400 -3.00839300 -0.57213500

H 6.57054700 -1.53263400 0.29061000

C 5.30409200 -1.19064500 -1.42776900

H 4.56861900 -1.74326900 -2.02596600

H 6.15447400 -0.96706600 -2.08266800

C 4.65852100 0.12084100 -0.95519300

H 4.24795300 0.69361800 -1.78568400

H 5.40941500 0.74160200 -0.44400500

C 2.26793700 0.10781000 -0.41012900

O 2.01857300 0.71168400 -1.46780500

O -0.57881900 1.16554300 2.47664800

C -0.40652800 2.25368800 0.42811500

C -0.39737400 2.16975600 -0.97265500

C -0.49659100 3.49304300 1.08024600

C -0.47313400 3.34675300 -1.71267300

H -0.31186300 1.21487000 -1.46850400

C -0.56422800 4.65443400 0.32166000

H -0.51522300 3.51524000 2.16224500

C -0.55497100 4.58635100 -1.07622900

H -0.45996000 3.28870200 -2.79615400

H -0.62944300 5.61650200 0.82049000

H -0.61267500 5.49695600 -1.66482100

N -0.41646100 1.07465000 1.22839900

A-int2

0 1

C -0.60431000 1.26012900 -0.68497800

C -0.90230200 -0.17846200 -0.60784500

C -1.22690300 2.10888000 -1.51229200

O 0.15221900 -0.97945600 -0.72483600

H -2.01904000 1.77504900 -2.17375700

H -0.98292600 3.16648800 -1.51580600

Si 0.43719300 -2.49831900 0.07298100

C 0.00429200 -3.95383400 -1.02578400

H 0.31248800 -4.89247300 -0.55162000

H 0.52192700 -3.88144800 -1.98779500

H -1.07009200 -3.97943200 -1.20797200

C 2.34202900 -2.37752300 0.24546900

C -0.36028200 -2.45313000 1.76941500

H 0.16933800 -3.09165900 2.48462700

H -1.40547100 -2.76110900 1.70442000

H -0.32751900 -1.42510500 2.14801800

C 2.90532000 -3.69504600 0.81681000

H 3.99413400 -3.62196300 0.94561600

H 2.71324900 -4.54599800 0.15375600

H 2.47969600 -3.93214400 1.79901600

C 2.69676700 -1.21946800 1.19975200

H 2.32195100 -1.39501700 2.21352500

H 2.27268500 -0.27222700 0.85674000

H 3.78761200 -1.09776600 1.26792100

C 2.98160600 -2.10899900 -1.13209300

H 4.07065700 -1.99391500 -1.03527000

H 2.58535800 -1.19501500 -1.58578000

H 2.80382800 -2.93171600 -1.83394100

N 1.45286000 2.46885700 0.00500200

C 2.27622200 3.10085100 1.04541600

H 2.28226700 4.18349800 0.85112300

H 1.78877800 2.92341900 2.00331700

C 3.71353800 2.56832700 1.03432700

H 3.70598600 1.51829200 1.34625300

H 4.30523700 3.12313900 1.77137000

C 4.32942500 2.69099600 -0.36580300

H 5.33441500 2.25573200 -0.38415200

H 4.43893500 3.75429100 -0.62383200

C 3.43129300 2.00670000 -1.40433500

H 3.40015200 0.92839400 -1.21095600

H 3.82739100 2.14517200 -2.41694700

C 1.99796100 2.55615100 -1.35405300

H 1.35263700 2.00301500 -2.03385200

H 1.99361200 3.61054300 -1.66797800

C 0.37085300 1.72689400 0.37330600

O 0.10070700 1.45777700 1.54798200

O -2.28585200 -1.99001600 -0.51265800

C -3.27504400 0.08082300 -0.10871800

C -3.24181500 1.00485800 0.93597700

C -4.45193700 -0.19871000 -0.80158900

C -4.41172000 1.69476100 1.25493100

H -2.31672000 1.17692000 1.47714300

C -5.61125600 0.50115400 -0.47494700

H -4.43912600 -0.96615100 -1.56671100

C -5.59238700 1.45161100 0.54977200

H -4.40026800 2.41607300 2.06645400

H -6.53145300 0.29864500 -1.01454900

H -6.49933500 1.99089400 0.80644900

N -2.10368600 -0.70002500 -0.44884600

A-int3

0 1

C 1.01707300 -0.03012400 -0.08383100

C -0.37738900 0.25481600 -0.17600800

C 2.27136700 0.72947600 -0.42090700

O -1.22287700 -0.73577700 -0.25142800

H 2.34645300 1.11191600 -1.44536900

H 2.57417500 1.53209400 0.25883600

Si -3.01154300 -0.41458300 -0.08535000

C -4.08393900 -0.10320200 -1.61786500

H -4.95656900 -0.75968000 -1.66591000

H -3.48084600 -0.28470400 -2.51428100

H -4.41283300 0.93821200 -1.65614000

C -3.27844000 -2.33052100 0.24086200

C -3.47475300 0.39047300 1.56786500

H -4.05427500 -0.26173400 2.22658000

H -4.03001500 1.31619800 1.39630100

H -2.55622800 0.66763500 2.09920000

C -4.76059400 -2.62189900 0.55639800

H -4.90913300 -3.69529800 0.74571000

H -5.42824900 -2.35000200 -0.26851200

H -5.10797200 -2.09010800 1.45004900

C -2.43134400 -2.81902000 1.43567000

H -2.66766700 -2.27071900 2.35595300

H -1.36010400 -2.71073900 1.24345500

H -2.62539300 -3.88327400 1.63828600

C -2.87301400 -3.14318600 -1.00657600

H -3.02569600 -4.22015400 -0.83976000

H -1.81635400 -2.99431900 -1.25135800

H -3.46635900 -2.86558500 -1.88592800

N 3.13273900 -0.49450600 -0.22961400

C 4.06864600 -0.42703100 0.92151300

H 4.85495700 0.30562100 0.68724000

H 3.49887600 -0.05567100 1.77796700

C 4.67098300 -1.80399300 1.20787700

H 3.86954200 -2.47789200 1.52748300

H 5.37709700 -1.70963200 2.03951900

C 5.36006000 -2.37080400 -0.04040400

H 5.73900700 -3.37778300 0.16032300

H 6.22886900 -1.74791500 -0.29694800

C 4.37896800 -2.39746700 -1.21976700

H 3.56595000 -3.09989900 -1.01042800

H 4.87536100 -2.72810200 -2.13804800

C 3.77814000 -1.01138500 -1.46379100

H 3.00847400 -1.04286000 -2.23994900

H 4.55268900 -0.29510800 -1.77486000

C 1.57167300 -1.27870000 0.13769700

O 1.48443600 -2.43576600 0.41630900

O -2.24087800 1.46387000 -0.59747400

C -0.31310700 2.72398700 -0.04582800

C 0.55704600 2.92621600 1.03194600

C -0.65483700 3.78589800 -0.89145300

C 1.13437300 4.18216100 1.22258400

H 0.75170600 2.11118200 1.72081600

C -0.07849100 5.03559400 -0.68516700

H -1.37459800 3.60796400 -1.68136400

C 0.82470300 5.23768700 0.36367100

H 1.80630300 4.33972700 2.06115700

H -0.33763000 5.85859200 -1.34446600

H 1.26819500 6.21607100 0.52057700

N -0.92621500 1.46702800 -0.26287000

A-ts2

0 1

C -1.01030700 0.46506800 0.41927200

C 0.33906600 0.38392200 0.51023300

C -0.38552300 1.04487900 1.65360200

O 1.39800400 0.65075400 -0.26130600

H -0.33905600 2.13329700 1.76123200

H -0.41964200 0.50719000 2.61061100

Si 3.02574900 0.53762700 0.27643000

C 3.63644800 -1.20117300 -0.09676500

H 4.72920600 -1.25569100 -0.04338600

H 3.33050100 -1.51012800 -1.10081900

H 3.22570100 -1.92253200 0.61491200

C 3.90643100 1.83755800 -0.79628700

C 3.09549100 0.91246100 2.11316000

H 4.11429200 0.78321600 2.49399200

H 2.43618800 0.22879800 2.65704100

H 2.77984900 1.93740000 2.33073000

C 5.40058100 1.90119600 -0.41623200

H 5.91877000 2.64471000 -1.03658300

H 5.90758700 0.94159200 -0.57123400

H 5.54611300 2.19328300 0.63037700

C 3.25956700 3.21953600 -0.56848000

H 3.34995700 3.54746200 0.47352000

H 2.19650000 3.21080400 -0.82950500

H 3.75186100 3.97746200 -1.19271300

C 3.76905100 1.45453300 -2.28516400

H 4.25467200 2.20955900 -2.91830600

H 2.71890500 1.39074900 -2.58858000

H 4.24337200 0.49124100 -2.50443500

N -3.38843200 0.56877900 -0.08612300

C -3.74521300 1.17052100 1.19478700

H -4.51163400 0.53918300 1.67021200

H -2.87875100 1.17875800 1.85240500

C -4.30727800 2.58601000 0.99383800

H -3.50568900 3.22781700 0.60594300

H -4.61921200 2.99886700 1.96057900

C -5.48271100 2.56540700 0.00435600

H -5.84024400 3.58308500 -0.18877500

H -6.32128800 2.01733500 0.45795700

C -5.08132500 1.87506800 -1.30837700

H -4.32652400 2.47843400 -1.82845200

H -5.94453500 1.78584400 -1.97834700

C -4.49122500 0.48223700 -1.04124100

H -4.10722000 0.02162200 -1.95077500

H -5.26716800 -0.17559400 -0.62133700

C -2.11206400 0.24734700 -0.49633100

O -1.88587700 -0.24165000 -1.61425700

O 0.59461700 -1.47395600 2.41902800

C 0.05506400 -2.44184000 0.41373300

C 0.06127700 -2.33033900 -0.98411700

C -0.34102600 -3.62995300 1.04487300

C -0.34686200 -3.41972300 -1.74839100

H 0.34316600 -1.39802400 -1.45473400

C -0.74164500 -4.70923900 0.26706600

H -0.31804500 -3.67866700 2.12709300

C -0.74509800 -4.60675000 -1.12962500

H -0.36318100 -3.33466800 -2.82994400

H -1.05008800 -5.63411600 0.74528500

H -1.05931400 -5.45329500 -1.73298800

N 0.59047900 -1.35639400 1.18581100

A-ts3

0 1

C 1.33388100 -0.54572000 0.31548200

C -0.04154600 -0.08174400 0.41026100

C 0.89209700 -1.12917000 1.51905100

O -1.03510100 -0.59120700 -0.36001500

H 0.21017000 -1.97939000 1.50261000

H 1.29687500 -0.86121800 2.49705700

Si -2.65832500 -0.91338500 0.07513000

C -3.76182700 0.25482500 -0.89362000

H -4.81239200 -0.04587700 -0.81471400

H -3.48784000 0.25049200 -1.95271500

H -3.67006200 1.28157500 -0.52993800

C -2.91999300 -2.72342100 -0.45663500

C -2.84957000 -0.68676200 1.93365900

H -3.86720100 -0.94728600 2.24348700

H -2.66113000 0.34820400 2.23234000

H -2.15572100 -1.32287800 2.49159400

C -4.31372200 -3.20476600 -0.00194200

H -4.48275000 -4.24036100 -0.32574700

H -5.11989000 -2.59712500 -0.42962400

H -4.41866100 -3.18390900 1.08882700

C -1.83554500 -3.61503900 0.18206600

H -1.86459100 -3.57649800 1.27777400

H -0.83439800 -3.31731000 -0.14589700

H -1.98273600 -4.66374800 -0.10907300

C -2.81401600 -2.83015000 -1.99277700

H -2.92763800 -3.87577000 -2.30973500

H -1.84237900 -2.47715300 -2.35441600

H -3.59443600 -2.24978500 -2.49720900

N 3.70206100 -0.02467200 0.53077000

C 4.08664700 -1.43128300 0.67920800

H 4.76113100 -1.51042500 1.54321300

H 3.20786900 -2.03638500 0.89521500

C 4.79748400 -1.94350800 -0.58558100

H 4.07118000 -1.94876500 -1.40858900

H 5.12240900 -2.97944100 -0.42994600

C 5.98914500 -1.04596500 -0.95061700

H 6.44428900 -1.37861200 -1.89047500

H 6.76176700 -1.14344800 -0.17404900

C 5.55578800 0.42470700 -1.04691200

H 4.86701100 0.56041100 -1.88978600

H 6.42141100 1.07382500 -1.22443600

C 4.83402300 0.85919200 0.23620900

H 4.45143000 1.87561600 0.15368900

H 5.52524600 0.80982800 1.08906500

C 2.44696900 0.38347300 0.10627200

O 2.26850900 1.48450600 -0.43031400

O 0.24617900 1.53238000 2.10233700

C -1.01449200 2.14983500 0.26908700

C -0.84394000 2.29464600 -1.10967600

C -1.87512300 2.97743700 0.99207800

C -1.59028400 3.26245300 -1.77677900

H -0.10947500 1.68486600 -1.62080700

C -2.62392600 3.93338500 0.30942000

H -1.93769200 2.85797900 2.06738200

C -2.48886900 4.07163800 -1.07506200

H -1.46097900 3.39374400 -2.84651900

H -3.30801900 4.57349600 0.85786200

H -3.07191200 4.81943800 -1.60401000

N -0.26350500 1.15945300 0.99645000

A-ts4

0 1

C 0.72238700 0.79213300 0.04543000

C -0.61698000 0.37616300 -0.23637100

C 1.36368200 2.00154800 -0.36751000

O -0.82178500 -0.92462800 -0.39897700

H 1.06433500 2.49404100 -1.29805800

H 1.83219400 2.68144900 0.34012100

Si -2.22330900 -1.90536500 -0.16754100

C -3.10701900 -2.18366000 -1.79842500

H -3.97157100 -2.84280800 -1.66019000

H -2.44159100 -2.65569500 -2.52863900

H -3.44635400 -1.22643200 -2.19663500

C -1.37620300 -3.52050600 0.40941700

C -3.30246100 -1.22050500 1.20836800

H -3.92935300 -2.00483700 1.64691000

H -3.93079500 -0.41672200 0.82125000

H -2.67843700 -0.81155700 2.01039600

C -2.42239600 -4.65198600 0.48360400

H -1.95522000 -5.58015500 0.84101100

H -2.86653000 -4.86690700 -0.49517200

H -3.23885200 -4.41546000 1.17694600

C -0.74527900 -3.31108400 1.80252200

H -1.49720600 -3.05016300 2.55644200

H 0.01609700 -2.52484700 1.78287100

H -0.25655300 -4.23636600 2.13965200

C -0.26072600 -3.92118300 -0.57869300

H 0.21052300 -4.86117000 -0.25850700

H 0.51832300 -3.15441800 -0.62237300

H -0.64560900 -4.08001500 -1.59313200

N 2.94339500 0.74900400 -0.13452600

C 4.09636100 0.99329300 0.76103600

H 4.66438300 1.82475400 0.32427900

H 3.70230800 1.32150500 1.72725000

C 4.99624500 -0.24446000 0.91934500

H 4.42977100 -1.00916900 1.45757200

H 5.86648400 0.02786300 1.52765500

C 5.42960100 -0.78647900 -0.44967000

H 6.00395600 -1.71036800 -0.32481000

H 6.09619700 -0.06253900 -0.94067000

C 4.20261900 -1.03414600 -1.33913700

H 3.57999900 -1.82241300 -0.90286600

H 4.50079400 -1.36112300 -2.34159500

C 3.36393300 0.24384500 -1.46554600

H 2.45702100 0.07376900 -2.05165700

H 3.94744400 1.03878900 -1.94800100

C 1.79420800 -0.04310000 0.46434400

O 1.96060300 -1.09356700 1.06067700

O -2.76083200 0.79746500 -0.96440000

C -1.59815900 2.61659900 -0.08680700

C -1.12022600 3.04827400 1.15477400

C -2.16441600 3.52229600 -0.98590100

C -1.15725800 4.40692000 1.46648000

H -0.72668200 2.32504100 1.85987500

C -2.19233300 4.87743000 -0.66550800

H -2.58583800 3.13861800 -1.90736500

C -1.68287300 5.32658700 0.55612400

H -0.78987400 4.74321600 2.43156900

H -2.62262200 5.58475800 -1.36827300

H -1.71266500 6.38307300 0.80461400

N -1.63309500 1.21804800 -0.44126800

A-ts5

0 1

C -0.91555100 -1.10590000 -0.30429800

C 0.47232600 -0.76138000 -0.34595200

C -1.96471100 -0.23836000 -0.99000000

O 0.85134500 0.44453800 -0.81612400

H -1.73911000 0.82713500 -0.83784200

H -1.92191500 -0.40820100 -2.07292500

Si 1.55923800 1.76211400 0.04623700

C 2.92090900 2.39666100 -1.07768300

H 3.36331800 3.31148500 -0.67043200

H 2.53475600 2.62576000 -2.07613900

H 3.71448200 1.65280200 -1.17915600

C 0.22782000 3.10358100 0.31482000

C 2.19763800 1.14138700 1.70009000

H 2.38618900 1.98795200 2.36899300

H 3.12642100 0.57612200 1.59380300

H 1.46334100 0.49075400 2.18570700

C 0.91077600 4.37672000 0.86588500

H 0.15496000 5.14628400 1.07163700

H 1.62359200 4.80500800 0.15338100

H 1.44259700 4.19010900 1.80606200

C -0.80912400 2.61947700 1.35032000

H -0.35063800 2.45488000 2.33146800

H -1.29358300 1.68608700 1.05393200

H -1.59953500 3.37096500 1.48029800

C -0.46600100 3.45529300 -1.01665600

H -1.23123100 4.22648100 -0.85632300

H -0.95797700 2.58981200 -1.46870600

H 0.24686000 3.84985600 -1.74939800

N -3.30109900 -0.60454300 -0.52351400

C -4.35301200 -0.31539700 -1.50023600

H -4.46557400 0.77715600 -1.65820200

H -4.06187700 -0.75153200 -2.46197700

C -5.69015800 -0.90552900 -1.04178600

H -5.59298400 -1.99750200 -1.01019600

H -6.46682000 -0.66426700 -1.77671500

C -6.07322600 -0.38421700 0.34958300

H -6.99174500 -0.86587300 0.70313400

H -6.28233600 0.69334600 0.28731600

C -4.92125900 -0.61715900 1.33559200

H -4.77499600 -1.69277500 1.49117100

H -5.14465900 -0.16948900 2.31077400

C -3.62035000 -0.02675200 0.78787900

H -2.79105200 -0.23585900 1.47075400

H -3.71339600 1.07612400 0.71707000

C -1.38592300 -2.27120200 0.21508300

O -1.78906100 -3.27020600 0.65946300

O 0.84246000 -2.76845500 0.59588700

C 2.76908900 -1.60952200 -0.08450300

C 3.37920800 -1.06051000 -1.21599500

C 3.53551800 -2.19181300 0.92933600

C 4.77169700 -1.05344000 -1.30339800

H 2.77198400 -0.64808300 -2.01217500

C 4.92418200 -2.17504200 0.83125400

H 3.01990600 -2.65245000 1.76301500

C 5.54818100 -1.60048800 -0.28008600

H 5.24781100 -0.63057400 -2.18313300

H 5.52167900 -2.61810000 1.62227600

H 6.63124000 -1.59316200 -0.35482100

N 1.34607500 -1.67907200 0.04933300

B-int1

0 1

C -2.22502600 0.25124900 -1.68917800

C -3.12360000 -0.31316300 -0.74279400

C -3.58655700 0.80154200 -1.67743200

O -3.06123000 -0.30574200 0.63118500

H -3.74845700 1.78949200 -1.24707000

H -4.32376000 0.52784800 -2.44075100

Si -4.06971400 0.54035700 1.70459900

C -4.34566700 -0.62546300 3.15111000

H -4.94320900 -0.14517700 3.93379000

H -3.39940100 -0.94102300 3.59850100

H -4.88041300 -1.52280100 2.82287000

C -3.12357800 2.10593500 2.24921500

C -5.70920500 0.93913000 0.86378900

H -6.50419300 1.03609700 1.61090800

H -5.98642900 0.13474500 0.17389600

H -5.67632300 1.86991100 0.29055900

C -4.01367900 2.93212200 3.20184600

H -3.48049700 3.83236000 3.53631800

H -4.28961700 2.36777700 4.10047000

H -4.93897700 3.26301700 2.71547300

C -2.74114400 2.96812600 1.02866900

H -3.62098900 3.29833600 0.46339300

H -2.06684500 2.42953000 0.35362600

H -2.21808800 3.87518300 1.35942100

C -1.82673100 1.69070000 2.97496900

H -1.26879700 2.57997500 3.29867800

H -1.17851900 1.10645500 2.31624600

H -2.03188700 1.09766700 3.87296700

N -0.57698700 1.05701200 -3.24928600

C -1.42264100 2.15265600 -3.72463000

H -1.40015700 2.14052000 -4.82347400

H -2.45275700 1.99332100 -3.41899300

C -0.88361900 3.49794600 -3.21304100

H -0.94936800 3.50046400 -2.11719300

H -1.51554900 4.31287400 -3.58607900

C 0.57649600 3.68815700 -3.65228500

H 0.98490100 4.61429200 -3.23134600

H 0.60669800 3.79925600 -4.74604000

C 1.43262800 2.48361200 -3.23351300

H 1.51492600 2.44097000 -2.14404400

H 2.44761800 2.56714800 -3.63907000

C 0.81149700 1.16016600 -3.70834200

H 1.37177700 0.30851600 -3.33241400

H 0.80010200 1.12121400 -4.80724000

C -0.91104000 0.20989400 -2.22596100

O -0.06721500 -0.63804500 -1.79248900

O -4.70265400 -1.66836200 -1.85171200

C -2.72278900 -2.74946800 -1.32203200

C -1.55617100 -2.75564200 -0.54543200

C -3.08883300 -3.85543600 -2.10747200

C -0.73392700 -3.87860000 -0.58210000

H -1.29221500 -1.91367300 0.07587400

C -2.25667000 -4.96635600 -2.12684700

H -4.00441500 -3.81512600 -2.68428800

C -1.07613500 -4.97901600 -1.36962100

H 0.19053200 -3.86097300 -0.01608100

H -2.52197000 -5.82455300 -2.73675100

H -0.42593900 -5.84862100 -1.39957800

N -3.58571200 -1.61954000 -1.29195700

Rh 3.00874400 0.12756600 1.60239500

C 1.30644200 2.38531100 0.95629900

O 2.22634400 2.02609400 1.75486500

C 0.51771500 -0.98501200 2.52917800

O 0.11145800 -0.92115200 1.32244500

C 3.12648400 -2.52918900 0.46877200

O 2.14057200 -2.20493900 -0.27145100

C 3.96026800 0.73694100 -1.05355400

O 4.31407300 0.79184600 0.16570400

Rh 1.39943600 -0.28959200 -0.15143900

O 2.83857900 0.31959300 -1.49176700

O 1.64312300 -0.58354400 2.95931100

O 3.69625000 -1.78785200 1.33095200

O 0.74917400 1.65337400 0.07145700

C 0.83213900 3.81561300 1.06568100

H 0.35046100 3.95997600 2.03654900

H 1.69445800 4.48586600 1.02073300

H 0.12476700 4.05060000 0.27068300

C 4.94062300 1.24733000 -2.08335300

H 4.70373500 2.29435800 -2.30120400

H 5.95985800 1.19323000 -1.69901100

H 4.84364100 0.67809700 -3.00960400

C 3.68972900 -3.91872900 0.28090300

H 4.08886600 -4.29512200 1.22425100

H 2.92593200 -4.58985500 -0.11511200

H 4.51105300 -3.86709200 -0.44177500

C -0.42213200 -1.60385900 3.53573100

H -1.36900800 -1.86180100 3.06254600

H 0.04394700 -2.50249800 3.95073500

H -0.58575500 -0.90605200 4.36049200

B-int2

0 1

C -1.76478200 2.07118100 1.53905600

C -2.55839700 1.11149900 0.71307900

C -2.40524500 3.04060900 2.20361200

O -3.15465200 0.14951600 1.38068800

H -3.47254100 3.19398100 2.07852600

H -1.89235900 3.69014200 2.90531100

Si -4.54144100 -0.86419300 1.08516400

C -6.02115500 0.15688200 0.53585200

H -6.94987400 -0.40382000 0.69575100

H -5.93423600 0.42458800 -0.51782900

H -6.09004300 1.07697100 1.12795000

C -4.17002100 -2.42128000 0.02910900

C -4.80890600 -1.36165000 2.88784000

H -5.67603700 -2.01973300 3.00407900

H -4.97262500 -0.47041000 3.50286700

H -3.93255800 -1.88292600 3.28689400

C -4.71512600 -3.67137200 0.75874700

H -4.52074800 -4.56465900 0.14976300

H -5.79893000 -3.62269800 0.92175000

H -4.23677800 -3.82768800 1.73087900

C -2.64599400 -2.56215900 -0.13789800

H -2.12231200 -2.63213100 0.82109800

H -2.23176000 -1.70366700 -0.66268300

H -2.41425800 -3.46814800 -0.71802100

C -4.83910700 -2.34316200 -1.35938200

H -4.62999500 -3.26329200 -1.92301300

H -4.46563900 -1.49739800 -1.93766000

H -5.92933700 -2.25174500 -1.28166600

N 0.58734500 2.62741400 2.11175600

C 1.81154500 2.24461700 2.84572600

H 1.75626700 2.75425200 3.81987900

H 1.78828700 1.16898200 3.00888500

C 3.09547200 2.65513600 2.12209000

H 3.21194400 2.02055200 1.24244300

H 3.94201900 2.45810200 2.78985400

C 3.05422500 4.13215200 1.70988700

H 3.94068800 4.39095500 1.11945800

H 3.07350800 4.77209900 2.60398900

C 1.77815800 4.41655700 0.90904500

H 1.78560100 3.82621700 -0.01245200

H 1.70948400 5.47476300 0.63177700

C 0.51943300 4.03634500 1.70024900

H -0.36467700 4.18663700 1.08291200

H 0.43192500 4.67088800 2.59460300

C -0.34093000 1.68630800 1.82966100

O -0.09665200 0.46144300 1.87562500

O -3.57381200 0.44622900 -1.21534900

C -2.20280100 2.34173200 -1.34662700

C -3.09861300 3.06912700 -2.13341100

C -0.83812300 2.63043400 -1.34992000

C -2.62855300 4.13931300 -2.89009600

H -4.14101500 2.77267900 -2.14196500

C -0.38075400 3.70876500 -2.11272600

H -0.13691500 2.00795800 -0.81252800

C -1.26906500 4.46820900 -2.87507200

H -3.32246300 4.71548100 -3.49484600

H 0.67929100 3.94200500 -2.11771200

H -0.90408100 5.30182200 -3.46776500

N -2.75517400 1.25300800 -0.57734500

Rh 2.52162600 -1.84758800 -1.22207600

C 0.86693200 -3.42650500 0.53178500

O 1.69078700 -3.54056300 -0.43274200

C -0.06691000 -1.00995300 -2.19341100

O -0.24244900 -0.46358200 -1.05200000

C 2.87464800 1.01013300 -1.46478100

O 2.03238400 1.14153200 -0.51103200

C 3.85607600 -1.34444600 1.30169500

O 4.02237000 -1.92303700 0.18225900

Rh 1.23417000 -0.56980700 0.35715900

O 2.82177200 -0.69039100 1.66393800

O 0.95817100 -1.68158500 -2.54023700

O 3.29161100 -0.08489200 -1.94657400

O 0.50422900 -2.33565800 1.08135900

C 0.23649900 -4.69146100 1.05972400

H 0.82352200 -5.56215000 0.76595700

H 0.14058300 -4.63477400 2.14593100

H -0.76997000 -4.77708000 0.63829000

C 4.97223500 -1.45429800 2.31340800

H 4.69531100 -2.20422100 3.06166000

H 5.89961700 -1.76057800 1.82872800

H 5.10231000 -0.50040500 2.82946200

C 3.40126600 2.28381900 -2.08489000

H 4.29700100 2.07893400 -2.67145300

H 2.63249900 2.69610100 -2.74676600

H 3.61266500 3.02339900 -1.30919300

C -1.15825900 -0.81819100 -3.21525000

H -2.05962900 -0.41909800 -2.74609800

H -0.79891600 -0.11593500 -3.97539200

H -1.36322400 -1.76858900 -3.71363600

B-int3

0 1

C 1.60516800 -1.85496500 0.91037400

C 2.57070500 -0.92418100 0.36616600

C 1.49231700 -3.35726100 0.98680000

O 2.92649200 0.07119400 1.15906100

H 1.28805200 -3.89411400 0.05505900

H 2.25858800 -3.89465400 1.55685300

Si 4.21698100 1.19124800 1.36935000

C 5.89771100 0.41407100 1.06161400

H 6.69561200 1.08295700 1.40580500

H 6.02757300 0.19511900 0.00156600

H 5.98903800 -0.52280400 1.62318200

C 3.90102800 2.83288100 0.43992400

C 4.00852600 1.48651700 3.22219900

H 4.80083500 2.12657600 3.62392600

H 4.04081900 0.53207300 3.75844400

H 3.04458300 1.95728700 3.44227400

C 4.59520000 3.97292400 1.22296000

H 4.44594200 4.92594500 0.69745300

H 5.67843300 3.81885900 1.30616400

H 4.19174900 4.09078800 2.23383900

C 2.38090600 3.09715400 0.41228500

H 1.95799800 3.16463800 1.42132200

H 1.84015200 2.30259600 -0.10286300

H 2.17384400 4.04868100 -0.09970000

C 4.46045500 2.81855200 -0.99521900

H 4.24479000 3.77637700 -1.49033000

H 4.03501100 2.00845400 -1.58769100

H 5.54875400 2.68626800 -0.99733900

N 0.21027400 -3.19464700 1.79989900

C 0.31381200 -3.63489800 3.22558100

H 0.42573900 -4.72700300 3.22380400

H 1.22749000 -3.18896800 3.62787700

C -0.92400000 -3.20799400 4.01740100

H -0.96314300 -2.11476500 4.04907200

H -0.81305500 -3.56923700 5.04548000

C -2.20397100 -3.75610700 3.37124900

H -3.08267000 -3.39887900 3.91733200

H -2.20827100 -4.85375900 3.43887300

C -2.28153500 -3.32222500 1.90156400

H -2.40987800 -2.23920600 1.83199000

H -3.14009000 -3.78335100 1.40077400

C -1.02251200 -3.72841400 1.13499800

H -1.03648800 -3.31844500 0.12474700

H -0.91335700 -4.82003100 1.09280900

C 0.45258600 -1.59780100 1.59242400

O -0.33592800 -0.73290400 1.98433800

O 4.04340100 -0.23898300 -1.26548700

C 2.63388600 -2.02672800 -1.79158400

C 3.61246900 -2.71536500 -2.51135400

C 1.27414300 -2.22540400 -2.05201600

C 3.22571300 -3.65751300 -3.46210800

H 4.65374400 -2.48171800 -2.32227700

C 0.90123000 -3.17042300 -3.01000400

H 0.52813100 -1.64909300 -1.51967400

C 1.86992200 -3.89288100 -3.71073500

H 3.98379700 -4.20322800 -4.01618300

H -0.15318800 -3.32580500 -3.21818200

H 1.57190000 -4.62331400 -4.45719800

N 3.08834400 -1.03819400 -0.84361300

Rh -2.65490800 1.61338500 -1.32533500

C -1.44803700 3.25049700 0.72082300

O -2.13401600 3.33472000 -0.34891400

C 0.13887800 1.24774100 -1.96911700

O 0.25478900 0.65332600 -0.84243100

C -2.58393300 -1.25010500 -1.75864800

O -1.82664000 -1.33439700 -0.73206000

C -4.21466900 0.74819300 0.95672600

O -4.31131500 1.41430200 -0.12163700

Rh -1.41134600 0.39426400 0.33754300

O -3.16323300 0.17324900 1.39481400

O -0.93822800 1.73610600 -2.43788900

O -3.09171300 -0.18884400 -2.22709700

O -1.05041800 2.17379700 1.27473700

C -1.03361700 4.54215300 1.38180100

H -1.69237200 5.35615600 1.07726800

H -1.03411700 4.42455900 2.46711600

H -0.01067100 4.77452700 1.06827100

C -5.44962200 0.64941300 1.82132300

H -5.40835400 1.43916100 2.57903200

H -6.34845500 0.78994900 1.21966300

H -5.47319500 -0.31239300 2.33724600

C -2.91011300 -2.53532400 -2.48648800

H -3.99159300 -2.69694700 -2.46200000

H -2.61845500 -2.43989400 -3.53597800

H -2.40084800 -3.38647600 -2.03283200

C 1.38240900 1.40550800 -2.80647600

H 2.20172800 0.78931100 -2.43784200

H 1.15163700 1.16583700 -3.84707700

H 1.68974700 2.45554600 -2.76859500

B-ts2

0 1

C -2.15203200 0.58957800 1.59475200

C -2.92191400 0.36441000 0.49658000

C -3.53336800 0.05369500 1.83492800

O -2.81966400 -0.24546700 -0.68432400

H -3.66031300 -0.99156400 2.13128900

H -4.33629900 0.70956100 2.19129200

Si -3.95213600 -1.37628600 -1.29569500

C -4.17652300 -0.97810700 -3.11598800

H -4.93354000 -1.63682500 -3.55606000

H -3.25340000 -1.10131800 -3.68614500

H -4.51943600 0.05428000 -3.24147500

C -3.11352300 -3.06517400 -1.02207700

C -5.59081500 -1.17623800 -0.39390600

H -6.40445700 -1.60100100 -0.99161900

H -5.80057800 -0.11290500 -0.23400800

H -5.60214700 -1.66260800 0.58503200

C -4.01228100 -4.19394100 -1.56613000

H -3.53140400 -5.17038800 -1.41891100

H -4.20078100 -4.08471000 -2.64063900

H -4.98236900 -4.22848300 -1.05598000

C -2.85942000 -3.28173500 0.48453200

H -3.78759000 -3.26656300 1.06716300

H -2.18302100 -2.51960300 0.88638800

H -2.38899300 -4.26001300 0.65066500

C -1.75317000 -3.07516100 -1.75316000

H -1.23519800 -4.02831800 -1.58080200

H -1.10494800 -2.27010100 -1.39471400

H -1.86837000 -2.96626000 -2.83709500

N -0.53687100 0.74075900 3.38652100

C -1.39153400 0.02402700 4.33476100

H -1.35427500 0.56529900 5.29012800

H -2.42273400 0.03781100 3.99184400

C -0.87470600 -1.40885300 4.53341800

H -0.94660100 -1.93716400 3.57380100

H -1.51284400 -1.93585200 5.25285800

C 0.58681800 -1.38236300 5.00868700

H 0.98213500 -2.40186700 5.08602900

H 0.62318700 -0.95296400 6.02048900

C 1.45453400 -0.54100700 4.06021500

H 1.52880000 -1.03052200 3.08507500

H 2.47189400 -0.43451100 4.45440500

C 0.85569400 0.85715100 3.83580100

H 1.42233200 1.40816100 3.08903400

H 0.85482200 1.42717000 4.77548300

C -0.85079700 0.92834600 2.07139000

O -0.00800700 1.45364400 1.27979900

O -4.78057600 2.32123500 0.60316100

C -2.78318100 3.15852200 -0.12664700

C -1.56288300 2.86379000 -0.75027700

C -3.13115600 4.47442000 0.22028200

C -0.66588000 3.89917000 -1.00245500

H -1.30848300 1.84533300 -1.01188300

C -2.23322600 5.49761800 -0.05181600

H -4.08860300 4.65802000 0.69413400

C -0.99898200 5.21009700 -0.65584400

H 0.30354300 3.66023000 -1.42590800

H -2.48334300 6.52100500 0.21202900

H -0.29557400 6.01553200 -0.84777500

N -3.68543100 2.07434300 0.09712800

Rh 2.93125300 -0.92149500 -1.39097700

C 1.24474500 -2.53601400 0.32997300

O 2.13648700 -2.63370500 -0.56863400

C 0.41962800 -0.29704500 -2.66711800

O 0.06459400 0.25422000 -1.57482100

C 3.10550600 1.94612500 -1.70870500

O 2.16863500 2.05115100 -0.84978700

C 3.97535700 -0.21141200 1.20391300

O 4.29064300 -0.83875800 0.14443500

Rh 1.39597900 0.33535500 -0.01292100

O 2.87009200 0.38411300 1.42424600

O 1.50911600 -0.91765100 -2.87243000

O 3.63572000 0.86451100 -2.11641200

O 0.71588600 -1.45313700 0.75169100

C 0.78935800 -3.82236600 0.97841900

H 0.54423600 -4.55591400 0.20715100

H 1.61608400 -4.22802400 1.57023600

H -0.07220300 -3.64964500 1.62240800

C 4.98402400 -0.19066300 2.32843300

H 4.67276200 -0.91313800 3.09036200

H 5.97399100 -0.46315900 1.96188300

H 5.00193000 0.79728900 2.79353500

C 3.60524900 3.23464000 -2.32018000

H 4.60071700 3.09520700 -2.74302200

H 2.92133000 3.53062300 -3.12321900

H 3.60978000 4.02807800 -1.57025800

C -0.53282100 -0.18412800 -3.83257900

H -1.46052400 0.29385600 -3.52126900

H -0.05704100 0.40681500 -4.62116100

H -0.73148000 -1.17781100 -4.24175800

B-ts3

0 1

C -2.05702400 -1.49058500 1.25274200

C -2.91737400 -0.30993000 1.17710200

C -3.22188200 -1.90257300 1.93885000

O -3.62057000 0.08814700 0.09897600

H -4.13379200 -2.14799800 1.39548200

H -3.23789500 -2.12014800 3.00814800

Si -4.27246100 -0.58500600 -1.32253000

C -4.64802100 0.95912700 -2.32481100

H -5.38962800 0.75595100 -3.10399700

H -3.74990100 1.35058600 -2.81127800

H -5.04275600 1.74133900 -1.66931200

C -3.13150100 -1.77149100 -2.29306600

C -5.87489000 -1.44934100 -0.83331300

H -6.43742800 -1.74370100 -1.72640500

H -6.50876000 -0.77744200 -0.24561700

H -5.70401500 -2.35598800 -0.24447500

C -3.78154100 -1.95560700 -3.68703300

H -3.17165000 -2.64073900 -4.29059900

H -3.85193600 -1.01266900 -4.23992000

H -4.78727800 -2.38888900 -3.62560900

C -3.00046900 -3.15679000 -1.62760700

H -3.97165500 -3.64724500 -1.49364900

H -2.50965500 -3.08189400 -0.65381100

H -2.38920200 -3.81613300 -2.25969700

C -1.72889000 -1.16048300 -2.46625100

H -1.11823300 -1.80621400 -3.11279000

H -1.20707700 -1.04809600 -1.51489100

H -1.77072800 -0.17709600 -2.94488200

N -0.13899500 -2.58640300 2.23098500

C -0.67667200 -3.92720400 1.98266700

H -0.76713700 -4.44896700 2.94644600

H -1.67289100 -3.85593300 1.55259500

C 0.25382000 -4.71405100 1.04720800

H 0.27012400 -4.20498700 0.07616600

H -0.15732700 -5.71884700 0.89253300

C 1.67495300 -4.78127300 1.62229500

H 2.34463600 -5.30328800 0.92867900

H 1.66019400 -5.37155700 2.55024900

C 2.19594400 -3.36772800 1.91514700

H 2.32621800 -2.81560100 0.98181400

H 3.16921200 -3.40490500 2.41825000

C 1.21556900 -2.58304500 2.79679200

H 1.53672200 -1.55094700 2.91515500

H 1.15177900 -3.05173100 3.79027200

C -0.70722300 -1.43483500 1.77225200

O -0.08933800 -0.33261200 1.79972000

O -2.48363600 0.41892500 3.35838500

C -2.96487200 2.06368000 1.82482200

C -2.35434500 2.59231600 0.68735200

C -3.66581500 2.86604000 2.72590000

C -2.47978400 3.95761500 0.43926800

H -1.75909500 1.95477100 0.04648600

C -3.79870900 4.22590400 2.45282300

H -4.08868200 2.41728300 3.61724000

C -3.20909400 4.77325100 1.30964400

H -1.99566200 4.38514000 -0.43394300

H -4.35529000 4.85907700 3.13699600

H -3.30835400 5.83514100 1.10439800

N -2.81041700 0.66453900 2.16306700

Rh 2.98930300 0.96685600 -1.41778600

C 1.85761200 -1.68275900 -1.76507900

O 2.66108300 -0.86545600 -2.31059200

C 0.27522000 1.74209400 -2.01341300

O -0.07155800 1.15382200 -0.93688200

C 2.51546700 2.96097900 0.61900800

O 1.66611800 2.16422300 1.13447200

C 4.14485300 -0.34433600 0.87636100

O 4.46228400 0.14163800 -0.25419700

Rh 1.38623800 0.32524700 0.26983900

O 2.97024600 -0.39536600 1.36809500

O 1.44863100 1.79318700 -2.49377300

O 3.21426500 2.74694500 -0.42531600

O 1.18407600 -1.47938700 -0.69973500

C 1.69709200 -3.04255200 -2.40403400

H 2.08108500 -3.03625900 -3.42437800

H 2.25981300 -3.77455300 -1.81446300

H 0.64604600 -3.33824000 -2.38794100

C 5.24934100 -0.95256400 1.70809800

H 5.23236300 -2.03902200 1.57058000

H 6.22080100 -0.57175500 1.39146600

H 5.07726800 -0.74506200 2.76612800

C 2.72962900 4.27295000 1.33637800

H 3.15721400 5.01329000 0.65911800

H 1.78612100 4.62624200 1.75673100

H 3.42686300 4.10859700 2.16481500

C -0.80332100 2.45665700 -2.79693800

H -1.76340100 2.40362200 -2.28425700

H -0.50993700 3.50118700 -2.93421300

H -0.88734100 2.00887500 -3.79117100

B-ts4

0 1

C 1.76279400 -1.80912000 1.09625300

C 2.49329700 -0.79989600 0.32477800

C 2.10478200 -3.17140800 1.36619200

O 2.62792800 0.37179800 0.91759400

H 2.47740300 -3.82371200 0.56927400

H 2.35550500 -3.51606100 2.36497300

Si 3.91363000 1.40307200 1.37739800

C 5.55705700 0.54235000 1.09397900

H 6.39397300 1.19797900 1.36015000

H 5.63242400 0.26335000 0.04017600

H 5.63762200 -0.36358000 1.70571400

C 3.72354200 3.05653900 0.45163200

C 3.57419000 1.64390400 3.21432300

H 4.34009800 2.26147200 3.69512400

H 3.54993600 0.67479500 3.72427100

H 2.60119900 2.12259200 3.36645700

C 4.44973400 4.16275900 1.25037000

H 4.37142900 5.11981500 0.71701500

H 5.51940200 3.95081700 1.37350700

H 4.01720600 4.30730500 2.24629900

C 2.22090600 3.39920800 0.35879600

H 1.75959300 3.49415100 1.34829000

H 1.66517000 2.62792400 -0.17733200

H 2.08778400 4.35750900 -0.16445700

C 4.32891800 2.98202000 -0.96448300

H 4.16873800 3.93389400 -1.49091000

H 3.88684600 2.17435900 -1.54967800

H 5.41020400 2.80487100 -0.93003800

N 0.17873300 -2.98881200 2.14902200

C -0.14519700 -3.16516000 3.58273600

H 0.03379400 -4.22343900 3.81477000

H 0.56473800 -2.56564400 4.16009300

C -1.59853000 -2.79168100 3.91594600

H -1.72835900 -1.71904200 3.74256100

H -1.77625000 -2.99276600 4.97879600

C -2.56746900 -3.58300500 3.02895400

H -3.59968200 -3.27867300 3.23253900

H -2.49717200 -4.65468500 3.26721800

C -2.22843400 -3.34645600 1.55269200

H -2.40166700 -2.29939100 1.30025400

H -2.86691200 -3.95158000 0.89727600

C -0.76659300 -3.70264400 1.25040500

H -0.50485400 -3.44251600 0.22395400

H -0.59496600 -4.77613100 1.40313700

C 0.54286500 -1.60122100 1.75312700

O -0.19519600 -0.63188900 1.98472200

O 3.83409900 -0.17537800 -1.45734900

C 2.71321900 -2.21989700 -1.59246900

C 3.77554800 -2.98632900 -2.07292700

C 1.38611000 -2.56588400 -1.85703300

C 3.50321100 -4.15247700 -2.78676300

H 4.78835300 -2.64554300 -1.88974100

C 1.12743100 -3.73726700 -2.57171700

H 0.58208100 -1.92827800 -1.50720400

C 2.17992200 -4.53431100 -3.02979700

H 4.32271700 -4.76108800 -3.15725700

H 0.10009600 -4.02020700 -2.77938700

H 1.97102600 -5.44242100 -3.58770700

N 3.02175800 -1.01751400 -0.86142700

Rh -2.63518800 1.49510400 -1.41961400

C -1.52649800 3.26370700 0.57134200

O -2.24542900 3.26924500 -0.48048500

C 0.15865300 1.28153400 -2.11271700

O 0.32717200 0.70978200 -0.98277400

C -2.36318200 -1.35858700 -1.79904500

O -1.60479100 -1.36750800 -0.77060200

C -4.10477000 0.58861800 0.90293700

O -4.26484300 1.20276500 -0.19859600

Rh -1.28660300 0.40797800 0.25037500

O -3.00614700 0.11969200 1.34920900

O -0.95127500 1.71905200 -2.55914800

O -2.95117600 -0.34645000 -2.28440800

O -1.02037300 2.23595200 1.12862500

C -1.21474500 4.60046100 1.19835400

H -2.00340300 5.31928800 0.97174600

H -1.08400200 4.49041500 2.27612800

H -0.27365500 4.96730000 0.77577300

C -5.31966300 0.41930800 1.78481000

H -5.32051600 1.21715500 2.53504100

H -6.23361500 0.49226100 1.19424600

H -5.27177700 -0.53656300 2.31042200

C -2.55141900 -2.67176400 -2.52333000

H -3.57223300 -2.74589700 -2.90245600

H -1.87251000 -2.69762500 -3.38225200

H -2.32469100 -3.51215200 -1.86524400

C 1.37764500 1.48156000 -2.97412700

H 2.19475400 0.82900900 -2.66110800

H 1.11598400 1.32671900 -4.02311300

H 1.70484800 2.52095700 -2.86189900

B-ts5

0 1

C 1.67465600 0.95567900 -1.79917500

C 2.58467300 -0.06737300 -1.33375000

C 2.20373400 2.40851900 -2.01114300

O 3.68678100 0.25658600 -0.63035600

H 3.15742000 2.45376500 -1.48017500

H 2.42914100 2.50841800 -3.08007400

Si 3.79578600 0.20728100 1.09712500

C 4.17096100 -1.54079100 1.67422300

H 4.23428000 -1.55530000 2.76842100

H 5.11436400 -1.91538400 1.27018100

H 3.38901300 -2.24175500 1.37371100

C 5.26104500 1.36349600 1.46463700

C 2.15593200 0.76514300 1.81913500

H 2.10852500 0.49847500 2.88112700

H 1.32154600 0.27568800 1.30550700

H 2.01194100 1.84286200 1.73375100

C 5.59993900 1.27305700 2.96964500

H 6.43112700 1.94897000 3.21010300

H 5.90694700 0.26347600 3.26343600

H 4.75187300 1.56579100 3.60066100

C 4.92256500 2.82683300 1.11589800

H 4.07377500 3.20195300 1.69823900

H 4.68737500 2.95218900 0.05418900

H 5.78010200 3.47609500 1.33780200

C 6.48312500 0.91621100 0.63363500

H 7.34389900 1.56449100 0.84530700

H 6.27812800 0.97240000 -0.44046000

H 6.78416300 -0.11126100 0.86708200

N 1.39942100 3.54268200 -1.62159100

C 1.17928900 3.65082500 -0.17675800

H 0.53089500 2.83797800 0.18806700

H 2.15233600 3.55402600 0.31617200

C 0.55134600 5.00260700 0.17779100

H 1.26032300 5.80188900 -0.07206100

H 0.37494300 5.04817500 1.25888700

C -0.75312500 5.21317100 -0.60134400

H -1.18411400 6.19655900 -0.38132300

H -1.48804300 4.45768900 -0.28983300

C -0.48928800 5.05637000 -2.10442900

H 0.18409600 5.85194500 -2.44700800

H -1.42064700 5.13925700 -2.67596800

C 0.15935300 3.69822900 -2.38786800

H 0.39875400 3.60242000 -3.45362400

H -0.57565800 2.91101700 -2.14171300

C 0.41932700 0.61469300 -2.11094300

O -0.69030900 0.31328200 -2.38652800

O 1.15179700 -1.55320700 -2.23249000

C 3.13585500 -2.46036600 -1.38344200

C 4.52367100 -2.40550800 -1.53319800

C 2.49269900 -3.65123200 -1.03552500

C 5.27672000 -3.55260200 -1.28300300

H 5.00183500 -1.48061500 -1.83079600

C 3.25830800 -4.78738200 -0.78084700

H 1.41013600 -3.65547000 -0.98633700

C 4.65062800 -4.74036000 -0.89724000

H 6.35593500 -3.51787300 -1.39896300

H 2.76604600 -5.71347200 -0.49938300

H 5.24359000 -5.62880000 -0.70207200

N 2.29857300 -1.32372800 -1.64594400

Rh -3.34345300 -0.84771400 1.39519300

C -0.65632400 -1.88981000 1.53323200

O -1.76766600 -1.91713400 2.15216300

C -1.84205600 1.59371500 1.77616900

O -1.39322800 1.43892400 0.59105700

C -4.64543300 0.80582500 -0.58379000

O -3.55407700 0.78323700 -1.24214300

C -3.43415600 -2.69423700 -0.81550000

O -3.94023300 -2.49507600 0.33745000

Rh -1.95926900 -0.23719000 -0.46745000

O -2.59628400 -1.93916200 -1.40554000

O -2.65900300 0.82392500 2.37139300

O -4.85275800 0.24623700 0.54125300

O -0.41736000 -1.26930600 0.44538900

C 0.49819400 -2.63853300 2.15581200

H 0.13428700 -3.36339300 2.88445400

H 1.08623100 -3.13174700 1.37969600

H 1.15007700 -1.92142400 2.66452800

C -3.83801600 -3.95531900 -1.53971100

H -3.07694800 -4.72140700 -1.35697500

H -4.79701000 -4.31790400 -1.16760500

H -3.87978000 -3.77171200 -2.61474200

C -5.80515200 1.53711500 -1.21508600

H -6.51291300 1.85861000 -0.44997000

H -5.44345500 2.38793600 -1.79497300

H -6.31734500 0.85216200 -1.89928000

C -1.34729500 2.80197700 2.53496200

H -1.62371000 3.71000700 1.99271000

H -1.77073300 2.82318200 3.53887300

H -0.25552200 2.76998100 2.58745800

C-int1

0 1

C 1.82740500 -1.57447900 0.05979800

C 2.50956100 -0.36079600 -0.28460800

C 1.86868800 -1.23978400 -1.36098600

O 3.84555400 -0.08374200 -0.16627500

H 2.61924500 -1.76816900 -1.95361900

H 0.98678900 -0.90822500 -1.91097400

Si 4.62074800 1.18592200 -1.01221600

C 4.52264600 2.74851300 0.02855300

H 5.19591900 3.52099300 -0.35915000

H 4.79913700 2.54265600 1.06706600

H 3.50859600 3.15882200 0.03552400

C 6.40750000 0.55857100 -1.17855500

C 3.74483500 1.42233800 -2.65878900

H 4.16707400 2.27173600 -3.20644500

H 2.67986600 1.62056200 -2.49366700

H 3.82638000 0.53522700 -3.29477400

C 7.25592100 1.58167000 -1.96177500

H 8.28716800 1.21882400 -2.06791200

H 7.30478900 2.55100600 -1.45218900

H 6.86511100 1.75169200 -2.97201500

C 6.40752300 -0.79105300 -1.92620800

H 6.02210000 -0.69244300 -2.94774200

H 5.79815800 -1.53582800 -1.40372200

H 7.42976700 -1.18605400 -2.00080200

C 7.00860900 0.35769100 0.22912700

H 8.03330000 -0.03127400 0.15345200

H 6.42277300 -0.35606800 0.81774300

H 7.05708600 1.29815400 0.78977800

N 0.53371600 -3.44856600 0.86154800

C -0.27098100 -3.57856200 -0.34997900

H -1.33231400 -3.50847500 -0.06209500

H -0.07514300 -2.74349400 -1.01329100

C -0.01370200 -4.92839600 -1.03084700

H 1.02116500 -4.94612400 -1.39671000

H -0.67064400 -5.02660400 -1.90266800

C -0.23971200 -6.08463000 -0.04549200

H 0.00712600 -7.04431600 -0.51296200

H -1.30625100 -6.12548500 0.21827000

C 0.58759800 -5.87802000 1.23270600

H 1.65702000 -5.94938600 0.99696800

H 0.36403600 -6.65764500 1.97007300

C 0.31304900 -4.50046600 1.85305600

H 0.95866200 -4.29857300 2.70725600

H -0.72962300 -4.45179000 2.20085500

C 1.37591000 -2.40029600 1.15855700

O 1.80653600 -2.21873600 2.31153100

O 0.73828000 1.13344200 -0.76341800

C 1.67054500 1.30356500 1.38507300

C 2.39714500 0.70635000 2.42878000

C 1.10897200 2.58262000 1.53554500

C 2.53226000 1.39512900 3.63010700

H 2.80064500 -0.29053700 2.32182200

C 1.25976400 3.25198300 2.74420400

H 0.57622400 3.03154100 0.71050700

C 1.96753100 2.66328200 3.79646200

H 3.07935800 0.93049100 4.44444500

H 0.83160100 4.24336100 2.85854800

H 2.08391800 3.19200900 4.73796400

N 1.57845100 0.68372600 0.11576200

Rh -3.75378700 0.24148100 -0.08171900

C -2.40222900 -1.47047000 -1.97353600

O -3.53209700 -1.29745300 -1.42138200

C -1.92634500 -0.99435900 1.79343800

O -0.98062000 -0.42044700 1.15592600

C -2.84058600 2.51947700 1.42618500

O -1.70575700 2.35510400 0.86785300

C -3.25393300 2.09805000 -2.25036400

O -4.20072800 1.51076000 -1.63462300

Rh -1.42905600 0.79439300 -0.42902700

O -2.01033400 2.00649800 -1.98490600

O -3.15695600 -0.97369100 1.47167600

O -3.87357700 1.80472900 1.23969600

O -1.34344100 -0.79572400 -1.74827000

C -2.31169200 -2.57410700 -3.00238100

H -2.78132200 -3.48026500 -2.61252900

H -2.86860000 -2.27085200 -3.89447000

H -1.27438800 -2.76865000 -3.27595500

C -3.63262600 2.96070800 -3.43128600

H -3.54475500 2.36137700 -4.34390800

H -4.66433300 3.30181600 -3.33711000

H -2.94826300 3.80675000 -3.51494100

C -2.95372300 3.68736400 2.37626000

H -3.84257800 3.58886100 2.99988300

H -2.05290200 3.74744200 2.99143900

H -3.02587100 4.61148900 1.79332400

C -1.54969600 -1.72829200 3.05598600

H -0.47443600 -1.90428500 3.10086700

H -1.84693800 -1.10690300 3.90782200

H -2.10868800 -2.66425400 3.12284900

C-int2

0 1

C -1.86148500 -1.05693900 0.65215100

C -1.67916600 0.39586800 0.52954600

C -1.16246900 -1.85382300 1.48340400

O -2.61753900 1.08073800 -0.12242400

H -0.42011700 -1.46463800 2.16030300

H -1.31160600 -2.92812600 1.44977300

Si -3.78835000 2.23786300 0.37723000

C -3.32989900 3.93380500 -0.27417000

H -4.15125200 4.63380900 -0.08510800

H -3.14915200 3.90958900 -1.35265100

H -2.43097000 4.32295300 0.20838500

C -5.39370800 1.60727100 -0.42336800

C -3.80770100 2.20046300 2.25629900

H -4.60365100 2.84062900 2.65191300

H -2.85600100 2.56428600 2.65689700

H -3.97073200 1.18682700 2.63606900

C -6.48962100 2.68991500 -0.33091800

H -7.42812100 2.31202400 -0.75761400

H -6.22226300 3.59523900 -0.88595100

H -6.70020500 2.97887700 0.70653300

C -5.87052000 0.33733400 0.30458700

H -6.10992500 0.52848900 1.35732200

H -5.11559800 -0.45067800 0.26152100

H -6.77930900 -0.05740200 -0.17077200

C -5.11668800 1.26498200 -1.90451200

H -6.03632100 0.90124700 -2.38312000

H -4.35103900 0.48886600 -2.00269400

H -4.78459300 2.14408600 -2.46954500

N -3.69223200 -2.59063800 -0.06205600

C -4.03201800 -3.01236500 1.30258600

H -3.56140700 -3.98788500 1.50449900

H -3.61838000 -2.29641700 2.01100700

C -5.54958900 -3.13457600 1.50883500

H -5.99554900 -2.13452100 1.50561200

H -5.72576500 -3.56126200 2.50305500

C -6.20869100 -3.99100500 0.42149300

H -7.29552800 -4.01024100 0.55769500

H -5.85863400 -5.02944500 0.51005100

C -5.84004000 -3.45001000 -0.96513700

H -6.26064600 -2.44569500 -1.09890600

H -6.25545200 -4.08479500 -1.75595400

C -4.32047900 -3.38093200 -1.13157800

H -4.03781500 -2.92705200 -2.07999100

H -3.89983000 -4.39843200 -1.10034500

C -2.73856400 -1.66809600 -0.42259100

O -2.54152500 -1.36116500 -1.59916500

O 0.31094000 0.46686500 1.73781200

C -0.33382900 2.40767500 0.58992200

C -0.23907500 2.73996200 -0.76147700

C -0.04244000 3.32703700 1.59349700

C 0.12663500 4.03797200 -1.10856800

H -0.42453000 1.97777600 -1.50741900

C 0.31393700 4.62697800 1.23416400

H -0.07295800 3.00940000 2.62974800

C 0.39696900 4.98332900 -0.11476700

H 0.20776100 4.31051100 -2.15663000

H 0.53731700 5.35661300 2.00642400

H 0.68133600 5.99435000 -0.39118700

N -0.61730700 1.04347800 0.97372100

Rh 3.85397600 -0.88316600 -0.92332100

C 1.29699100 -0.60504600 -2.26539200

O 2.49939300 -0.95326600 -2.47933500

C 3.52757200 1.98159400 -0.74273900

O 2.58107000 1.76899900 0.08263200

C 4.57429900 -0.46578000 1.85223400

O 3.35730400 -0.16644700 2.08193100

C 2.37767600 -3.04375700 0.30256800

O 3.37708700 -2.82632900 -0.45027000

Rh 2.03956800 -0.16379200 0.50443000

O 1.63969700 -2.16203500 0.85284600

O 4.22863600 1.09712000 -1.32792500

O 5.07703200 -0.77890100 0.72563800

O 0.82291800 -0.20402200 -1.14867400

C 0.32646800 -0.63088400 -3.42148800

H 0.67749600 -1.31285300 -4.19720800

H -0.66595900 -0.90954500 -3.06060600

H 0.26618900 0.37679500 -3.84917300

C 1.99892100 -4.48692600 0.54960900

H 1.18040900 -4.75402900 -0.12777400

H 2.84621900 -5.14397700 0.35043900

H 1.64584200 -4.61278400 1.57531800

C 5.50435400 -0.48437700 3.04399000

H 6.52677300 -0.27065400 2.72860900

H 5.17076800 0.23225600 3.79615000

H 5.48279500 -1.48532700 3.48842500

C 3.80790100 3.42734300 -1.08082900

H 3.59819700 4.06090200 -0.21756600

H 4.83812300 3.54840200 -1.41808700

H 3.13369400 3.73041800 -1.88888200

C-int3

0 1

C 3.50119300 -0.51838100 -0.24185200

C 2.27198000 0.15497500 -0.52499700

C 3.94756600 -1.95444300 -0.15258400

O 2.36461300 1.45228000 -0.73203500

H 3.60165000 -2.53244500 0.70910600

H 3.82439100 -2.57170200 -1.04796900

Si 1.41613700 2.82324700 -0.25251600

C 0.71895100 2.42436500 1.44104400

H 0.10308100 3.25372700 1.80422600

H 1.53491800 2.26831800 2.15444800

H 0.09101800 1.53382900 1.41518200

C 2.77402900 4.16672800 -0.12218500

C 0.19976300 3.28639100 -1.59713600

H -0.37128200 4.16998600 -1.29155600

H -0.49650600 2.47099000 -1.78539300

H 0.73215000 3.53054500 -2.52271300

C 2.09425300 5.54447500 0.03824900

H 2.85278600 6.33128500 0.15128900

H 1.45210100 5.58589900 0.92722700

H 1.48116600 5.80541200 -0.83130000

C 3.63924400 4.16732400 -1.40057100

H 3.04174700 4.33781100 -2.30369900

H 4.17247700 3.21900700 -1.51509500

H 4.38858400 4.96978800 -1.34969300

C 3.69288400 3.91416100 1.09114500

H 4.48284100 4.67758800 1.12937400

H 4.18652200 2.93976300 1.02760700

H 3.14569400 3.96944300 2.03892500

N 5.38864800 -1.52639000 0.02704900

C 6.29084800 -1.87893500 -1.10403600

H 6.41218700 -2.97123200 -1.11194400

H 5.77974700 -1.58197600 -2.02397300

C 7.64279700 -1.17684100 -0.96068600

H 7.48832100 -0.09684000 -1.04653000

H 8.28494400 -1.48914400 -1.79093100

C 8.28937400 -1.50473900 0.39196500

H 9.22974200 -0.95634300 0.50545200

H 8.53633000 -2.57516700 0.43361000

C 7.32785800 -1.15282300 1.53513700

H 7.15856900 -0.07175700 1.55795800

H 7.74319200 -1.44628600 2.50494700

C 5.98092000 -1.85580400 1.35262200

H 5.25892600 -1.54228700 2.11181700

H 6.09320700 -2.94757600 1.41260600

C 4.74434000 0.07378000 -0.08311700

O 5.43377300 1.05497000 -0.01149400

O 0.04569300 0.19716400 -1.17207700

C 0.98217500 -1.89306200 -0.52038200

C 0.86496800 -2.67990600 -1.66637100

C 0.92993500 -2.45573200 0.75356600

C 0.74259900 -4.06072000 -1.53244400

H 0.85912800 -2.19829000 -2.63821300

C 0.80318200 -3.84041500 0.87976900

H 0.95661400 -1.80356100 1.61796500

C 0.71850900 -4.64296800 -0.26027900

H 0.65906700 -4.68349600 -2.41808200

H 0.75854200 -4.28979800 1.86758400

H 0.62181500 -5.72025100 -0.15908200

N 1.10750700 -0.46934300 -0.66984700

Rh -4.15982300 -0.23497700 0.64650500

C -3.06911800 -2.72151700 -0.33437500

O -4.10222300 -2.24599000 0.23297100

C -2.03365800 -0.61728000 2.58159200

O -1.19710700 -0.43324700 1.63389800

C -3.06379300 2.43396900 0.69853600

O -2.01281000 1.95701300 0.16037100

C -4.03953600 0.32844100 -2.18910800

O -4.86991900 0.13475300 -1.24496800

Rh -1.91613900 -0.05692400 -0.26605300

O -2.76863300 0.32048900 -2.09424600

O -3.29703800 -0.59143700 2.48608800

O -4.10799500 1.78509900 1.01739400

O -2.01841600 -2.07739200 -0.65419600

C -3.08818500 -4.19022900 -0.69267900

H -3.84598500 -4.71564300 -0.11031000

H -3.32829400 -4.28825200 -1.75680300

H -2.09895200 -4.62177500 -0.53048400

C -4.60716300 0.56222200 -3.57078200

H -4.55632400 -0.37542900 -4.13442900

H -5.64849200 0.87992700 -3.50681600

H -4.00649800 1.30439300 -4.10043900

C -3.04026600 3.91550700 1.00076500

H -2.65488500 4.46068700 0.13575600

H -4.03631400 4.27098500 1.26535300

H -2.35756200 4.09568000 1.83769800

C -1.43814100 -0.90886200 3.94119900

H -0.94604700 -1.88670900 3.91163100

H -0.67670100 -0.16041500 4.17651400

H -2.21287300 -0.91620800 4.70794500

C-ts2

0 1

C -1.87143600 1.44800200 0.07170600

C -2.70275400 0.45305500 -0.32240200

C -1.94116700 1.16078500 -1.40107600

O -3.94976900 0.04980000 -0.09540300

H -2.47782800 1.87547400 -2.03479100

H -1.12401900 0.63836100 -1.90392800

Si -4.73774000 -1.20964900 -0.96369000

C -4.64083800 -2.76446500 0.08565100

H -5.34419900 -3.52497600 -0.27087800

H -4.88158500 -2.53873700 1.12891300

H -3.63498600 -3.19304000 0.06506100

C -6.51851100 -0.55619900 -1.10161800

C -3.89796400 -1.43034700 -2.62879100

H -4.36402000 -2.25047000 -3.18582700

H -2.83745700 -1.66613200 -2.49693200

H -3.96667300 -0.52525600 -3.23984300

C -7.39185600 -1.56339100 -1.87822900

H -8.41886200 -1.18470100 -1.96862200

H -7.44873600 -2.53451200 -1.37279700

H -7.01772600 -1.73376500 -2.89467400

C -6.51083100 0.79685600 -1.84385400

H -6.13816900 0.69932800 -2.87004600

H -5.88828000 1.53371100 -1.32569200

H -7.52940400 1.20334700 -1.90459400

C -7.09948400 -0.35200800 0.31412800

H -8.11948000 0.05117500 0.25147400

H -6.49776300 0.35239000 0.89792000

H -7.15448600 -1.29328500 0.87279300

N -0.63105900 3.33326700 0.95614800

C 0.19632700 3.48498600 -0.23818800

H 1.25115800 3.37092500 0.05703100

H -0.02694800 2.68252600 -0.93324500

C -0.01377500 4.86533900 -0.87262300

H -1.04170800 4.92467300 -1.25372100

H 0.66021300 4.97806900 -1.72945400

C 0.22604700 5.97819200 0.15824900

H 0.01728800 6.96108300 -0.27875500

H 1.28780400 5.97707000 0.44365200

C -0.63296800 5.75062800 1.41182400

H -1.69499300 5.86037900 1.15791100

H -0.40303900 6.49771600 2.18038000

C -0.40703000 4.34524000 1.98704900

H -1.07633900 4.12935900 2.81942900

H 0.62731200 4.25904300 2.35187400

C -1.52107400 2.31113700 1.18202100

O -2.03839100 2.12453000 2.29802400

O -0.70195100 -1.34037500 -0.86000500

C -1.49559100 -1.58145800 1.29120900

C -2.19859000 -0.97110500 2.34601200

C -0.79297300 -2.78224700 1.49825300

C -2.15269500 -1.55170000 3.60817500

H -2.70678400 -0.02673600 2.20598400

C -0.77829300 -3.35705500 2.76434800

H -0.28575200 -3.25116000 0.66666400

C -1.44936900 -2.74247000 3.82465700

H -2.66621200 -1.06466600 4.43139000

H -0.24365200 -4.28947200 2.91943500

H -1.43122600 -3.19109700 4.81368800

N -1.63130400 -1.11288200 -0.04004300

Rh 3.69470800 0.03164800 -0.11119600

C 3.49208300 -1.86647800 -2.29201300

O 4.33285100 -1.13175600 -1.68019900

C 2.15694700 1.51493600 -2.04045500

O 1.20545000 0.69427300 -1.82746400

C 1.71058300 0.92616500 1.79168800

O 0.85201300 0.27269400 1.10995600

C 3.11293500 -2.36467200 1.37482100

O 4.03550600 -1.51011100 1.19416000

Rh 1.47926600 -0.84783400 -0.48727400

O 1.97022700 -2.36303000 0.80871800

O 3.27551600 1.53588500 -1.43862900

O 2.92898800 1.10520000 1.46892400

O 2.24634400 -1.95970000 -2.03713900

C 4.01774200 -2.73512400 -3.41036800

H 4.98572100 -2.37034800 -3.75519500

H 4.13601100 -3.75687600 -3.03444300

H 3.29792200 -2.76239900 -4.23094700

C 3.37516900 -3.49606500 2.33944100

H 3.39288400 -4.44060400 1.78706200

H 4.32572200 -3.35302700 2.85319000

H 2.55475200 -3.54990100 3.06026200

C 1.25087800 1.48145400 3.11534400

H 1.49568900 0.73908300 3.88369200

H 1.79448500 2.39769600 3.35135300

H 0.17273600 1.64413800 3.12324000

C 1.94339600 2.54366900 -3.12554100

H 0.87924200 2.72039900 -3.28666700

H 2.45695500 3.47197500 -2.86973000

H 2.37679500 2.16074500 -4.05560700

C-ts3

0 1

C 2.31906400 0.67604300 0.68263600

C 2.22342400 -0.73191200 1.00610100

C 1.90481800 0.61715400 2.03466700

O 3.24269000 -1.59402300 1.21523900

H 2.61551700 0.39638300 2.83037500

H 0.91650200 0.96083700 2.34707500

Si 4.94548800 -1.43190100 1.28384500

C 5.45633100 -3.23748400 1.26257600

H 6.52432800 -3.36078700 1.46678100

H 5.24244800 -3.68869000 0.28807600

H 4.89482700 -3.79545000 2.01812000

C 5.73962100 -0.47196400 -0.16899000

C 5.36290200 -0.61722000 2.92942000

H 6.44751700 -0.59786800 3.08562800

H 4.91461800 -1.16805200 3.76264300

H 5.00915400 0.41788300 2.97129500

C 7.20552200 -0.95851800 -0.27857800

H 7.72814100 -0.38628600 -1.05642100

H 7.26837800 -2.01636600 -0.55317700

H 7.76481100 -0.81793200 0.65501000

C 5.74936100 1.05040600 0.08229400

H 6.32381400 1.31718100 0.97644500

H 4.73490800 1.44122500 0.20303500

H 6.20840700 1.56977300 -0.76987300

C 5.01117400 -0.76132000 -1.49699200

H 5.59202600 -0.35707200 -2.33743400

H 4.02675400 -0.28818600 -1.52627500

H 4.88543300 -1.83582900 -1.67621200

N 1.54833000 2.70548400 -0.42498900

C 2.03495700 3.59230800 0.63750100

H 1.23358800 4.29940400 0.88086800

H 2.24467900 3.01853300 1.53762500

C 3.28124300 4.36632300 0.17373100

H 4.10336500 3.65463000 0.02864000

H 3.58991900 5.06236600 0.96298600

C 3.00874400 5.11662800 -1.13920300

H 3.92372800 5.60089900 -1.49830600

H 2.28083600 5.91893900 -0.95036000

C 2.44289900 4.16699200 -2.20676600

H 3.19816000 3.42391300 -2.49092700

H 2.16924800 4.71947000 -3.11309500

C 1.21757200 3.41930700 -1.66229300

H 0.83384000 2.69826500 -2.38124800

H 0.41602100 4.13246400 -1.42641000

C 1.88261800 1.37042700 -0.52625000

O 1.88639000 0.78245500 -1.62070900

O -0.03179600 -1.09471900 1.44609700

C 0.91258100 -2.41669800 -0.24529400

C 1.65756400 -2.34733200 -1.42949600

C 0.03557400 -3.47850200 0.00641300

C 1.53615000 -3.37555700 -2.35744900

H 2.24285500 -1.46181100 -1.63422500

C -0.07514100 -4.49690100 -0.93575100

H -0.54560900 -3.48179500 0.91911400

C 0.67618300 -4.45284300 -2.11349000

H 2.09731400 -3.32519300 -3.28569500

H -0.74873100 -5.32730300 -0.74727800

H 0.58605600 -5.25094200 -2.84476200

N 1.01418500 -1.38144800 0.71771700

Rh -3.78771600 0.67498900 -0.46356000

C -1.99116400 2.52053900 0.85239600

O -3.09139800 2.48240100 0.21891700

C -1.51930400 0.37651000 -2.23039900

O -0.91838200 -0.18678600 -1.25708700

C -3.67059000 -2.19559500 -0.75356400

O -2.60644800 -2.16572100 -0.05049500

C -4.12964500 -0.10443900 2.30656400

O -4.76415000 0.39603800 1.32345900

Rh -1.85187800 -0.35696100 0.54379800

O -2.91739400 -0.49864300 2.30135800

O -2.68774100 0.87966400 -2.19357600

O -4.37336600 -1.19306800 -1.09094500

O -1.23203500 1.52981400 1.10928100

C -1.54218900 3.88037400 1.33563900

H -2.39215500 4.41535100 1.76484900

H -0.74144400 3.78389200 2.06872400

H -1.18392000 4.46062400 0.47953700

C -4.87129200 -0.21523200 3.61879000

H -4.63365300 0.66148000 4.23083400

H -5.94796600 -0.23797200 3.44569900

H -4.54536800 -1.10464100 4.16114000

C -4.12766700 -3.56244100 -1.20607800

H -4.91361700 -3.47598400 -1.95657000

H -3.27338700 -4.11620100 -1.60373400

H -4.51035300 -4.11464900 -0.34175700

C -0.77145400 0.41052300 -3.53975500

H -0.91530500 -0.55210400 -4.04292800

H -1.16241700 1.20110500 -4.18211600

H 0.29394400 0.53683200 -3.34191800

C-ts4

0 1

C 3.11060900 0.07287700 -0.70391400

C 1.76647400 0.58887400 -0.84979500

C 3.94579900 -0.70208700 -1.56611100

O 1.40250200 1.58786800 -0.08319400

H 3.54361200 -1.45124400 -2.25008000

H 4.93430500 -0.34558500 -1.84254500

Si 1.80176600 3.25392700 -0.00558100

C 3.66790500 3.43983900 -0.17941400

H 3.95381100 4.49389000 -0.09649700

H 4.02680800 3.07361800 -1.14681300

H 4.17001000 2.87985800 0.61309200

C 0.90214000 4.16469600 -1.41555600

C 1.18032000 3.70502200 1.69612700

H 1.16175200 4.78641600 1.86461500

H 1.80605800 3.23872500 2.46324500

H 0.16533100 3.31235600 1.78948500

C 1.39025400 5.63122200 -1.42676700

H 0.86166100 6.19422600 -2.20766300

H 2.46282800 5.71033500 -1.63810600

H 1.19500100 6.14120800 -0.47510400

C -0.62558500 4.13128200 -1.20380900

H -0.91355000 4.62225400 -0.26708500

H -1.00618100 3.10846300 -1.17990500

H -1.12447300 4.66840200 -2.02287200

C 1.23352800 3.50502000 -2.77179400

H 0.80303600 4.09827700 -3.59022900

H 0.79688500 2.50408700 -2.83073700

H 2.31396700 3.44172900 -2.95409000

N 4.76021900 -1.06790700 0.28770700

C 6.17029600 -0.82412100 0.66854800

H 6.77426000 -1.55001400 0.10932500

H 6.43832700 0.17921500 0.32471900

C 6.41422200 -0.97998500 2.17888800

H 5.88419500 -0.17425700 2.69462300

H 7.48596700 -0.86309100 2.37582100

C 5.90786100 -2.34099900 2.67474600

H 6.02312200 -2.41447500 3.76110300

H 6.51593000 -3.14507800 2.23574900

C 4.43789400 -2.53765500 2.27870800

H 3.81555100 -1.79389900 2.78587000

H 4.07627200 -3.52844600 2.57653100

C 4.26170100 -2.38477200 0.76238500

H 3.21395400 -2.46607400 0.46276700

H 4.83280200 -3.15748600 0.23253300

C 3.80968600 0.07233800 0.54337100

O 3.77355300 0.67276800 1.60454200

O -0.34817800 0.68175000 -1.80819800

C 1.03249100 -1.15380400 -2.35502500

C 1.10482000 -1.15682800 -3.74732800

C 1.09531500 -2.33944400 -1.62051300

C 1.27751200 -2.37012400 -4.41554600

H 1.01933800 -0.21889700 -4.28568100

C 1.27826400 -3.54514400 -2.29576700

H 0.97052000 -2.28911300 -0.54567100

C 1.37277900 -3.56179400 -3.69160000

H 1.33800300 -2.38446200 -5.49950800

H 1.32879000 -4.47436900 -1.73533000

H 1.50994500 -4.50371400 -4.21458700

N 0.85355400 0.09140200 -1.66825800

Rh -3.17304000 -0.90818800 1.46783800

C -0.34952500 -1.17639000 2.07483600

O -1.49218400 -1.35239200 2.59185600

C -2.47689600 1.87378300 1.79757900

O -1.68602800 1.74706900 0.80670400

C -4.49733300 0.27953300 -0.81573400

O -3.34873900 0.59614400 -1.26673900

C -2.45617400 -2.80830600 -0.59086700

O -3.05554900 -2.73848100 0.52754900

Rh -1.68829700 -0.03817600 -0.24368900

O -1.82787300 -1.86495100 -1.17275900

O -3.21954100 0.96832500 2.29338000

O -4.73860800 -0.37628500 0.24796300

O -0.11169800 -0.73719800 0.89967100

C 0.86956300 -1.52502500 2.90051800

H 0.59540200 -1.69637500 3.94168500

H 1.32451100 -2.43669400 2.49769100

H 1.60408800 -0.71910500 2.81922000

C -2.50556900 -4.12835400 -1.32696900

H -1.56601100 -4.29486200 -1.85808200

H -2.71562600 -4.94458100 -0.63435300

H -3.30877200 -4.08369900 -2.07016500

C -5.68946100 0.76247200 -1.61019000

H -6.55248600 0.12087500 -1.42651700

H -5.93685700 1.77892200 -1.28541400

H -5.44646900 0.79454900 -2.67365800

C -2.52110200 3.22775000 2.47190600

H -2.28090000 4.01608600 1.75682300

H -3.50542900 3.39249200 2.91303100

H -1.77822600 3.25029800 3.27636000

C-ts5

0 1

C -1.73679500 -1.37103400 -0.94326600

C -1.68088900 0.08883200 -0.78774500

C -2.68270000 -1.93844400 -2.06922200

O -2.69185200 0.72814700 -0.18828700

H -2.76585800 -1.14792400 -2.82270500

H -2.16023500 -2.77476700 -2.54412700

Si -2.86386600 1.03082200 1.50585200

C -1.27152300 1.82359800 2.08979900

H -1.31679700 2.05475300 3.16016300

H -1.08735700 2.75241100 1.54201200

H -0.42125300 1.16329400 1.90370600

C -4.35942600 2.20317800 1.61785700

C -3.22298700 -0.61771900 2.33342700

H -3.11642200 -0.54715400 3.42103300

H -2.53466400 -1.38509000 1.97215400

H -4.24088800 -0.95587300 2.11573300

C -4.82255800 2.24557600 3.09243200

H -5.67526500 2.92888700 3.19881000

H -4.03365600 2.60834400 3.76247900

H -5.14490100 1.26207500 3.45152200

C -5.51782600 1.68921200 0.73794300

H -5.85180500 0.68955900 1.03788600

H -5.23414600 1.64202300 -0.31824300

H -6.38280800 2.36031900 0.82356700

C -3.97313200 3.62845400 1.17140500

H -4.84743100 4.29078300 1.22717600

H -3.60301300 3.65219900 0.14259400

H -3.19631300 4.05940500 1.81195500

N -4.00392700 -2.37912100 -1.69653600

C -4.91451400 -1.31337300 -1.27442000

H -4.60956800 -0.89448400 -0.30356300

H -4.85348900 -0.49665100 -2.00302900

C -6.34970400 -1.83422000 -1.16702800

H -6.69151600 -2.14900200 -2.16063200

H -7.00640900 -1.02182700 -0.83392500

C -6.41417100 -3.02238300 -0.19825000

H -7.42906000 -3.43272600 -0.15096200

H -6.16367600 -2.67613200 0.81536600

C -5.41038400 -4.10162500 -0.62467800

H -5.71386100 -4.52266500 -1.59102000

H -5.38724300 -4.92344200 0.10007100

C -4.00488800 -3.50834100 -0.76546000

H -3.30724100 -4.26665000 -1.13974200

H -3.63903600 -3.19928400 0.23296500

C -0.99680500 -2.28252300 -0.34024900

O -0.49081900 -3.24053000 0.10437900

O 0.30633000 0.20407100 -1.92862600

C -0.87563600 2.22164600 -1.71681900

C -2.11406800 2.82778300 -1.96162300

C 0.31251200 2.93726500 -1.88974500

C -2.15923300 4.17440800 -2.31902100

H -3.03011300 2.25915900 -1.87881300

C 0.24965800 4.28571300 -2.23788300

H 1.26166900 2.43900700 -1.76232200

C -0.98040600 4.91229600 -2.44489500

H -3.12119500 4.64129900 -2.50815900

H 1.17343500 4.84284300 -2.36201000

H -1.02084200 5.96196900 -2.72030400

N -0.77196500 0.80304400 -1.42817900

Rh 3.71349200 -0.61104100 1.05893700

C 2.56795200 2.02797200 1.38728300

O 3.48244500 1.27985300 1.85000100

C 1.09744100 -1.26768600 2.12033000

O 0.59387200 -0.78081600 1.04415500

C 2.97098400 -2.78399400 -0.69734900

O 2.07904200 -2.01841500 -1.18977500

C 4.49209000 0.54821900 -1.47090600

O 4.98156200 0.11863000 -0.37870100

Rh 1.88025900 -0.12190600 -0.44329400

O 3.25640300 0.57177000 -1.78781700

O 2.32852300 -1.32475500 2.40153700

O 3.80894400 -2.47652500 0.20989600

O 1.76720500 1.74069800 0.43523100

C 2.37403300 3.38409600 2.02504600

H 3.17499700 3.59747000 2.73285500

H 2.33366200 4.15237900 1.24856100

H 1.41022600 3.39168800 2.54413500

C 5.45976400 1.11890200 -2.48108300

H 6.43082100 0.62991800 -2.38933900

H 5.06180400 1.00823700 -3.49086200

H 5.59239100 2.18670100 -2.27555000

C 3.00554500 -4.19428600 -1.23494100

H 3.88229200 -4.72718800 -0.86666600

H 2.09571000 -4.70849400 -0.90941400

H 3.00032300 -4.17150600 -2.32747800

C 0.12152400 -1.81189900 3.13423500

H -0.47276700 -2.60266700 2.66842800

H 0.65305500 -2.20514200 4.00033800

H -0.56139000 -1.01713300 3.44553700

Di-Rh-ts4

0 1

C 1.14964000 1.29914100 2.27643800

C 0.21056400 0.38789400 1.62529200

C 0.92257900 2.53166800 2.95998700

O 0.41061200 -0.87851100 1.86724100

H 0.24492300 3.28827200 2.55500400

H 1.11005600 2.63290900 4.02441100

Si -0.63958400 -2.25280900 2.04768600

C -1.02582100 -3.07348000 0.41140900

H -1.30835900 -4.11961200 0.57945000

H -0.12521800 -3.06212100 -0.20980100

H -1.82822000 -2.56246800 -0.11659000

C 0.51467600 -3.39239400 3.06186200

C -2.07446300 -1.68050700 3.11385600

H -2.78748000 -2.49756600 3.26102600

H -2.61360100 -0.85891100 2.64008600

H -1.71070100 -1.36475500 4.09818900

C -0.32369000 -4.54575000 3.65522800

H 0.32325000 -5.24140500 4.20731000

H -0.83392500 -5.12926300 2.87874500

H -1.08493200 -4.18249400 4.35527200

C 1.18867800 -2.61000900 4.20831300

H 0.45516400 -2.16109300 4.88910500

H 1.82682400 -1.81189900 3.81689900

H 1.81773400 -3.28319300 4.80737500

C 1.61483000 -3.97448700 2.15055300

H 2.29136300 -4.61503700 2.73463000

H 2.22052400 -3.19005700 1.68803400

H 1.19104000 -4.59536200 1.35220200

N 3.01289900 2.40202000 2.84012800

C 3.90323700 2.32813500 4.01965000

H 3.80804400 3.29009700 4.54048700

H 3.51886500 1.54479400 4.67948800

C 5.37164600 2.07554900 3.64239400

H 5.44629800 1.08219500 3.18917300

H 5.97358400 2.08127600 4.55844000

C 5.85634200 3.13910500 2.64937900

H 6.88713600 2.92870100 2.34470900

H 5.86238200 4.12558200 3.13594300

C 4.93665400 3.15925400 1.42261100

H 5.02124700 2.21219200 0.88807100

H 5.22275300 3.95623000 0.72534400

C 3.47303200 3.38566200 1.82321800

H 2.81218400 3.29757900 0.95959500

H 3.34987900 4.37956900 2.27244400

C 2.53838300 1.08976000 2.32925900

O 3.30986300 0.17135800 2.02642500

O -1.57638600 -0.13187900 0.29933700

C -0.84173600 2.16128600 0.40468000

C -1.84474400 2.99887500 0.88910500

C 0.11634400 2.60936400 -0.50675100

C -1.87911500 4.32554700 0.45594600

H -2.59421500 2.59782200 1.56147500

C 0.06773100 3.93617200 -0.93424900

H 0.88850000 1.92678700 -0.84424300

C -0.92621300 4.79427200 -0.45189100

H -2.65719400 4.98928200 0.82116600

H 0.80487700 4.29946900 -1.64311500

H -0.95878500 5.82704600 -0.78709000

N -0.77258800 0.79442700 0.85092600

Rh 4.23327100 -0.80999100 -2.51474300

C 4.10989300 -3.11701300 -0.79382300

O 4.32674000 -2.79008300 -2.00466900

C 1.38640000 -0.85790300 -2.03773900

O 1.65128800 -0.59903000 -0.81301500

C 3.69676800 1.98617600 -2.00543100

O 3.38725100 1.65331600 -0.81211800

C 6.46105100 -0.23012200 -0.76473100

O 6.20006300 -0.56947500 -1.96165800

Rh 3.61040200 -0.32590600 -0.24392600

O 5.60707800 -0.02508900 0.16062300

O 2.25404600 -1.00903800 -2.96017800

O 4.10205800 1.20388500 -2.91673300

O 3.83290300 -2.31577600 0.15821400

C 4.15936400 -4.58381100 -0.44433700

H 4.61022800 -5.15695700 -1.25454500

H 4.71550000 -4.72276800 0.48540400

H 3.13780100 -4.93588300 -0.27051900

C 7.91506100 -0.07846200 -0.38526100

H 8.23826400 -0.98771600 0.13245700

H 8.53084100 0.06008300 -1.27444500

H 8.03574600 0.75965800 0.30453600

C 3.55724100 3.44506000 -2.37543600

H 4.48369700 3.79294000 -2.83900000

H 2.76099800 3.54944900 -3.11896600

H 3.32378600 4.04966800 -1.49859300

C -0.06027400 -1.00662700 -2.42512000

H -0.72231500 -0.62427100 -1.65021600

H -0.23528900 -0.50207000 -3.37835900

H -0.27570700 -2.07013200 -2.57092000

Rh -6.04307800 0.01940500 -0.75310100

C -5.15801900 -2.30779300 0.70367500

O -6.19419200 -1.81408900 0.15922500

C -5.45166400 1.23669800 1.81364500

O -4.21378000 1.00941200 1.58625400

C -4.60384600 2.34301100 -1.68619400

O -3.55541000 1.83667500 -1.16849000

C -4.29331900 -1.19602200 -2.70871000

O -5.51566700 -0.90854200 -2.50812500

Rh -3.69479100 0.04675100 -0.16618600

O -3.31863800 -0.93377100 -1.92926000

O -6.41974700 0.94230900 1.05091100

O -5.77574600 1.85163300 -1.64463400

O -4.00112000 -1.77506400 0.74401700

C -5.29162200 -3.65366700 1.37842500

H -6.29950200 -4.04986700 1.25508000

H -4.55863400 -4.34526200 0.95375800

H -5.06237000 -3.54512700 2.44282600

C -3.95692100 -1.95015100 -3.97413300

H -3.94000200 -3.02186000 -3.74833800

H -4.71399500 -1.76837400 -4.73805300

H -2.96624900 -1.66317600 -4.33141400

C -4.41782400 3.63796300 -2.44267500

H -5.34795800 4.20809400 -2.45576700

H -3.60642400 4.21709700 -1.99792500

H -4.14171800 3.40140000 -3.47594200

C -5.77124200 1.92657600 3.12042000

H -5.36906000 1.33748400 3.94950900

H -5.28042800 2.90450300 3.14277200

H -6.84773600 2.05135800 3.23633700

OAc-PC

0 1

C -0.74947600 -1.48813400 -0.63465500

C 0.32474100 -0.74087300 -0.27578300

C -1.99405200 -1.05540800 -1.33815000

O 0.56756100 0.54065700 -0.53263400

H -1.80923700 -0.07809800 -1.82532400

H -2.20730000 -1.77811100 -2.13384600

Si 0.96786200 1.82779200 0.54281200

C 2.81212400 1.82077700 0.88826200

H 3.09931000 2.74445700 1.40301200

H 3.39418100 1.74756300 -0.03423500

H 3.09748300 0.97732900 1.52254800

C 0.44192400 3.35162700 -0.46240300

C -0.00848900 1.59175500 2.12806800

H 0.32433200 2.30523000 2.88956500

H 0.14962000 0.58348700 2.52478200

H -1.08119600 1.73261400 1.97423200

C 0.74153400 4.62904200 0.35093100

H 0.43288500 5.51818300 -0.21428300

H 1.80988700 4.73840900 0.56875000

H 0.19856900 4.64591800 1.30327700

C -1.06749700 3.28549200 -0.77303400

H -1.67399800 3.29624700 0.13927600

H -1.32452700 2.38191400 -1.33423300

H -1.36866500 4.15247500 -1.37580400

C 1.23198000 3.38457800 -1.78775400

H 0.93922600 4.25982300 -2.38282500

H 1.03810800 2.49215000 -2.39186200

H 2.31268400 3.45136600 -1.61906300

N -3.16540600 -1.01766700 -0.45900500

C -4.39675100 -0.82957300 -1.22654700

H -4.40425500 0.15550600 -1.74165200

H -4.43658300 -1.59812900 -2.00673000

C -5.62851300 -0.93653800 -0.32371100

H -5.67526600 -1.95443500 0.08160800

H -6.53441300 -0.77660900 -0.91998500

C -5.54775600 0.07226300 0.82899300

H -6.39557600 -0.04900500 1.51288600

H -5.60921700 1.09228700 0.42297700

C -4.21774000 -0.09453800 1.57421700

H -4.19535500 -1.07014400 2.07466000

H -4.10230900 0.67465900 2.34745500

C -3.03762700 -0.02050300 0.60187000

H -2.10338200 -0.20868600 1.13749400

H -2.97124700 1.00625000 0.18080200

C -0.50395800 -2.83765600 -0.16417100

O -1.13254900 -3.86390400 -0.23505000

O 0.74372400 -2.82258300 0.50674800

C 2.64875000 -1.47614600 0.15718400

C 3.12675800 -1.05478800 -1.08677500

C 3.53265200 -1.89866400 1.15347900

C 4.50228200 -1.03138400 -1.31862700

H 2.43227100 -0.73336900 -1.85462100

C 4.90298600 -1.89511400 0.90414800

H 3.13248400 -2.22742700 2.10658000

C 5.39129200 -1.45243700 -0.32844600

H 4.87701900 -0.69495800 -2.28059500

H 5.59082000 -2.22801700 1.67521900

H 6.46047200 -1.43778200 -0.51618400

N 1.25001100 -1.48036200 0.46638600

OAc-ts4-1

0 1

C -1.96282000 -1.15296700 -0.23582600

C -3.05338600 -0.19568300 -0.49328300

C -1.86100000 -1.68097400 1.07017600

O -2.73599200 1.01424400 -0.91299800

H -2.78041700 -1.63082100 1.64403300

H -1.34733500 -2.61841800 1.23892000

Si -3.80453300 2.43091800 -0.92497300

C -4.23210400 3.06200500 0.80461900

H -4.01672600 4.12727900 0.93150200

H -3.64772300 2.51706000 1.55483400

H -5.28531500 2.87482000 1.02438500

C -2.38901700 3.59807200 -1.56732900

C -5.12216300 2.48116900 -2.27180100

H -5.13253300 3.43712500 -2.80448800

H -6.11202600 2.28043100 -1.85853500

H -4.90917300 1.69435500 -3.00353900

C -2.88582300 5.05655000 -1.64964000

H -2.09584200 5.70847400 -2.04888500

H -3.16461800 5.45679500 -0.66852000

H -3.75361100 5.16426800 -2.30995800

C -1.94072200 3.15242700 -2.97424000

H -2.75091900 3.23781900 -3.70701100

H -1.60378800 2.11181600 -2.97298200

H -1.10770900 3.77331900 -3.33525800

C -1.16816700 3.55365300 -0.62519400

H -0.38242100 4.24145700 -0.96785600

H -0.72980700 2.55250100 -0.58513800

H -1.42916400 3.84621900 0.39936000

N 0.32136900 -1.63383400 -1.38137900

C 0.78562200 -2.59713400 -2.46914000

H 1.82829700 -2.81540100 -2.22266300

H 0.20137400 -3.51144900 -2.34196500

C 0.73331400 -2.08369500 -3.91092600

H -0.29786800 -2.03978500 -4.25888700

H 1.26584700 -2.82255400 -4.52199800

C 1.39244400 -0.70579200 -4.03630600

H 1.28500300 -0.32868800 -5.05818100

H 2.46924600 -0.78778300 -3.83256200

C 0.76139400 0.26297900 -3.03225100

H -0.29379900 0.42021200 -3.26662100

H 1.25225800 1.24172800 -3.06386600

C 0.92825800 -0.25978800 -1.61007700

H 0.48105500 0.40628800 -0.87570800

H 1.98754300 -0.34747600 -1.37949800

C -1.21038500 -1.45324200 -1.41793600

O -1.70636200 -1.45489900 -2.53509900

O -5.13975500 0.64944000 -0.21200800

C -4.90009000 -1.66980600 0.12047800

C -4.58108700 -2.80897300 -0.62355000

C -5.82369900 -1.71958500 1.16532000

C -5.16886200 -4.02468900 -0.27804900

H -3.89489200 -2.72757000 -1.45975700

C -6.40446400 -2.94211500 1.49866400

H -6.07831400 -0.79987100 1.67874200

C -6.07413400 -4.09643000 0.78435000

H -4.93138500 -4.91531200 -0.85196200

H -7.12032300 -2.99157000 2.31345700

H -6.53204600 -5.04595200 1.04415400

N -4.32291000 -0.39976900 -0.20476500

C 1.25164500 -1.37108100 1.15415600

C 2.44215100 -0.59696000 1.25774000

C 0.93365900 -2.25477500 0.03625100

O 2.58339000 0.35307900 2.05011500

H 1.82760100 -2.72496900 -0.35771200

H 0.22650000 -3.05188000 0.25481200

Si 4.89992800 1.49999800 0.21549400

C 6.08113300 1.76893100 -1.23141700

H 6.80587100 2.56354900 -1.02733200

H 5.54129000 2.03996400 -2.14503700

H 6.63258800 0.84386300 -1.42939200

C 3.87949100 3.09232300 0.57774700

C 5.79889100 0.79497200 1.69918500

H 6.52241400 1.51178800 2.10204300

H 6.33875300 -0.11579100 1.42057700

H 5.07280700 0.54524900 2.47568600

C 3.78231500 3.34453100 2.09809400

H 3.19118700 4.25106000 2.28981000

H 4.77067300 3.50102500 2.54598200

H 3.30424400 2.50810900 2.61026900

C 2.45389100 2.95066900 0.00074900

H 1.91712400 2.13309000 0.48584000

H 2.45989200 2.76113000 -1.07956000

H 1.88738700 3.87685900 0.16634500

C 4.56243500 4.31287100 -0.07968800

H 3.99380600 5.22530900 0.14476800

H 4.61683600 4.22106800 -1.16973300

H 5.58206800 4.46802400 0.29296200

N -0.87700800 -0.64077000 2.32675300

C -1.64083800 -0.69138700 3.60888500

H -2.67371300 -0.42207400 3.35792500

H -1.62313200 -1.72288000 3.96343100

C -1.09847700 0.27582500 4.66527800

H -0.10006600 -0.05432100 4.96397100

H -1.74499800 0.21370000 5.54820800

C -1.04600800 1.70784800 4.11785700

H -0.60012600 2.38089400 4.85743600

H -2.06781300 2.06993200 3.93189000

C -0.24225900 1.73813600 2.81349200

H 0.80529100 1.48043200 2.99337000

H -0.25980800 2.73391700 2.35777900

C -0.81536000 0.75355200 1.79440800

H -0.21852900 0.72870500 0.88755700

H -1.83394500 1.02972800 1.51359600

C 0.44144400 -1.32922800 2.39099600

O 0.75680200 -1.83404000 3.44774400

O 3.85683200 0.28886600 -0.44129000

C 4.02323400 -2.04569400 -0.11830500

C 3.98070100 -3.17891600 0.72142500

C 4.57744400 -2.16474800 -1.40619100

C 4.44044900 -4.40511300 0.25426000

H 3.56965500 -3.08201400 1.72047500

C 5.05388100 -3.40129000 -1.84560500

H 4.63684300 -1.28746700 -2.03753000

C 4.98181400 -4.53050000 -1.03007900

H 4.39333700 -5.26949800 0.91064800

H 5.48240000 -3.47639000 -2.84137000

H 5.35198600 -5.48874700 -1.38008100

N 3.53217400 -0.83173000 0.35025300

OAc-ts4-2

0 1

C -0.92062200 0.42822700 1.09460300

C -1.83431500 -0.61634500 0.63492300

C -0.39761600 0.56627600 2.41857200

O -2.59009900 -0.32023400 -0.39554300

H -0.72264600 -0.06591600 3.24277900

H 0.54007300 1.08196200 2.57397700

Si -3.59965300 -1.44263000 -1.31840200

C -5.28242200 -1.84099500 -0.56038700

H -6.09621600 -1.74256700 -1.28566300

H -5.48164000 -1.14068000 0.25838400

H -5.29031300 -2.84714000 -0.13666300

C -3.95427200 -0.12133200 -2.69220600

C -2.60486800 -2.80574400 -2.15678100

H -2.79008700 -2.86339400 -3.23421700

H -2.80291500 -3.77484100 -1.69455200

H -1.54536200 -2.57690800 -2.00120600

C -4.87909300 -0.70561700 -3.77992200

H -5.05447400 0.03644900 -4.57234700

H -5.86137000 -0.98973000 -3.38537000

H -4.44676200 -1.59099600 -4.26136600

C -2.63152900 0.32279400 -3.34881100

H -2.11637600 -0.51872400 -3.82966200

H -1.94832200 0.75926000 -2.61680900

H -2.82184200 1.07391900 -4.13069300

C -4.63293200 1.12050800 -2.07532800

H -4.86056900 1.86439300 -2.85321700

H -3.97943200 1.60077400 -1.34019500

H -5.58001200 0.86733900 -1.58288100

N -1.24099400 2.48768800 2.06814000

C -0.59038100 3.81657500 2.13347100

H -0.38508400 4.00169600 3.19594000

H 0.36225100 3.72924000 1.60683600

C -1.46089300 4.94723500 1.55881400

H -1.54292900 4.80213200 0.47813000

H -0.95338800 5.90323200 1.73332800

C -2.85924300 4.94572700 2.19312600

H -3.49147500 5.70409400 1.71958100

H -2.78210400 5.21538500 3.25650900

C -3.50402000 3.55780300 2.06852400

H -3.67574900 3.31702400 1.01351200

H -4.47591200 3.52767200 2.57400700

C -2.59580400 2.48604300 2.68430400

H -3.02021900 1.48460100 2.56361500

H -2.45836300 2.67148000 3.75661800

C -1.24712300 1.82687000 0.72684300

O -1.50154900 2.41850500 -0.28961300

O -2.91430100 -2.61068500 0.60482800

C -1.32919400 -2.35975400 2.30590000

C -0.00383300 -2.78413800 2.24270500

C -2.08408500 -2.50474300 3.47381300

C 0.58820600 -3.31918600 3.38976400

H 0.54477900 -2.67581500 1.31753600

C -1.48219100 -3.03698000 4.61169200

H -3.12726400 -2.20632900 3.46841300

C -0.14178800 -3.43971500 4.57251400

H 1.62171800 -3.64966100 3.35039700

H -2.05767300 -3.14509000 5.52628100

H 0.32455900 -3.85679100 5.46025900

N -1.98404900 -1.82457900 1.14668300

Rh 3.27990400 -0.09265300 -1.25340500

C 1.14912500 1.04172900 -2.81073000

O 2.38749800 0.75089300 -2.88521700

C 2.91074000 2.42029700 0.15178500

O 1.77214200 1.98386100 0.53977300

C 3.37072300 -1.01169500 1.49249700

O 2.14265300 -0.67780200 1.59287100

C 1.54747300 -2.39368900 -1.50481100

O 2.67800700 -1.95314300 -1.87845600

Rh 1.17706800 0.10164500 -0.06845600

O 0.71941600 -1.78344800 -0.74460200

O 3.74677100 1.79060600 -0.56143700

O 4.08709300 -0.92115300 0.44907000

O 0.40161700 0.87176900 -1.79392400

C 0.49734000 1.62174400 -4.04080400

H 1.25258100 2.03600500 -4.70948500

H -0.04445300 0.82508900 -4.55958000

H -0.23289400 2.37882500 -3.75087000

C 1.12004400 -3.74769100 -2.01447300

H 0.53421600 -3.60697800 -2.92884500

H 1.99370000 -4.35694100 -2.24945900

H 0.47922800 -4.24186200 -1.28235100

C 4.03718000 -1.54376900 2.74000700

H 4.67532500 -2.39295000 2.48597600

H 4.67850500 -0.76096100 3.15823400

H 3.29042900 -1.82827500 3.48190500

C 3.26689800 3.82650500 0.57394000

H 3.04296100 3.96592800 1.63458400

H 4.31925300 4.03088700 0.37712500

H 2.64998100 4.53059700 0.00584500

Rh_2_(cap)_4_

0 1

Rh -0.00000700 0.75160300 0.93351300

C 1.94307400 -1.40002300 1.18638200

N 1.47463600 -1.79306600 0.02012900

O 1.46872100 -0.39309300 1.83542700

C 3.13489400 -2.08109400 1.83771600

H 3.18975200 -1.69745400 2.85853800

H 2.96958200 -3.16443800 1.89902800

C 4.45307500 -1.79725700 1.08844900

H 5.29083300 -2.00943100 1.76341700

H 4.50418000 -0.72510900 0.86181500

C 4.64081600 -2.62180300 -0.19461700

H 4.71163400 -3.68312500 0.08553900

H 5.60591800 -2.36091100 -0.64680900

C 3.53368200 -2.47371100 -1.24927000

H 3.47823700 -1.44523600 -1.62466800

H 3.78423600 -3.11451000 -2.10478300

C 2.13295300 -2.85684800 -0.74101800

H 2.18198400 -3.78842100 -0.15643500

H 1.48819400 -3.07491000 -1.59947100

C 1.94306900 1.40031000 -1.18646800

N 1.47461500 1.79336000 -0.02020600

O 1.46862000 0.39348000 -1.83558000

C 3.13503500 2.08120800 -1.83769900

H 3.18984500 1.69768000 -2.85856800

H 2.96998500 3.16459800 -1.89886800

C 4.45310700 1.79691800 -1.08839900

H 5.29096400 2.00896100 -1.76328400

H 4.50389500 0.72472300 -0.86190400

C 4.64098400 2.62122900 0.19479100

H 5.60602000 2.36006200 0.64696700

H 4.71204300 3.68257900 -0.08520100

C 3.53380700 2.47320900 1.24939800

H 3.78451200 3.11376900 2.10504300

H 3.47807600 1.44466400 1.62457500

C 2.13318700 2.85680800 0.74120500

H 2.18244800 3.78852200 0.15686200

H 1.48846700 3.07479900 1.59970500

C -1.94307900 1.40035000 -1.18647500

N -1.47473300 1.79328400 -0.02013500

O -1.46858300 0.39356000 -1.83562700

C -3.13492200 2.08139500 -1.83778200

H -2.96974900 3.16477600 -1.89882000

H -3.18963200 1.69798300 -2.85869900

C -4.45313400 1.79719500 -1.08869100

H -4.50409700 0.72499100 -0.86228300

H -5.29086600 2.00939200 -1.76368400

C -4.64109500 2.62142000 0.19453900

H -5.60621700 2.36032000 0.64657000

H -4.71199700 3.68280300 -0.08536700

C -3.53406300 2.47317300 1.24926400

H -3.78480300 3.11366100 2.10495300

H -3.47848400 1.44458000 1.62433400

C -2.13333600 2.85669500 0.74129600

H -1.48872600 3.07453600 1.59991400

H -2.18243700 3.78847800 0.15704900

C -1.94295000 -1.40017000 1.18642700

N -1.47464500 -1.79305200 0.02005200

O -1.46859400 -0.39327300 1.83550000

C -3.13462800 -2.08139100 1.83784600

H -2.96922300 -3.16472900 1.89902700

H -3.18937900 -1.69785100 2.85871300

C -4.45291300 -1.79758100 1.08874600

H -4.50407200 -0.72542800 0.86211800

H -5.29059100 -2.00980100 1.76379700

C -4.64074100 -2.62212000 -0.19431200

H -5.60594300 -2.36134000 -0.64635500

H -4.71138300 -3.68346000 0.08582400

C -3.53380100 -2.47383600 -1.24912500

H -3.78440300 -3.11458700 -2.10465700

H -3.47854500 -1.44531700 -1.62442800

C -2.13294500 -2.85683300 -0.74112000

H -1.48827400 -3.07470200 -1.59968100

H -2.18178100 -3.78848700 -0.15664500

Rh -0.00001700 -0.75127300 -0.93365400

cap-cb

0 1

C -2.54119000 -0.93987700 -1.16991900

C -2.25516000 -1.07311000 -2.48572700

O -3.76106500 -1.28470900 -0.64825100

H -3.00111400 -1.42652600 -3.18926100

H -1.27090400 -0.80948600 -2.85055600

Si -5.29477400 -0.71474200 -1.12826300

C -6.03779000 -2.01265200 -2.27455000

H -7.03497500 -1.71751200 -2.61807500

H -6.13106700 -2.97678900 -1.76327800

H -5.41014500 -2.16441800 -3.15924000

C -6.30297200 -0.56290400 0.48783300

C -5.06389600 0.93891000 -1.98830000

H -6.02925300 1.42549200 -2.16492200

H -4.55633100 0.83473900 -2.95208800

H -4.45695400 1.58697000 -1.34835100

C -7.80262800 -0.79385400 0.20025000

H -8.38669100 -0.66074100 1.12093700

H -8.00209000 -1.80529900 -0.17031000

H -8.19695800 -0.08271400 -0.53638400

C -6.12234400 0.84546400 1.09575900

H -6.55950400 1.61915800 0.45461900

H -5.06995300 1.10119400 1.24199200

H -6.62953000 0.90229000 2.06897200

C -5.81145700 -1.62000700 1.49962400

H -6.39362700 -1.55229900 2.42908800

H -4.75604900 -1.47187500 1.74248700

H -5.92352300 -2.64118100 1.11587900

N -2.33679200 -0.19953500 2.14736300

C -1.81276200 -1.51654900 2.50471400

H -2.66164200 -2.17159400 2.75344800

H -1.29206400 -1.93832000 1.64321200

C -0.85404500 -1.41205600 3.69612700

H 0.04327100 -0.88690800 3.35595000

H -0.55259600 -2.42149000 3.99889400

C -1.49538200 -0.66062700 4.87022200

H -0.77811900 -0.55301400 5.69190300

H -2.34274000 -1.24142000 5.26296200

C -1.99537900 0.71477600 4.40693700

H -1.14380100 1.32769400 4.08736800

H -2.50006600 1.24603500 5.22233800

C -2.96081900 0.56856400 3.22596800

H -3.24944100 1.53697300 2.81753600

H -3.87361600 0.04575900 3.54694800

C -2.28336900 0.30693500 0.90216100

O -2.78238700 1.39406100 0.56209500

Rh 2.79361700 0.45600200 -0.51701000

C 1.23987000 2.12324100 -2.27505200

N 0.17766000 1.45281600 -1.85048000

O 2.44619700 1.87497000 -1.92942000

C 1.12446600 3.33595700 -3.19188700

H 2.12085000 3.47753100 -3.61560700

H 0.43109600 3.15277700 -4.02055200

C 0.69601200 4.59814400 -2.41591900

H 0.96707500 5.48366100 -3.00361300

H 1.28156400 4.65117000 -1.48979300

C -0.80525600 4.64454100 -2.10220500

H -1.35074800 4.75712800 -3.05097800

H -1.02421600 5.54648600 -1.51612200

C -1.35436000 3.41112400 -1.37353000

H -0.92332500 3.30025900 -0.37375600

H -2.43249000 3.54046000 -1.22416600

C -1.13562200 2.08161400 -2.11516400

H -1.27976000 2.21979900 -3.19699800

H -1.90643900 1.39768000 -1.79473600

C 1.27639100 2.06266600 1.41731600

N 2.48605800 1.82961700 0.96791800

O 0.23472800 1.39485000 1.04686800

C 0.96796800 3.19488100 2.38558000

H -0.06988200 3.05975600 2.69712500

H 1.59855300 3.11570200 3.28106600

C 1.14921700 4.58597300 1.74255300

H 0.60444100 5.32101400 2.34730500

H 0.66971600 4.58311100 0.75661200

C 2.61109700 5.04121300 1.62151100

H 2.63473100 6.04378100 1.17621500

H 3.02464100 5.14698000 2.63546100

C 3.53248800 4.10989400 0.81931700

H 4.54209000 4.54117500 0.81666000

H 3.21106300 4.04059800 -0.22645400

C 3.60886400 2.67889400 1.37673800

H 3.70342900 2.70768600 2.47383300

H 4.51667800 2.19516900 1.00493500

C 2.15105800 -1.65502100 1.41931200

N 3.13916100 -0.98377400 0.88629000

O 0.91179000 -1.47874500 1.07972600

C 2.36664500 -2.72078000 2.48641800

H 2.81549900 -2.25347300 3.37432800

H 1.37574900 -3.07241700 2.77681900

C 3.23488400 -3.91817500 2.04596600

H 2.89099700 -4.27573300 1.06860300

H 3.05963100 -4.73909000 2.75191400

C 4.74083800 -3.63607300 1.98788700

H 5.26573600 -4.56091000 1.71791000

H 5.08854200 -3.36886200 2.99668100

C 5.14746900 -2.51851600 1.02018300

H 6.23958900 -2.41218300 1.04537700

H 4.87692700 -2.77862800 -0.01051300

C 4.51948000 -1.15599200 1.34950200

H 5.10567800 -0.37112200 0.86282800

H 4.59110800 -0.96619500 2.43226700

C 2.02744900 -1.72742400 -2.28212200

N 0.83569600 -1.68793600 -1.69891600

O 2.95824600 -0.87628200 -2.07911300

C 2.41559800 -2.84609900 -3.23991100

H 1.58630800 -3.09740600 -3.91249000

H 3.23185200 -2.44621500 -3.84537900

C 2.88105300 -4.11047600 -2.49219100

H 3.56121100 -3.81066300 -1.68473300

H 3.46528900 -4.73695900 -3.17670400

C 1.71644300 -4.93086000 -1.92806300

H 2.11045400 -5.80122900 -1.38771700

H 1.13065000 -5.33073000 -2.76872000

C 0.77204500 -4.14611200 -1.00917200

H -0.00450100 -4.83430200 -0.64912200

H 1.29892100 -3.78241300 -0.12408900

C 0.05867900 -2.94237700 -1.64982400

H -0.82086400 -2.73948700 -1.04988700

H -0.31227300 -3.21856900 -2.64912500

Rh 0.38621000 -0.09184900 -0.39293300

C -1.57925900 -0.38359600 -0.22235800

Rh_2_(OAc)_4_

0 1

Rh 0.00001100 0.00000000 1.19218600

C 1.86277000 -1.85366800 0.00000200

O 1.44691000 -1.44749400 1.13477800

C 1.86300600 1.85343200 0.00024100

O 1.44705500 1.44734100 -1.13455200

C -1.86277700 1.85367400 0.00010300

O -1.44778100 1.44665500 -1.13466500

C -1.86300500 -1.85344500 0.00033200

O -1.44798700 -1.44637800 1.13504500

Rh -0.00002500 0.00000000 -1.19201300

O -1.44709800 -1.44734900 -1.13447900

O 1.44801100 1.44638200 1.13497200

O -1.44689000 1.44748700 1.13486000

O 1.44773200 -1.44664900 -1.13474700

C 2.96598000 -2.88203500 0.00025200

H 2.90448800 -3.50145000 0.89618600

H 2.91283500 -3.49345000 -0.90163300

H 3.92905200 -2.36030700 0.00747000

C -2.96611100 -2.88192500 -0.00084000

H -2.93066400 -3.47622100 0.91314200

H -3.92878700 -2.36081000 -0.03871300

H -2.88701600 -3.51816600 -0.88365000

C -2.96592700 2.88210600 0.00027000

H -3.92905000 2.36043900 0.00347800

H -2.90667400 3.49929000 0.89787700

H -2.91041900 3.49577600 -0.89995800

C 2.96615700 2.88186300 -0.00086000

H 2.92870800 3.47815100 0.91175900

H 3.92889200 2.36061300 -0.03523000

H 2.88907300 3.51617200 -0.88522100

OAc-cb

0 1

Rh -3.06406400 -1.11360300 0.46214300

O -3.50973000 0.74130500 1.23904500

C -2.64330800 1.66100700 1.18907900

O -1.48172100 1.58512300 0.65376200

Rh -0.85429600 -0.21262700 -0.14129400

O -0.25558600 -0.80063000 1.72903000

C -1.06981400 -1.38995500 2.51784700

O -2.28344600 -1.66494600 2.27707000

O -1.67392100 0.28874100 -1.96847000

C -2.91470000 0.04891300 -2.19172800

O -3.72340800 -0.50627600 -1.39571700

O -0.39535200 -2.09361400 -0.84830400

C -1.27643300 -3.02172400 -0.78421000

O -2.45206500 -2.91148200 -0.33095500

C 0.94429600 0.49263700 -0.58241700

C 1.88960400 -0.11806100 -1.48538300

C 1.45388400 -0.64263000 -2.65816300

O 3.22052600 -0.01438000 -1.19316900

H 2.17273500 -0.91050600 -3.42554900

H 0.39548600 -0.74580100 -2.85335300

Si 3.99445900 -1.26887300 -0.30922100

C 4.44386300 -2.60348600 -1.56163100

H 4.89682000 -3.47272500 -1.07248000

H 5.14596300 -2.23409700 -2.31600000

H 3.54198200 -2.94717800 -2.08060800

C 5.54056000 -0.45472400 0.43965400

C 2.78723800 -1.94142400 0.95712600

H 3.26564000 -2.69909000 1.58845000

H 1.92689700 -2.40135000 0.45882600

H 2.40044300 -1.13662200 1.58754600

C 6.48200800 -1.55650200 0.97356300

H 7.37025400 -1.10350900 1.43440600

H 6.83146900 -2.22406400 0.17785700

H 5.99820100 -2.17145100 1.74228200

C 5.14434700 0.48007800 1.60145500

H 4.66381200 -0.07086900 2.41700700

H 4.44306300 1.25595000 1.28474100

H 6.04000400 0.96698500 2.01216100

C 6.26940500 0.36184200 -0.64805500

H 7.16724400 0.83630900 -0.22854900

H 5.62709500 1.15200900 -1.04953900

H 6.59464700 -0.26614500 -1.48632800

N 1.29087500 2.88804500 -0.22951500

C 0.62136500 3.19846000 -1.49412100

H 1.35046700 3.67852600 -2.16350300

H 0.29852500 2.26710400 -1.96601900

C -0.58342200 4.12214500 -1.26975000

H -1.35285400 3.54488200 -0.75028900

H -0.98683800 4.42259200 -2.24385400

C -0.19631800 5.35306200 -0.43581900

H -1.08181200 5.96542300 -0.23025800

H 0.49569300 5.98411000 -1.01209300

C 0.48164000 4.92892400 0.87603400

H -0.22914200 4.37163300 1.49740700

H 0.80681500 5.80563600 1.44787600

C 1.69018200 4.02846400 0.59420200

H 2.13039800 3.63528800 1.51142300

H 2.46511900 4.59340500 0.05571300

C 1.45439300 1.62910300 0.22930200

O 1.98367800 1.31428200 1.30239000

C -0.50137800 -1.77950300 3.86115300

H 0.38937200 -2.39548900 3.71096000

H -0.18732400 -0.87564900 4.39112400

H -1.24349800 -2.31989900 4.44873600

C -0.83525000 -4.37531900 -1.29016400

H -0.35975900 -4.26836700 -2.26830900

H -0.08741100 -4.78272300 -0.60238100

H -1.68437400 -5.05610100 -1.34975000

C -3.43694200 0.50278200 -3.53480700

H -3.48379900 1.59651400 -3.54567400

H -2.74884300 0.19362200 -4.32540800

H -4.43219200 0.09567500 -3.71306300

C -2.98601200 2.98983400 1.82330200

H -2.29646500 3.17707300 2.65188900

H -2.85096500 3.79482300 1.09569800

H -4.01224900 2.98383000 2.19013500

I-int1

0 1

Rh -2.56243800 -0.03123000 -1.49598800

C -3.89224300 -0.52152800 1.03745500

N -2.67828900 -0.63668800 1.55046800

O -4.13358200 -0.38970000 -0.21546000

C -5.13837300 -0.48585900 1.91091000

H -5.97518800 -0.72113000 1.24980300

H -5.09569400 -1.25884500 2.68808500

C -5.34926800 0.89549700 2.56048800

H -6.39501000 0.98411400 2.87896600

H -5.19125400 1.66849700 1.79808700

C -4.43948300 1.14328100 3.77024200

H -4.72797100 0.44376400 4.56841900

H -4.62673200 2.15068800 4.16411200

C -2.93784500 0.97380300 3.50146300

H -2.57619300 1.70927900 2.77732100

H -2.40194500 1.16823600 4.44077100

C -2.51158500 -0.41702900 2.99837300

H -3.02467200 -1.19948700 3.57803400

H -1.45299100 -0.53096000 3.21285200

C -1.77779200 2.29935200 0.10617800

N -2.49270300 1.92466600 -0.92734100

O -1.03075400 1.49039200 0.77851700

C -1.79518500 3.73096100 0.62423200

H -1.06315100 3.76684800 1.43287100

H -1.44649900 4.41578100 -0.16062200

C -3.17112600 4.19425300 1.14670700

H -3.01625000 5.07588300 1.78073300

H -3.58631900 3.41643800 1.79812200

C -4.18734300 4.55328600 0.05363900

H -5.10991600 4.90758900 0.53035200

H -3.79377300 5.40378300 -0.52254400

C -4.52268900 3.41928800 -0.92333700

H -5.25459900 3.79160600 -1.65195300

H -4.99119600 2.57617400 -0.40171600

C -3.30055700 2.88254100 -1.68393300

H -2.68263400 3.72289800 -2.03842800

H -3.64195200 2.35962300 -2.58301600

C 0.28435800 0.06497200 -2.26409600

N -0.94024100 0.26031800 -2.69956100

O 0.55470200 -0.25860000 -1.04186800

C 1.48777800 0.15921400 -3.18849600

H 1.51604600 1.15271700 -3.65615600

H 2.37109900 0.08055500 -2.55630500

C 1.50312700 -0.92470500 -4.28732400

H 1.26467500 -1.89645900 -3.83886600

H 2.52723200 -1.00806000 -4.67192700

C 0.56136300 -0.65114600 -5.46816300

H 0.67075400 -1.45464700 -6.20772500

H 0.89144900 0.27181600 -5.96748900

C -0.92119000 -0.49844000 -5.10176400

H -1.48797800 -0.29518900 -6.01989300

H -1.32076600 -1.42778200 -4.67898900

C -1.19679400 0.62808600 -4.09379800

H -2.25348400 0.90774300 -4.15370100

H -0.62877900 1.52944100 -4.37248500

C -1.82555300 -2.82807300 -1.46735700

N -1.01562700 -2.51590000 -0.46990000

O -2.65591600 -2.01087900 -2.00401100

C -1.81223600 -4.19892900 -2.12958800

H -1.77308100 -5.00067200 -1.38234800

H -2.76198200 -4.28312800 -2.66192300

C -0.63676000 -4.34623800 -3.11602200

H -0.56866900 -3.43244500 -3.71886900

H -0.85573800 -5.16319400 -3.81415600

C 0.70083500 -4.63029000 -2.42247500

H 1.49572300 -4.69474100 -3.17709800

H 0.64125300 -5.62383700 -1.95421300

C 1.10425200 -3.61638800 -1.34307000

H 2.05222100 -3.95668800 -0.90578700

H 1.29309500 -2.62601900 -1.76902700

C 0.09868200 -3.43877900 -0.19139500

H 0.64366700 -3.03513800 0.65073800

H -0.27665600 -4.41949300 0.13692600

Rh -1.00199600 -0.55364700 0.29848100

O 1.82844800 -1.00552700 1.48209700

C 0.45873400 -2.21315600 2.80767300

C -0.70825300 -3.00728200 2.84581100

C 1.53499200 -2.56438700 3.64786100

C -0.77981200 -4.10702300 3.69382600

H -1.52628200 -2.77372600 2.17515900

C 1.45851500 -3.68253000 4.47579200

H 2.41528600 -1.93375400 3.67423200

C 0.30055200 -4.46098400 4.50922700

H -1.68470800 -4.70873000 3.70039000

H 2.30316600 -3.93218300 5.11238600

H 0.23800400 -5.32724700 5.16073800

N 0.49441900 -1.06315600 1.99577000

C 2.42105000 0.11583600 1.22321000

C 2.12545500 1.41844900 1.76767000

C 1.11144400 1.67798500 2.62923000

O 3.08003800 2.35503400 1.47804900

H 1.09640400 2.63587100 3.13710400

H 0.32038900 0.96793000 2.79255400

Si 2.78518300 3.70315300 0.46509300

C 1.90474400 5.00950100 1.50376900

H 1.83684900 5.95958200 0.96173000

H 2.43346700 5.19527100 2.44388200

H 0.88384700 4.69754200 1.74267300

C 4.52211600 4.31099600 -0.02610700

C 1.70330500 3.19568100 -0.97902200

H 1.25463800 4.07592100 -1.45321100

H 0.89858400 2.54132800 -0.62874900

H 2.27521500 2.63500400 -1.71993500

C 4.36889900 5.44167100 -1.06710400

H 5.35581600 5.82798000 -1.35568900

H 3.79141200 6.28895000 -0.67785400

H 3.87630700 5.08943200 -1.98069700

C 5.35377600 3.16615000 -0.63877100

H 4.86750100 2.72037000 -1.51088400

H 5.51389100 2.35997500 0.08255200

H 6.33702000 3.54516200 -0.95167400

C 5.25885000 4.85433200 1.21657200

H 6.26930300 5.18794200 0.94270600

H 5.36299400 4.08513100 1.98985900

H 4.74183100 5.71213200 1.66100800

N 4.53004700 -0.98464700 0.50927300

C 4.60052600 -1.84878600 1.68880300

H 5.64171300 -1.83591400 2.03874500

H 3.99066200 -1.43253600 2.48804000

C 4.18202400 -3.27960000 1.32727400

H 3.12026300 -3.26765800 1.06279800

H 4.28815900 -3.92817800 2.20382600

C 5.02538900 -3.80385900 0.15161000

H 4.65720200 -4.78478500 -0.16861200

H 6.06112600 -3.95104200 0.48982200

C 5.02054000 -2.82188400 -1.03347400

H 4.01818100 -2.77393600 -1.47549700

H 5.70706700 -3.16138100 -1.81759400

C 5.41505800 -1.40731300 -0.58375400

H 5.32866000 -0.68115700 -1.39114300

H 6.44793400 -1.39324100 -0.21218100

C 3.57453300 -0.05652600 0.25076900

O 3.55142000 0.63442400 -0.76611000

I-ts1

0 1

Rh 3.26955500 -0.97357200 0.04347300

C 1.62978600 -3.12600500 1.09262700

N 0.61123000 -2.28833800 0.98189300

O 2.85315200 -2.80394300 0.88174100

C 1.43193300 -4.59147800 1.45273600

H 2.39802700 -4.94065900 1.82378500

H 0.70024000 -4.69810700 2.26315700

C 0.99233500 -5.43584700 0.24201100

H 1.18859000 -6.49393900 0.45364100

H 1.61797600 -5.17001800 -0.61961100

C -0.49050900 -5.26104600 -0.10393200

H -1.09151500 -5.66875100 0.72212900

H -0.73208500 -5.86834700 -0.98614600

C -0.93017100 -3.81205700 -0.34876700

H -0.41136000 -3.37719100 -1.20710900

H -1.99657200 -3.82458800 -0.60471400

C -0.74741600 -2.84840000 0.83577300

H -1.07570000 -3.33623300 1.76591400

H -1.41243500 -2.00417700 0.67326000

C 1.46960900 -1.50164500 -2.21537800

N 2.70642100 -1.61908000 -1.80250100

O 0.52942600 -0.98122400 -1.49386200

C 1.03273400 -2.00208800 -3.58612400

H 0.00209400 -1.67416500 -3.73318900

H 1.63562500 -1.51667200 -4.36542900

C 1.12242900 -3.53433700 -3.74931000

H 0.49009500 -3.82843500 -4.59585700

H 0.69501000 -4.01587800 -2.86236000

C 2.54242500 -4.06302500 -3.99293700

H 2.49801100 -5.15072800 -4.12921800

H 2.91072300 -3.64934000 -4.94345200

C 3.55896100 -3.72958900 -2.89279900

H 4.53015400 -4.15591700 -3.17537300

H 3.27302200 -4.19205600 -1.94075100

C 3.73877700 -2.22289700 -2.64912600

H 3.81396300 -1.69465900 -3.61294400

H 4.69196000 -2.05848000 -2.13778200

C 2.64665700 1.80843500 -0.72223900

N 3.59627400 0.90196100 -0.70271700

O 1.43291300 1.58158300 -0.33808300

C 2.91247800 3.24019000 -1.16651200

H 3.31063500 3.25159500 -2.19011100

H 1.94152300 3.73703300 -1.18985200

C 3.87077300 4.00566900 -0.22977700

H 3.57251300 3.82539000 0.80944900

H 3.73997300 5.08060800 -0.40587600

C 5.35456600 3.65931200 -0.41569900

H 5.95135500 4.27423400 0.26958300

H 5.65516100 3.95409900 -1.43216300

C 5.71350800 2.18072800 -0.21442900

H 6.79065400 2.05856300 -0.38764000

H 5.51846300 1.86363200 0.81661100

C 4.95437000 1.22586500 -1.14922600

H 5.49775100 0.27795600 -1.20609900

H 4.94540700 1.63344000 -2.17271800

C 2.94206200 0.28708200 2.61807700

N 1.67659700 0.46144700 2.27706300

O 3.79724300 -0.36399600 1.91762600

C 3.53935600 0.90689400 3.87448700

H 2.87534900 0.77458500 4.73692300

H 4.45878100 0.35149600 4.07173500

C 3.85678800 2.40311800 3.68173700

H 4.34763500 2.53354200 2.70911100

H 4.58494600 2.71440400 4.44056700

C 2.61954700 3.30353200 3.77858300

H 2.91458500 4.34626200 3.60142600

H 2.24333000 3.26328300 4.81125300

C 1.46989500 2.92716700 2.83460900

H 0.64188000 3.62746000 3.01016200

H 1.75815300 3.04100800 1.78478800

C 0.92219400 1.49830300 3.00597500

H -0.08709200 1.49183500 2.62206400

H 0.84176100 1.24555400 4.07327500

Rh 0.95290400 -0.30050400 0.44884700

O -1.41811900 1.43759500 0.07498100

C -1.88258700 0.26896600 1.97594700

C -1.51866600 -0.65905600 2.97997300

C -2.97900200 1.12681100 2.22775500

C -2.24134800 -0.73788000 4.16585700

H -0.62741900 -1.25757500 2.84312800

C -3.68981000 1.03214000 3.41861000

H -3.26108800 1.85895000 1.48389500

C -3.33752600 0.09768400 4.39805500

H -1.93322300 -1.45388100 4.92312500

H -4.52862500 1.70307200 3.58658400

H -3.89759100 0.03151600 5.32559400

N -1.06445600 0.37471600 0.84807600

C -2.18414300 1.14506800 -1.25691900

C -2.93117400 -0.17567100 -1.22022500

C -2.62788600 -1.21175100 -2.00785500

O -3.85538100 -0.18588000 -0.24966300

H -3.17264800 -2.14063800 -1.90737200

H -1.81070900 -1.17765200 -2.71039100

Si -5.54070900 -0.45373200 -0.28720700

C -6.09293600 -0.62342000 -2.07442400

H -7.18238800 -0.72085300 -2.13173700

H -5.64959700 -1.49335400 -2.56726700

H -5.79318800 0.27249600 -2.62469900

C -5.81796900 -2.05696000 0.70731600

C -6.32379200 1.02825700 0.55264200

H -7.41004000 0.91511900 0.63627400

H -6.10711800 1.92817200 -0.02889800

H -5.91387100 1.15637800 1.55822200

C -7.32051600 -2.40956000 0.69061300

H -7.50343500 -3.32475200 1.26995800

H -7.69141400 -2.58766600 -0.32577200

H -7.93154800 -1.61655900 1.13794700

C -5.34573500 -1.86513200 2.16300600

H -5.91119600 -1.08284800 2.67929700

H -4.28747300 -1.59383400 2.21535200

H -5.48185200 -2.79631700 2.73054400

C -5.01150000 -3.21260400 0.08136500

H -5.19626000 -4.14784000 0.62728200

H -3.93670900 -3.01282900 0.13299500

H -5.27925200 -3.39105400 -0.96733000

N -2.56282100 3.61677600 -1.34793100

C -1.14974900 3.97849100 -1.12056200

H -1.11935000 4.61192100 -0.22375700

H -0.54188800 3.10702700 -0.89023600

C -0.60787500 4.75676200 -2.32873000

H -0.57478500 4.08597900 -3.19727800

H 0.42336100 5.06136400 -2.12098700

C -1.47920000 5.98009700 -2.64672500

H -1.11884200 6.48414500 -3.55009600

H -1.39347000 6.70388900 -1.82393200

C -2.95032800 5.57194600 -2.80540100

H -3.07138600 4.94429300 -3.69756200

H -3.58838600 6.45282200 -2.93971600

C -3.43209100 4.78213700 -1.58250600

H -4.45285000 4.42511900 -1.70739100

H -3.39110000 5.41469300 -0.68531400

C -3.11230000 2.38174900 -1.47566500

O -4.29191600 2.18140100 -1.75324500

N -1.23792000 1.14719000 -2.30933200

N -0.21308100 1.15942700 -2.80102100

I-ts2

0 1

Rh -2.80348400 -0.18906600 -1.17701700

C -3.69156200 0.51066400 1.49931600

N -2.43036000 0.34628500 1.86537400

O -4.13953200 0.24481300 0.32771900

C -4.73864100 1.09165200 2.43993500

H -5.70716400 0.82104000 2.01430200

H -4.67048400 0.63600200 3.43551400

C -4.62265000 2.62408800 2.55851200

H -5.56887600 3.02404000 2.94241900

H -4.49435700 3.04419800 1.55333600

C -3.48251300 3.08187800 3.47695300

H -3.71902200 2.76932600 4.50454900

H -3.44754600 4.17883600 3.49133700

C -2.09549600 2.53373900 3.11437700

H -1.77257100 2.89259600 2.13216100

H -1.37372000 2.92221300 3.84583200

C -1.98306800 0.99819900 3.10782800

H -2.50444100 0.58174000 3.98340400

H -0.93600700 0.74530900 3.24289800

C -1.44238400 2.36616800 -0.71303600

N -2.38231200 1.78995900 -1.42051600

O -0.69049300 1.71978900 0.11586100

C -1.18306700 3.86404400 -0.78463000

H -0.28625900 4.05362000 -0.19335300

H -0.95165900 4.15284900 -1.81837900

C -2.35601700 4.71726700 -0.25924600

H -1.97583500 5.72094400 -0.03294200

H -2.70634100 4.30031000 0.69218400

C -3.53387800 4.85150800 -1.23468300

H -4.29486900 5.50187400 -0.78535500

H -3.17980000 5.37236400 -2.13653400

C -4.18653200 3.53009000 -1.66402700

H -5.00557900 3.75661900 -2.35894800

H -4.63012800 3.01157200 -0.80558100

C -3.21637500 2.55480300 -2.34928600

H -2.59519100 3.09437400 -3.08137900

H -3.79296800 1.82317800 -2.92431300

C -0.14984800 -0.85310000 -2.27996900

N -1.40975900 -0.65220500 -2.59881300

O 0.28834100 -0.79713900 -1.06784500

C 0.89738800 -1.21972900 -3.31862000

H 0.91654700 -0.45633000 -4.10805200

H 1.86353600 -1.17387800 -2.81575400

C 0.66358700 -2.60694500 -3.95093600

H 0.42357000 -3.32956800 -3.16088700

H 1.60472700 -2.94557400 -4.40157200

C -0.42940100 -2.63079600 -5.02905100

H -0.50179000 -3.64359000 -5.44540600

H -0.11144800 -1.98093500 -5.85767000

C -1.82070800 -2.17627500 -4.56496200

H -2.50442700 -2.22448800 -5.42280100

H -2.22004500 -2.85552400 -3.80254100

C -1.85955600 -0.75125600 -3.98915000

H -2.89385300 -0.39279200 -4.00951500

H -1.28762100 -0.06869600 -4.63725800

C -2.43597400 -2.87563100 -0.17909900

N -1.42118700 -2.39086000 0.51733100

O -3.24731500 -2.15768300 -0.86508000

C -2.71009400 -4.36857200 -0.29497300

H -2.62152900 -4.87044600 0.67602900

H -3.74726100 -4.45731500 -0.62482700

C -1.76855300 -5.03608300 -1.31708300

H -1.70538900 -4.39608700 -2.20556700

H -2.20994900 -5.98457200 -1.64605700

C -0.36752700 -5.30450200 -0.75684500

H 0.25861400 -5.75185600 -1.53994100

H -0.45180900 -6.06110600 0.03717000

C 0.34685600 -4.07683400 -0.17663000

H 1.31513400 -4.41264000 0.21394200

H 0.56263000 -3.33077800 -0.94773100

C -0.39492600 -3.35172300 0.96115000

H 0.34311000 -2.79848400 1.52540100

H -0.82190800 -4.08390200 1.66305300

Rh -1.01610400 -0.32123600 0.46874200

O 1.90730200 -0.95349800 1.28904100

C 0.77727500 -1.18515700 3.22763400

C -0.43352800 -1.70436800 3.71561700

C 1.95582000 -1.37060900 3.96970700

C -0.45483000 -2.39426400 4.92583400

H -1.33219900 -1.59889100 3.11927900

C 1.92643800 -2.07148000 5.17245600

H 2.88038600 -0.93220000 3.61114400

C 0.72019100 -2.58449700 5.65776400

H -1.39460900 -2.80031600 5.28899800

H 2.84354800 -2.20504500 5.73911100

H 0.69674900 -3.12650600 6.59848800

N 0.79314000 -0.45964500 1.99918600

C 2.56551200 0.00481100 0.62985400

C 2.48980900 1.31075300 1.06802000

C 1.54999900 1.71314200 2.04380700

O 3.54137400 2.12934600 0.74092500

H 1.93507600 2.39707300 2.80406700

H 0.49562800 1.75343200 1.84991200

Si 3.30304000 3.45124100 -0.32159800

C 2.54281200 4.84872300 0.69151700

H 2.36737000 5.74127900 0.08055300

H 3.18756700 5.13070200 1.52958300

H 1.57777200 4.53400300 1.10391400

C 5.06539200 3.85679000 -0.91172600

C 2.15346000 2.94148200 -1.71134100

H 1.80175600 3.81738800 -2.26781000

H 1.28621000 2.41762100 -1.29754000

H 2.64961700 2.24794300 -2.39254300

C 4.98640200 4.89022600 -2.05558400

H 5.99496400 5.14792100 -2.40665900

H 4.50697700 5.82390100 -1.73582000

H 4.42933500 4.50311200 -2.91614400

C 5.74145000 2.56741900 -1.42600800

H 5.16016800 2.07750100 -2.21320500

H 5.86566700 1.83784500 -0.62043700

H 6.73598000 2.80056700 -1.83153400

C 5.90156700 4.43688000 0.24760700

H 6.93170100 4.62735200 -0.08409200

H 5.95067400 3.74348700 1.09460500

H 5.49602300 5.38769200 0.61163500

N 4.27406700 -1.56876600 -0.16206100

C 4.53370700 -2.11886200 1.16901900

H 5.62539100 -2.17477200 1.29059200

H 4.16034800 -1.43475100 1.92826600

C 3.91981400 -3.51497700 1.31063900

H 2.83150300 -3.40877500 1.29147800

H 4.18976700 -3.93888000 2.28480300

C 4.39457200 -4.43007700 0.16869300

H 3.87178700 -5.39219300 0.21185600

H 5.46406100 -4.64648500 0.30380600

C 4.18800000 -3.77420700 -1.20770500

H 3.11554900 -3.67378000 -1.41463800

H 4.61792300 -4.39896400 -1.99934100

C 4.82007000 -2.37397900 -1.25679000

H 4.60882100 -1.85846900 -2.19332400

H 5.91095900 -2.43982000 -1.14091900

C 3.49123400 -0.48994500 -0.44740100

O 3.46130800 0.04770800 -1.55471800

II-int1

0 1

C -4.69389100 1.01054300 0.97228700

C -5.71011900 1.87891500 0.76130800

O -4.89654900 -0.18779200 1.61474800

H -5.53915400 2.82941100 0.28418600

H -6.70782700 1.60951800 1.08702700

Si -5.66880900 -1.56826500 0.98087000

C -7.39059500 -1.08483300 0.38820700

H -7.96418600 -0.62841400 1.20189900

H -7.94334500 -1.96558200 0.04476400

H -7.35022400 -0.36943600 -0.43868900

C -4.66318700 -2.35050600 -0.44174200

C -5.78457200 -2.70828200 2.46857300

H -6.28149700 -3.64808000 2.20539600

H -6.36524400 -2.23457600 3.26689000

H -4.79745600 -2.95116700 2.87088900

C -5.49154200 -3.52934100 -1.00534100

H -4.92461100 -4.03861300 -1.79603000

H -6.43709300 -3.19654100 -1.44715800

H -5.72111900 -4.27986800 -0.23937600

C -3.30863300 -2.89219500 0.06435700

H -3.43770800 -3.65222700 0.84349000

H -2.66773100 -2.10236600 0.46067400

H -2.75640000 -3.35923500 -0.76121100

C -4.41473600 -1.33164800 -1.57584500

H -3.97363200 -1.83799100 -2.44413500

H -3.71489200 -0.54848900 -1.27445600

H -5.33866900 -0.85112900 -1.91710500

N -2.06354500 0.01962300 2.31929000

C -2.69298400 0.72911600 3.44138000

H -1.89259300 1.27971900 3.95940300

H -3.40074900 1.46625600 3.06400100

C -3.36976800 -0.23648100 4.41887600

H -4.23603100 -0.67987100 3.92139900

H -3.73782700 0.33638800 5.27805100

C -2.39796800 -1.33473700 4.87014500

H -2.91253000 -2.05748700 5.51345900

H -1.59455900 -0.88743200 5.47285200

C -1.77936400 -2.03066700 3.65120600

H -2.55413000 -2.57175600 3.09435000

H -1.03122600 -2.76919500 3.96138700

C -1.10229400 -1.02347300 2.71788800

H -0.70981300 -1.50172600 1.82323300

H -0.25114900 -0.54207900 3.20864100

C -2.14038200 0.41407100 1.03048600

O -1.29995100 0.10123000 0.15836300

Rh 3.44454100 -0.28131400 -0.31600800

C 2.59110900 2.46172100 -0.69174200

N 1.37482200 1.99125800 -0.50420200

O 3.64303500 1.72221700 -0.74850200

C 2.86292000 3.95584400 -0.79504800

H 3.88914700 4.05590400 -1.15483800

H 2.20263600 4.42080300 -1.53773900

C 2.70498200 4.67046500 0.56331300

H 3.23068800 5.63209200 0.51647600

H 3.21837600 4.07714800 1.32986300

C 1.24854600 4.93096100 0.97835000

H 0.80900300 5.63919600 0.26073800

H 1.24086500 5.43800200 1.95200000

C 0.34686600 3.68888800 1.03823900

H 0.68727100 2.98566800 1.80736100

H -0.66525000 4.01174000 1.31958700

C 0.26211900 2.92009900 -0.29077500

H 0.16777300 3.62494500 -1.12921300

H -0.64283800 2.32229500 -0.29987100

C 2.54710900 0.49097000 2.37931300

N 3.60718800 0.17255500 1.67214000

O 1.34864800 0.47076900 1.90581100

C 2.65421800 0.96113000 3.82243800

H 1.63628500 0.98101600 4.21895600

H 3.22608200 0.23756600 4.41823800

C 3.29577100 2.35811500 3.94580700

H 3.04541100 2.77270600 4.92997400

H 2.83386100 3.02145800 3.20473800

C 4.82392700 2.36567300 3.78895600

H 5.18720500 3.39478200 3.90329100

H 5.25759900 1.79392200 4.62285300

C 5.36200100 1.78276800 2.47324100

H 6.45805700 1.84395600 2.49289000

H 5.02322300 2.36992800 1.61171000

C 4.95393100 0.31974400 2.22634000

H 5.06888000 -0.26520600 3.15262000

H 5.64232400 -0.12549900 1.50075300

C 1.96121000 -2.76229900 0.29079600

N 3.16267900 -2.26863700 0.08947200

O 0.89883600 -2.03441000 0.34429100

C 1.71658100 -4.25886100 0.41449300

H 2.36748200 -4.69443900 1.18386900

H 0.68361000 -4.37726300 0.74930600

C 1.92988900 -4.99627400 -0.92395900

H 1.42885400 -4.43087600 -1.71891300

H 1.42486600 -5.96865900 -0.87212300

C 3.40379000 -5.23292000 -1.28801100

H 3.45112200 -5.77282200 -2.24222500

H 3.83992300 -5.90536800 -0.53439100

C 4.27995000 -3.97381600 -1.37596100

H 5.30259000 -4.28306100 -1.62924500

H 3.93834900 -3.30679400 -2.17592700

C 4.31879800 -3.15147900 -0.07696300

H 5.20637300 -2.51014000 -0.08574800

H 4.43696500 -3.81922800 0.79098700

C 2.00249500 -0.76402100 -2.79494700

N 0.91673200 -0.49902300 -2.09668500

O 3.19604800 -0.69131000 -2.31783200

C 1.93367300 -1.24185500 -4.23808300

H 1.30655500 -0.56953900 -4.83759600

H 2.95171000 -1.18015300 -4.62816400

C 1.40510500 -2.68620200 -4.35607100

H 1.90906600 -3.30690900 -3.60485800

H 1.69775100 -3.08811800 -5.33380000

C -0.11829600 -2.80771400 -4.21174400

H -0.40442000 -3.86282900 -4.31201900

H -0.58614200 -2.28240200 -5.05763000

C -0.70525800 -2.24117300 -2.91133300

H -1.79338400 -2.37778800 -2.93888500

H -0.34021000 -2.79118700 -2.03604300

C -0.40811100 -0.74982100 -2.67654500

H -1.13781500 -0.35763100 -1.97111600

H -0.53786000 -0.18961700 -3.61587000

Rh 1.05468400 -0.00386400 -0.10118900

C -3.31579500 1.22344200 0.57808600

O -2.95085300 2.00451300 -0.41432700

C -3.15143200 3.28585400 -2.21124200

C -3.76228100 4.38908000 -2.84520000

C -2.00512100 2.70100600 -2.79667300

C -3.22205600 4.91160200 -4.01438600

H -4.64953200 4.82190600 -2.39364500

C -1.47294300 3.24186000 -3.96135000

H -1.54897700 1.83299200 -2.34028900

C -2.07126300 4.34760900 -4.57650400

H -3.69674800 5.76555600 -4.48902800

H -0.58718400 2.78810700 -4.39741000

H -1.65090700 4.75889800 -5.48916400

N -3.78318200 2.84544900 -1.05583400

II-ts1

0 1

C -2.44651000 0.37864100 0.95274600

C -2.05844500 1.06586400 2.04404800

O -3.76817000 0.32492200 0.57062100

H -2.78682300 1.59137200 2.65257300

H -1.01479900 1.11013000 2.31199900

Si -4.97066900 1.47674600 0.28709000

C -4.37111500 3.19519800 0.77108300

H -4.43442200 3.35616700 1.85223900

H -4.97561800 3.96677400 0.28206300

H -3.32893800 3.31951100 0.46818600

C -5.44737300 1.38241700 -1.55870400

C -6.42236700 0.95521400 1.36943900

H -7.24872300 1.67105700 1.30266400

H -6.11156400 0.89849300 2.41823300

H -6.80745500 -0.02882700 1.08368800

C -6.70489100 2.24904400 -1.78969200

H -7.00480200 2.20207900 -2.84533700

H -6.52910400 3.30511500 -1.55174300

H -7.55878200 1.90719600 -1.19370300

C -5.75269100 -0.08065000 -1.94185700

H -6.55590600 -0.51153500 -1.33197900

H -4.86175100 -0.70436200 -1.83368300

H -6.07346800 -0.13821800 -2.99103300

C -4.30324200 1.88730100 -2.46303700

H -4.64504400 1.93378600 -3.50620900

H -3.44583000 1.21119300 -2.43078100

H -3.96380200 2.89240700 -2.18578300

N -2.93317900 -2.36059300 -0.33105700

C -3.20075700 -2.60940900 1.08385500

H -2.64114400 -3.50683600 1.39722900

H -2.84435100 -1.78047900 1.68851600

C -4.69837100 -2.83528000 1.32192400

H -5.22235000 -1.90377700 1.08418500

H -4.86466400 -3.04877400 2.38459800

C -5.22185800 -3.98023900 0.44490400

H -6.30243000 -4.10679700 0.57745200

H -4.75092900 -4.92179700 0.76307700

C -4.88495400 -3.71839200 -1.03007700

H -5.45562500 -2.85547100 -1.38918200

H -5.16678300 -4.57667000 -1.65154500

C -3.38864700 -3.43717300 -1.21538200

H -3.15707600 -3.14444400 -2.23849400

H -2.81289400 -4.34700100 -0.97806500

C -2.21307200 -1.31028500 -0.87535800

O -2.08398200 -1.20256500 -2.09403100

Rh 3.07396400 -0.01332900 0.54838500

C 2.39658000 -2.82789700 0.23547000

N 1.14142700 -2.44550900 0.07115800

O 3.34661900 -2.05158900 0.60937600

C 2.84019400 -4.25542700 -0.06860700

H 3.86812300 -4.33077800 0.29178300

H 2.24056300 -4.98417500 0.49108700

C 2.78703100 -4.58109100 -1.57741900

H 3.43346400 -5.44654900 -1.76792500

H 3.22734300 -3.74274000 -2.12820300

C 1.38493200 -4.88889900 -2.12435000

H 1.03263900 -5.82149300 -1.65888100

H 1.46043800 -5.09541100 -3.19971200

C 0.32760500 -3.80308600 -1.87953600

H 0.55222800 -2.87830200 -2.42107800

H -0.63604600 -4.15951200 -2.26239700

C 0.17307600 -3.44204500 -0.39402700

H 0.23111300 -4.35449300 0.21857500

H -0.81361700 -3.03618300 -0.22047000

C 2.17194300 -0.16273000 -2.20860600

N 3.25816800 -0.09460500 -1.47326700

O 0.98051600 -0.19123400 -1.72179400

C 2.22720200 -0.22630200 -3.72964000

H 1.19396500 -0.15673900 -4.07625500

H 2.77267800 0.64414600 -4.12175300

C 2.87217200 -1.52056700 -4.26357900

H 2.57635100 -1.64794000 -5.31194800

H 2.44960800 -2.37359400 -3.72038500

C 4.40511400 -1.54328700 -4.18530500

H 4.77012700 -2.48409200 -4.61656900

H 4.79478000 -0.74080000 -4.82917600

C 5.00330100 -1.36741000 -2.78217300

H 6.09772100 -1.38316500 -2.86977900

H 4.72705800 -2.20076900 -2.12687200

C 4.59113200 -0.06445500 -2.07863700

H 4.68492500 0.78228000 -2.77788900

H 5.29603100 0.13228800 -1.26505600

C 1.51878200 2.40472900 0.76122700

N 2.73282300 1.99172400 0.49936100

O 0.49815400 1.61522000 0.75494600

C 1.21324500 3.82971600 1.19050400

H 1.57577900 4.54516100 0.44156000

H 0.12636900 3.91854500 1.22579900

C 1.82251600 4.15284800 2.57147200

H 1.63075900 3.30938300 3.24732300

H 1.29374400 5.01797600 2.99023900

C 3.32581800 4.46742800 2.54053000

H 3.65300000 4.73771900 3.55238600

H 3.47916600 5.36254000 1.91903900

C 4.22090000 3.34035200 2.00451500

H 5.26407400 3.68205200 2.02714100

H 4.15126800 2.45495500 2.64763300

C 3.87180600 2.90808500 0.56974500

H 4.72292900 2.38232400 0.12586000

H 3.69820400 3.79570400 -0.05867500

C 1.74819500 -0.50130700 3.09189500

N 0.68413200 -0.85565600 2.38273300

O 2.76712900 0.09505000 2.59722800

C 1.85260100 -0.77886700 4.58615300

H 2.07144400 -1.84782200 4.72422900

H 2.74170600 -0.23762300 4.91717700

C 0.63564600 -0.38456400 5.44166500

H 0.27248100 0.60182400 5.12338800

H 0.97121700 -0.27140500 6.47958700

C -0.50956500 -1.40263400 5.40465000

H -1.27888200 -1.11612000 6.13283500

H -0.11851400 -2.37578200 5.73632500

C -1.15468900 -1.56730500 4.02615400

H -1.91094400 -2.36277400 4.08178700

H -1.68420600 -0.65175200 3.74591600

C -0.17803500 -1.93331800 2.89866700

H -0.74693100 -2.29268700 2.04643300

H 0.44059000 -2.78573600 3.21939100

Rh 0.64224800 -0.43405100 0.34461000

C -1.51929700 -0.36477000 0.09004400

O -1.31737300 1.11276600 -1.25900900

C -0.71806500 3.21656900 -1.61121500

C -1.00976700 4.56258000 -1.34721900

C 0.24910100 2.84980400 -2.56371200

C -0.33514400 5.55557200 -2.05173000

H -1.75613700 4.80387000 -0.59743800

C 0.92639400 3.85136000 -3.24699600

H 0.45394600 1.79540100 -2.70215200

C 0.63552800 5.19997300 -2.99473900

H -0.55426500 6.60181500 -1.86296000

H 1.68772200 3.58829100 -3.97537100

H 1.17176300 5.97615500 -3.53325200

N -1.41848100 2.26477800 -0.83432400

II-ts2

0 1

C -4.48698900 0.77357600 1.28325200

C -5.54329300 1.58723100 0.81782600

O -4.71170500 -0.47082200 1.82269700

H -5.66890200 2.60643800 1.13588800

H -6.46074900 1.07996200 0.52180800

Si -5.35178800 -1.88476100 1.12776400

C -7.12978600 -1.57103800 0.57768200

H -7.68537200 -1.02857100 1.34963200

H -7.64231000 -2.52397800 0.40629400

H -7.18779500 -0.99729600 -0.35290200

C -4.30689200 -2.50353700 -0.34498800

C -5.34181600 -3.09632400 2.56239500

H -5.74443400 -4.06735300 2.25451700

H -5.96079900 -2.72218500 3.38434900

H -4.33185600 -3.25803800 2.94852100

C -5.08711300 -3.65430800 -1.02341300

H -4.49770000 -4.06172000 -1.85551600

H -6.04477000 -3.31868700 -1.43599800

H -5.28692000 -4.48355100 -0.33371400

C -2.93967800 -3.04185300 0.12882700

H -3.05093700 -3.85049100 0.86012700

H -2.31726000 -2.26015400 0.56872700

H -2.37755300 -3.44656300 -0.72181000

C -4.08169700 -1.37447300 -1.37510600

H -3.61926700 -1.78052800 -2.28411800

H -3.40865400 -0.60738800 -0.98865800

H -5.01583000 -0.88818700 -1.68001900

N -1.74559500 -0.14384500 2.52996300

C -2.35357800 0.43389800 3.73596700

H -1.54908600 0.94750200 4.28377100

H -3.08944100 1.18383500 3.45046400

C -2.97729800 -0.64838900 4.62277300

H -3.83595700 -1.07281900 4.09508600

H -3.35160300 -0.17964100 5.54038600

C -1.95441500 -1.74648700 4.94749500

H -2.42743800 -2.55202600 5.52062500

H -1.16063200 -1.32801500 5.58274000

C -1.32865200 -2.29074900 3.65616100

H -2.08808400 -2.81857200 3.06660200

H -0.53744300 -3.01516600 3.88104500

C -0.72787700 -1.17113500 2.80032400

H -0.34444800 -1.55079300 1.85728700

H 0.11452800 -0.69096800 3.30902900

C -1.94721500 0.31845200 1.28138400

O -1.22253700 0.04945000 0.29897800

Rh 3.52338500 -0.00056100 -0.37957100

C 2.41173400 2.61787900 -0.94360400

N 1.24667100 2.03641700 -0.75413600

O 3.54064500 2.00593300 -0.84137300

C 2.52297700 4.10394200 -1.25017900

H 3.57393000 4.28944000 -1.48176900

H 1.94034200 4.35294900 -2.14661100

C 2.06985800 4.99319700 -0.07354100

H 2.50100100 5.99307500 -0.20698600

H 2.49939600 4.59380100 0.85432300

C 0.54687100 5.13172100 0.06584900

H 0.16311300 5.63126700 -0.83602200

H 0.32415500 5.80341600 0.90499800

C -0.21886200 3.81349700 0.24788700

H 0.06599600 3.32549700 1.18902900

H -1.29123300 4.03418200 0.30833300

C 0.00605400 2.80294100 -0.88841200

H -0.03419300 3.31781600 -1.86038900

H -0.80640200 2.08264200 -0.88837200

C 2.58767600 0.85164300 2.27434400

N 3.66283800 0.52345900 1.59263100

O 1.39094000 0.71743900 1.81792600

C 2.67590500 1.48807300 3.65332800

H 1.66322700 1.48516500 4.06362300

H 3.30576000 0.88553700 4.32066700

C 3.21883800 2.93094100 3.58618100

H 2.93821900 3.45591000 4.50757400

H 2.71418700 3.45427300 2.76440800

C 4.74308500 3.02176100 3.41776000

H 5.03533100 4.07934800 3.40067200

H 5.21438400 2.58887100 4.31282200

C 5.31804700 2.31926700 2.17826500

H 6.40842000 2.44896300 2.18368000

H 4.94141600 2.77576300 1.25545100

C 4.99986800 0.81501000 2.11370300

H 5.15027900 0.35317100 3.10244200

H 5.71289100 0.32418800 1.44278500

C 2.27979200 -2.57549300 0.34012900

N 3.42565900 -1.99369000 0.06688400

O 1.16387000 -1.93553300 0.41141300

C 2.16859300 -4.08036400 0.53431600

H 2.87854600 -4.42356200 1.29828300

H 1.16100500 -4.27130200 0.91042300

C 2.40319400 -4.85843600 -0.77742800

H 1.83682600 -4.37216200 -1.58110700

H 1.98059100 -5.86474100 -0.66752300

C 3.88047500 -4.99239100 -1.17626300

H 3.94677100 -5.57479800 -2.10397700

H 4.39239100 -5.58667100 -0.40486500

C 4.64440900 -3.67078600 -1.35388200

H 5.68247400 -3.90619000 -1.62309500

H 4.22591900 -3.07852400 -2.17575600

C 4.64901200 -2.78039100 -0.10011200

H 5.47475100 -2.06506200 -0.17432100

H 4.85427500 -3.38898200 0.79474500

C 2.08068000 -0.69398900 -2.81918000

N 0.98940300 -0.53042600 -2.09900400

O 3.26913900 -0.47497600 -2.37569700

C 2.02636900 -1.22003500 -4.24516300

H 1.32922200 -0.62491900 -4.84914600

H 3.02622700 -1.07978600 -4.66097400

C 1.62681300 -2.70805500 -4.30682600

H 2.19389600 -3.25640800 -3.54440800

H 1.93929500 -3.11620200 -5.27578900

C 0.12209100 -2.95994200 -4.13293700

H -0.07029000 -4.03849700 -4.20174800

H -0.40296900 -2.50128000 -4.98409100

C -0.49732500 -2.41571400 -2.83753900

H -1.57000200 -2.64546300 -2.85164500

H -0.07859000 -2.91425600 -1.95527000

C -0.32349900 -0.90085500 -2.63549200

H -1.06420700 -0.55194100 -1.91650200

H -0.52500100 -0.37166000 -3.58083700

Rh 1.11955900 0.08127500 -0.14760300

C -3.17437600 1.13502200 1.01417800

O -2.95625500 2.02740400 0.03954500

C -4.02921700 2.91091000 -1.72176100

C -4.83840300 3.92715200 -2.26583400

C -3.25697000 2.10272400 -2.58072800

C -4.86510100 4.13710400 -3.63972500

H -5.42630600 4.54211800 -1.59125500

C -3.27637300 2.33906400 -3.95255800

H -2.65054400 1.30683800 -2.16804900

C -4.07946600 3.34940700 -4.48965000

H -5.49017800 4.92410400 -4.05166600

H -2.67021000 1.71931800 -4.60756900

H -4.09738000 3.52021600 -5.56188500

N -4.06825500 2.78449400 -0.32417100

III-int1

0 1

C -2.32765100 0.54435700 0.43506200

C -1.81401600 1.81019400 1.05769600

O -3.70186100 0.54206100 0.22967200

H -2.58182800 2.21230100 1.73461300

H -0.90891500 1.61242500 1.61481300

Si -4.76560000 1.02348500 -0.99522400

C -5.19197100 2.85954000 -0.82796100

H -5.26337600 3.12594700 0.23302300

H -6.17243600 3.04664900 -1.28086400

H -4.46467900 3.52407600 -1.29558600

C -4.20849200 0.55369900 -2.77164800

C -6.35994500 0.11136200 -0.55961300

H -7.14293900 0.35168000 -1.28814900

H -6.72334900 0.41326100 0.42871900

H -6.22848800 -0.97280000 -0.55682100

C -4.98149900 1.46343200 -3.75527800

H -4.73831600 1.18471700 -4.78983400

H -4.72138700 2.52002000 -3.62994400

H -6.06961600 1.36974900 -3.64415800

C -4.57940200 -0.91690300 -3.06872000

H -5.65735800 -1.09396600 -2.97989600

H -4.04750200 -1.60992800 -2.41244100

H -4.29292600 -1.16709500 -4.09997000

C -2.69263500 0.73898100 -2.99876300

H -2.45264500 0.50823400 -4.04746300

H -2.09849200 0.07099400 -2.37144700

H -2.37357300 1.76691800 -2.80500400

N -2.99715800 -2.52516200 0.76462400

C -3.35021000 -2.09369300 2.11284500

H -2.97128800 -2.84071000 2.83131800

H -2.85862100 -1.14620800 2.32580400

C -4.87156500 -1.95599200 2.26607100

H -5.19300900 -1.11762400 1.64306700

H -5.11060500 -1.70719400 3.30765400

C -5.59255800 -3.24464200 1.84033200

H -6.67892200 -3.10177300 1.87218700

H -5.35830300 -4.04415700 2.55882900

C -5.14674500 -3.69070300 0.43915700

H -5.48794200 -2.97109300 -0.31382000

H -5.58940500 -4.66111400 0.18383500

C -3.61846700 -3.78436500 0.35952100

H -3.27781700 -4.01161400 -0.65000500

H -3.26605600 -4.58011000 1.03548500

C -2.24436900 -1.80683100 -0.15122600

O -2.10581800 -2.22158800 -1.30554200

Rh 2.98853700 -0.01430300 -0.06772500

C 2.40021800 -2.51937700 1.32349400

N 1.11628600 -2.24291200 1.17296700

O 3.35474300 -1.70963400 1.02592300

C 2.87003100 -3.87890200 1.82495200

H 3.94664200 -3.78472400 1.98005800

H 2.41654200 -4.11689500 2.79544600

C 2.57710800 -5.01470600 0.82009000

H 3.23762500 -5.85901600 1.05159900

H 2.85417000 -4.67451500 -0.18424500

C 1.12454400 -5.51318200 0.82354100

H 0.92128900 -5.96987800 1.80356800

H 1.02263200 -6.31830500 0.08498200

C 0.05941700 -4.43947000 0.56300600

H 0.12866000 -4.03127800 -0.45103600

H -0.93063800 -4.90296100 0.64246600

C 0.12891300 -3.26184800 1.54729300

H 0.32795600 -3.63309700 2.56380900

H -0.83491100 -2.77084300 1.58803200

C 1.68707100 -1.59248800 -2.13450100

N 2.87345500 -1.18198100 -1.72039300

O 0.59032200 -1.31741500 -1.53049000

C 1.51731600 -2.47614100 -3.35945300

H 0.44249100 -2.53076200 -3.54127700

H 1.98604600 -2.00729000 -4.23520100

C 2.09763700 -3.89014500 -3.15427000

H 1.65140300 -4.55842200 -3.90024600

H 1.77399800 -4.26479000 -2.17569400

C 3.62583500 -3.97125100 -3.28034300

H 3.94028500 -5.01417500 -3.14718200

H 3.90161000 -3.69856700 -4.30962200

C 4.42284300 -3.07994700 -2.31655100

H 5.49222200 -3.21667700 -2.52417800

H 4.26223700 -3.38041500 -1.27536100

C 4.09687800 -1.58150000 -2.42210100

H 4.05812300 -1.28003900 -3.48097100

H 4.91367500 -1.01267400 -1.96918100

C 1.24751700 1.90651100 -1.37454400

N 2.51149700 1.55492300 -1.22109400

O 0.29776900 1.36091700 -0.69407300

C 0.80635200 2.95474700 -2.38346400

H 1.01455500 2.56508100 -3.39050300

H -0.27849900 3.02876100 -2.29657400

C 1.44586300 4.34926400 -2.22945000

H 1.41198600 4.64965600 -1.17895600

H 0.82014400 5.06534500 -2.77523900

C 2.88631100 4.45977300 -2.73644500

H 3.21865400 5.50157600 -2.64534000

H 2.91142500 4.22037500 -3.80958600

C 3.87441100 3.54680200 -2.00409500

H 4.88108800 3.71202800 -2.40862500

H 3.91217600 3.79966800 -0.93703500

C 3.54828400 2.05089600 -2.13470000

H 4.44900300 1.47347100 -1.91238300

H 3.27805600 1.81712800 -3.17620400

C 2.06040100 0.81154200 2.56883700

N 0.86781400 0.30980700 2.26653200

O 2.95694300 1.07042100 1.68709400

C 2.47984400 1.13790400 3.99602400

H 2.59305000 0.18968600 4.54163700

H 3.47874300 1.57018700 3.91100900

C 1.55643000 2.07792500 4.79131900

H 1.25442500 2.92142600 4.15632300

H 2.13739500 2.50543500 5.61697200

C 0.31603800 1.38662700 5.36390700

H -0.23675000 2.09114300 5.99766100

H 0.64178900 0.56829500 6.02213000

C -0.62378800 0.82332800 4.29551700

H -1.47421600 0.33592700 4.79010600

H -1.04161200 1.64772500 3.70766500

C 0.00742000 -0.21117700 3.34583600

H -0.79493300 -0.74738500 2.84441000

H 0.55986500 -0.95830000 3.93495000

Rh 0.57228500 -0.43909000 0.33059200

C -1.57006100 -0.56151300 0.32800000

O -2.22236400 3.12340500 -0.85858800

C -0.62685000 3.97133200 0.55981300

C 0.51828600 3.68447000 1.32390800

C -0.91568800 5.29013800 0.16393000

C 1.35592600 4.72741700 1.70591700

H 0.78019000 2.66160600 1.54855800

C -0.07477200 6.31906500 0.56855400

H -1.79815700 5.47650200 -0.43601400

C 1.06231100 6.04496100 1.33972100

H 2.25433800 4.49825600 2.27023600

H -0.30365100 7.34064900 0.28124500

H 1.72141100 6.85307700 1.64144400

N -1.50679500 2.94361200 0.14411300

III-ts1

0 1

C -2.40683200 0.42390000 0.58168100

C -1.97838700 1.58547100 1.25431400

O -3.77123500 0.26775000 0.36574800

H -2.73150100 2.19339300 1.74809600

H -0.98995900 1.56675700 1.68026200

Si -4.93890800 0.93648100 -0.65559700

C -5.26524200 2.72508800 -0.15037300

H -5.43652300 2.77754400 0.93122200

H -6.16838200 3.09729400 -0.64641800

H -4.43920700 3.39098200 -0.40376900

C -4.50850400 0.75691900 -2.50935500

C -6.49735700 -0.03933400 -0.23943700

H -7.33218400 0.29891500 -0.86397700

H -6.78272100 0.11021700 0.80728000

H -6.37070600 -1.11151600 -0.40711300

C -5.38768100 1.74952400 -3.30391300

H -5.20766400 1.62842900 -4.38090700

H -5.16365300 2.79114400 -3.04858800

H -6.45988100 1.58427800 -3.13588800

C -4.81844300 -0.67949500 -2.98756700

H -5.87798900 -0.93398500 -2.86668900

H -4.21028400 -1.41853800 -2.45940100

H -4.58217200 -0.77135800 -4.05701400

C -3.02092500 1.06610400 -2.78127700

H -2.82654400 1.01038500 -3.86236800

H -2.35691100 0.34940800 -2.29187200

H -2.75241300 2.07144500 -2.44657000

N -2.80723100 -2.77132100 0.56636100

C -3.17218000 -2.53446100 1.96116200

H -2.79115000 -3.37155300 2.56699200

H -2.68312200 -1.62421400 2.31044100

C -4.69530900 -2.42213000 2.12468400

H -5.01656200 -1.49998200 1.63491500

H -4.93877400 -2.33580000 3.19091700

C -5.41311200 -3.63027700 1.50307300

H -6.49923400 -3.49136200 1.55245200

H -5.18365700 -4.53178400 2.09017200

C -4.96297900 -3.85334700 0.05043500

H -5.28825300 -3.01731900 -0.57959700

H -5.41361900 -4.76502100 -0.35968000

C -3.43488700 -3.94864000 -0.03282500

H -3.09032100 -4.02042700 -1.06418000

H -3.08778200 -4.83729200 0.51503000

C -2.21425100 -1.83813800 -0.25160000

O -2.17694100 -1.97254900 -1.47737900

Rh 2.93102700 0.07919000 -0.12627300

C 2.51763500 -2.41773600 1.33986900

N 1.21939300 -2.21446300 1.19487100

O 3.42179100 -1.56266100 1.02633600

C 3.05754900 -3.74466000 1.86081500

H 4.12855300 -3.59397300 2.01017600

H 2.61936100 -3.99091800 2.83641900

C 2.81949300 -4.90832500 0.87375900

H 3.52294800 -5.71493400 1.11345600

H 3.07498400 -4.56832700 -0.13629000

C 1.39492100 -5.48259000 0.89003100

H 1.21845900 -5.93483700 1.87731100

H 1.33311000 -6.30275300 0.16352200

C 0.27409200 -4.46945800 0.61896300

H 0.32571100 -4.07272400 -0.40125800

H -0.69125600 -4.98266100 0.70912500

C 0.28742900 -3.27439600 1.58340600

H 0.49621900 -3.62099600 2.60720100

H -0.69779000 -2.82923500 1.60735000

C 1.70263800 -1.62945000 -2.13540900

N 2.86289200 -1.13755900 -1.75353300

O 0.59769900 -1.41361900 -1.51583500

C 1.56621900 -2.54664300 -3.34070100

H 0.49413400 -2.67174700 -3.50388500

H 1.98784500 -2.06467500 -4.23316900

C 2.23989900 -3.91663400 -3.12533600

H 1.82600300 -4.62551500 -3.85264700

H 1.96051500 -4.29565500 -2.13479900

C 3.76801300 -3.90038400 -3.27815600

H 4.15223700 -4.91857700 -3.13632800

H 4.00658500 -3.62568200 -4.31626800

C 4.52132600 -2.94333300 -2.34227700

H 5.59397000 -3.01659700 -2.56558900

H 4.39600700 -3.23570000 -1.29375300

C 4.09770800 -1.47116000 -2.46766400

H 4.02507100 -1.19293900 -3.53146300

H 4.88370000 -0.84270500 -2.03912400

C 1.06954700 1.84430500 -1.51356800

N 2.35087900 1.59989100 -1.33660900

O 0.15627100 1.30984800 -0.77317200

C 0.55264900 2.76312600 -2.61160300

H 0.81993200 2.31592700 -3.57997100

H -0.53503500 2.73555400 -2.54589400

C 1.04819300 4.22201200 -2.57165300

H 0.94864200 4.61412800 -1.55558300

H 0.37828800 4.82113000 -3.20012800

C 2.48916300 4.42230100 -3.04916500

H 2.72609300 5.49376600 -3.03417000

H 2.56701300 4.10387700 -4.09911800

C 3.53284500 3.65975700 -2.22618500

H 4.53204200 3.89850400 -2.61260500

H 3.50672000 3.98595200 -1.17864200

C 3.35858400 2.13252700 -2.25916700

H 4.30642500 1.66721600 -1.97528900

H 3.15024900 1.80270600 -3.28935200

C 2.03907800 0.89874900 2.51926200

N 0.85885700 0.34770700 2.24930500

O 2.88336700 1.21967500 1.60998500

C 2.49765500 1.21921900 3.93607300

H 2.67308300 0.26744200 4.45859700

H 3.47342400 1.69511600 3.82056300

C 1.56774300 2.10519200 4.78299900

H 1.20241800 2.94094800 4.17150000

H 2.16170100 2.54839600 5.59108700

C 0.38720700 1.34574900 5.39294700

H -0.17286100 2.01051200 6.06242900

H 0.77904000 0.53224700 6.02060600

C -0.56686700 0.75714200 4.35143400

H -1.37256300 0.22116300 4.87076200

H -1.04593500 1.57184900 3.79754900

C 0.06861600 -0.22916200 3.35391700

H -0.73341500 -0.78917800 2.87784600

H 0.67235300 -0.96690900 3.90312800

Rh 0.55562300 -0.45894200 0.31582400

C -1.54582500 -0.64234300 0.35341900

O -2.22926500 3.63345400 -0.75708700

C -0.47389200 4.11547900 0.57726400

C 0.68688200 3.58405800 1.15526000

C -0.64367800 5.50587200 0.43154400

C 1.69190500 4.44774600 1.58272100

H 0.82675600 2.51669400 1.18945700

C 0.35523600 6.35532200 0.88616100

H -1.55143600 5.88094400 -0.02729500

C 1.52546400 5.82932800 1.45663300

H 2.60530800 4.02323400 1.98577000

H 0.23196900 7.43024400 0.79535500

H 2.30790500 6.50269600 1.79409100

N -1.47184600 3.21905400 0.10500200

III-ts2

0 1

C -2.06892600 0.59972700 -0.36239600

C -2.93437300 0.60171900 -1.65730500

O -2.18195000 1.76133100 0.28524000

H -3.96475200 0.85683900 -1.41753200

H -2.50382400 1.36804300 -2.31366300

Si -3.10279600 3.21037900 0.30797700

C -3.62060400 3.44946700 2.10011100

H -3.88018800 2.49030200 2.55938000

H -4.50749100 4.09138300 2.13617300

H -2.84369000 3.91295800 2.71105800

C -1.98499900 4.59943900 -0.34404900

C -4.70315100 3.10227800 -0.69000600

H -5.24687700 4.04021400 -0.52494000

H -5.35272200 2.29320000 -0.33811300

H -4.55690000 2.99743700 -1.76733300

C -2.62114000 5.96199800 0.00790600

H -1.99503700 6.77623600 -0.37976600

H -2.70993600 6.10738500 1.09038400

H -3.61825600 6.08353000 -0.43295100

C -1.85700000 4.47289700 -1.87473500

H -2.81726000 4.60857700 -2.38541600

H -1.45012600 3.49745600 -2.15173600

H -1.16800300 5.23353300 -2.26308700

C -0.58575300 4.49721800 0.29806400

H 0.05985700 5.29619000 -0.09116000

H -0.10740500 3.53550900 0.08865700

H -0.63615800 4.62780600 1.38389100

N -2.89131800 -1.89812700 1.14919900

C -3.37259300 -0.83712200 2.02966400

H -3.18932900 -1.14172200 3.07377100

H -2.80874600 0.07884000 1.87011300

C -4.87753200 -0.62003300 1.82488600

H -5.04387800 -0.26940800 0.79707600

H -5.23786900 0.16298200 2.50371800

C -5.63843700 -1.93603500 2.05440500

H -6.70453400 -1.80906100 1.83393000

H -5.56490100 -2.20292000 3.11845700

C -5.04515900 -3.06983700 1.20227000

H -5.24000700 -2.87472400 0.14352500

H -5.52263500 -4.02533900 1.44998300

C -3.52714000 -3.19114300 1.39835500

H -3.08924100 -3.91489000 0.71296600

H -3.30000600 -3.51364100 2.42731800

C -1.82172900 -1.81811500 0.25964300

O -1.37812500 -2.83642300 -0.26113500

Rh 3.21073600 0.23558100 -0.12488800

C 2.65123200 -1.77436200 1.91983800

N 1.37185500 -1.62345600 1.61730700

O 3.60488900 -1.09600800 1.39430500

C 3.12741900 -2.81856400 2.92579800

H 4.20342200 -2.66121900 3.02232400

H 2.68198900 -2.62751100 3.91152500

C 2.84088300 -4.27457700 2.50340300

H 3.52243100 -4.93376400 3.05495200

H 3.08872500 -4.39353100 1.44147000

C 1.39883100 -4.72927000 2.75947700

H 1.21426300 -4.69496700 3.84391000

H 1.29413500 -5.78125000 2.46452100

C 0.32908200 -3.89084900 2.05113200

H 0.39353600 -3.98820200 0.96427200

H -0.66140900 -4.26988400 2.33430400

C 0.38877800 -2.39343100 2.39228500

H 0.56878700 -2.26811900 3.47169400

H -0.58405100 -1.94831200 2.21080200

C 2.05386800 -1.87966300 -1.73708500

N 3.21128000 -1.34118600 -1.41303500

O 0.92760900 -1.41728600 -1.33546100

C 1.95074600 -3.15327400 -2.55790500

H 0.89621900 -3.26177900 -2.81754400

H 2.52749200 -3.07118900 -3.48854200

C 2.43388500 -4.37459200 -1.74744300

H 2.02156000 -5.28397700 -2.20160000

H 2.00757700 -4.31100600 -0.73896900

C 3.96230000 -4.51681700 -1.67475900

H 4.20886100 -5.42466100 -1.10878700

H 4.34150600 -4.68121400 -2.69449900

C 4.71865000 -3.32606000 -1.06510400

H 5.79383500 -3.54819600 -1.09821800

H 4.45516200 -3.18526900 -0.01156700

C 4.47253800 -1.98747500 -1.78054400

H 4.53806500 -2.12735400 -2.87139600

H 5.27301700 -1.29051500 -1.51330500

C 1.48992900 1.80376700 -1.88476300

N 2.73925100 1.47806000 -1.65737900

O 0.51419100 1.45352800 -1.11536000

C 1.10838100 2.66310600 -3.07922800

H 1.48435800 2.19526000 -3.99876000

H 0.02094900 2.64913000 -3.14477100

C 1.63336300 4.10988600 -2.98357700

H 1.46348200 4.48349200 -1.96651500

H 1.03859700 4.74481200 -3.65221300

C 3.11473200 4.25595300 -3.35900400

H 3.39826100 5.31433500 -3.29778700

H 3.23409600 3.96671400 -4.41369400

C 4.08752900 3.42168600 -2.51326200

H 5.10718100 3.60303100 -2.87777700

H 4.05890100 3.74121800 -1.46419800

C 3.81446100 1.90801700 -2.55147400

H 4.71591900 1.37722200 -2.23013500

H 3.62516500 1.58528200 -3.58776400

C 2.14731200 1.80637600 2.07542900

N 1.01623800 1.13976500 1.90233100

O 3.11300600 1.79690900 1.23288500

C 2.40923600 2.64709300 3.32029600

H 2.48498200 1.97505100 4.18734300

H 3.39903500 3.08375900 3.17315600

C 1.38013300 3.75198700 3.61315900

H 1.15947000 4.28739300 2.68225500

H 1.84184500 4.48262400 4.28853700

C 0.08287000 3.24621200 4.25512200

H -0.56973600 4.10162300 4.47718000

H 0.32857100 2.79022600 5.22552000

C -0.68752100 2.22602800 3.41285000

H -1.59051300 1.92463700 3.96081900

H -1.01860900 2.67731200 2.47734500

C 0.09718700 0.96687900 3.03333500

H -0.61278600 0.19956400 2.73224100

H 0.62789100 0.56828200 3.91235200

Rh 0.80893300 -0.11565400 0.26322300

C -1.27946700 -0.42867500 0.09660200

O -1.63312100 -1.01392700 -2.47230400

C -3.88897500 -1.62954800 -2.30845700

C -5.23827300 -1.22727300 -2.31037000

C -3.55440600 -2.99286900 -2.40590500

C -6.24099400 -2.19143300 -2.37821300

H -5.50454700 -0.17608700 -2.29532700

C -4.57106700 -3.93709100 -2.47412900

H -2.50864300 -3.26933500 -2.37759300

C -5.91650600 -3.54837700 -2.46053100

H -7.28057000 -1.87764700 -2.38111600

H -4.31152700 -4.99006300 -2.53020600

H -6.70272700 -4.29459100 -2.51740100

N -2.84699300 -0.68313100 -2.27790400

int2

0 1

O -1.06401000 1.71522700 -0.98425800

C 1.04739900 2.63456500 -0.46749900

C 2.36499800 3.00164600 -0.78392500

C 0.57480500 2.81901600 0.83670800

C 3.20785100 3.50276200 0.20581900

H 2.72287100 2.90855000 -1.80472400

C 1.42744600 3.32930500 1.81580200

H -0.44571500 2.55287900 1.07698600

C 2.74721600 3.66489300 1.51396600

H 4.22679000 3.77617000 -0.05260900

H 1.05252900 3.45432100 2.82746400

H 3.40551700 4.05595100 2.28326600

N 0.21992800 2.12489300 -1.52429100

C -0.93818000 0.34779300 -0.66161700

C 0.12136100 -0.19592600 -1.28792300

C 0.73566000 0.89220900 -2.15313900

O 0.55268300 -1.46131100 -1.27372900

H 1.82428300 0.88768400 -2.15062100

H 0.38589000 0.82135800 -3.19126100

Si 2.04548600 -2.09649100 -0.73405900

C 3.45152400 -1.18694900 -1.60697400

H 4.38603800 -1.74393500 -1.47974600

H 3.61190700 -0.18539800 -1.19422400

H 3.26613000 -1.09431300 -2.68217500

C 2.23901900 -1.93379900 1.15225900

C 1.95299200 -3.88177600 -1.29653900

H 2.82150600 -4.45009100 -0.94665700

H 1.92114600 -3.94936200 -2.38851400

H 1.05296100 -4.35994300 -0.89939800

C 3.70651900 -2.25911100 1.51419300

H 3.83311000 -2.23181400 2.60455900

H 4.41033000 -1.53583900 1.08758300

H 4.00614100 -3.26068000 1.18057400

C 1.30514200 -2.92621200 1.87695700

H 1.53755200 -3.96616500 1.61956100

H 0.25698700 -2.72846600 1.64129100

H 1.42899800 -2.82568100 2.96415700

C 1.90665300 -0.49788700 1.61110700

H 2.12988100 -0.38689600 2.68105700

H 0.84643700 -0.27864500 1.47125700

H 2.48938700 0.26566600 1.08232000

N -3.08036800 0.29171000 0.54194800

C -3.83632000 1.04264100 -0.46958200

H -4.34737000 1.86994200 0.04095300

H -3.15624300 1.48337600 -1.19519300

C -4.86847100 0.13284300 -1.15581400

H -4.32921300 -0.62768400 -1.73571400

H -5.45694900 0.72514900 -1.86661100

C -5.78198900 -0.55114100 -0.12795300

H -6.46379400 -1.24947200 -0.62608200

H -6.40914900 0.21088600 0.35698600

C -4.95268700 -1.27474900 0.94400300

H -4.40555500 -2.11385900 0.49717100

H -5.60139300 -1.68700000 1.72568400

C -3.93131000 -0.31689200 1.57165900

H -3.28624100 -0.83271300 2.28191700

H -4.45102000 0.49867500 2.09259300

C -1.84689600 -0.27526600 0.32702600

O -1.44790400 -1.25088800 0.97050800

int3

0 1

O -1.76157400 0.43013300 -1.93638000

C -3.37714200 -1.36182300 -0.25968000

C -3.18247900 -0.14764900 0.48365400

C -4.73784700 -1.80254100 -0.42205300

C -4.24626400 0.46743300 1.13190400

H -2.19464400 0.27676100 0.57308500

C -5.78647400 -1.15229800 0.19083900

H -4.89077000 -2.69937000 -1.01276300

C -5.54920600 -0.01269500 0.98359900

H -4.06527400 1.36493000 1.71736400

H -6.80059400 -1.52007100 0.06409600

H -6.37932800 0.50987900 1.44916000

N -2.46238500 -2.19787500 -0.75441400

C -0.68157300 0.36475100 -1.32518400

C -0.34068200 -0.73967500 -0.47413800

C -1.05536000 -2.04721500 -0.57830000

O 0.69194600 -0.56452400 0.35355600

H -0.59773500 -2.52530900 -1.46525800

H -0.74857300 -2.69815400 0.25195500

Si 2.00576800 -1.43890600 1.01767600

C 1.43195500 -3.10466300 1.68364700

H 2.22287400 -3.53143300 2.31030800

H 1.21938300 -3.82689000 0.88945100

H 0.53777400 -3.00349900 2.30742700

C 3.32534000 -1.64109400 -0.33816600

C 2.54747400 -0.32348300 2.42419900

H 3.45755600 -0.70400100 2.90025700

H 1.76933200 -0.25573700 3.19117800

H 2.75466000 0.68661000 2.05969600

C 4.35189000 -2.69639800 0.12958400

H 5.14145200 -2.80704400 -0.62512800

H 3.89770600 -3.68307400 0.27254700

H 4.84257500 -2.41086700 1.06859200

C 4.04618900 -0.29985300 -0.59166900

H 4.55837500 0.06380300 0.30674000

H 3.35234600 0.46850000 -0.94349600

H 4.80924800 -0.43092900 -1.37049500

C 2.65134600 -2.11032900 -1.64581100

H 3.41555700 -2.31585000 -2.40690900

H 1.98903100 -1.33793800 -2.04792300

H 2.08091300 -3.03790900 -1.50739500

N 0.20629700 2.65423500 -0.99390600

C -0.83857000 2.91964500 -0.00185600

H -1.21839900 3.93378700 -0.18104200

H -1.67433200 2.23825800 -0.16038600

C -0.25336700 2.81692900 1.41443200

H 0.05634000 1.77783500 1.58475400

H -1.02354500 3.05908400 2.15637700

C 0.95779600 3.75500900 1.56287400

H 1.42503300 3.62137000 2.54509800

H 0.60832800 4.79643600 1.52008900

C 1.99080300 3.52756800 0.44541700

H 2.44991300 2.53707200 0.55563100

H 2.79732500 4.26702900 0.50986800

C 1.33203300 3.59224000 -0.94150900

H 2.03141800 3.32452500 -1.73318100

H 0.94642400 4.60049100 -1.13871900

C 0.42131000 1.41336500 -1.51174000

O 1.43193600 1.09795400 -2.14348400

int4

0 1

O 1.08086000 -2.11138600 0.27556700

C -0.82207500 2.10451800 -0.39388500

C -1.56072700 2.81143200 -1.33712300

C -1.03486600 2.24766300 0.97722500

C -2.56138200 3.67758900 -0.89241800

H -1.36467100 2.66122600 -2.39359800

C -2.03022400 3.12288600 1.40749500

H -0.39903600 1.69408400 1.66122200

C -2.79574800 3.83072600 0.47577500

H -3.16090500 4.22433000 -1.61345500

H -2.20738400 3.25422100 2.47026900

H -3.57701800 4.50298200 0.81713600

N 0.21614400 1.18759400 -0.81815900

C 1.13402500 -0.88008300 0.07147800

C 0.07107800 -0.14397200 -0.51732200

C 1.31564400 1.65768700 -1.39879100

O -1.15813000 -0.65392200 -0.75395200

H 1.58086300 2.69516900 -1.24848900

H 1.97093000 0.96328000 -1.90252700

Si -2.06669300 -1.76855600 0.17149500

C -1.63118800 -1.57013100 1.98767800

H -2.34512000 -2.10632100 2.62235900

H -1.64663100 -0.51568800 2.28076300

H -0.62963500 -1.96396800 2.17736200

C -3.86177900 -1.22096800 -0.18249800

C -1.75418400 -3.50555900 -0.46178100

H -2.32581000 -4.24501000 0.10969400

H -0.68915000 -3.72581700 -0.36275300

H -2.03454000 -3.60207900 -1.51543500

C -4.83901600 -2.23596400 0.44660200

H -5.87817000 -1.92974400 0.26332300

H -4.71107100 -2.31030600 1.53324300

H -4.71583600 -3.23988500 0.02490200

C -4.10058200 -1.15780700 -1.70539400

H -3.96407600 -2.13404100 -2.18470600

H -3.41849800 -0.44886500 -2.18552700

H -5.12871700 -0.83171300 -1.91678500

C -4.12068700 0.17571000 0.41776600

H -5.15459400 0.49097600 0.21815500

H -3.45685600 0.92988000 -0.01325800

H -3.97998100 0.18831400 1.50434000

N 3.53422200 -0.48756700 0.56333600

C 3.95731800 -1.46285000 -0.44279600

H 4.45579000 -2.29621800 0.07308200

H 3.08437500 -1.87845400 -0.94111700

C 4.92942800 -0.81816300 -1.44093100

H 4.38776400 -0.06030300 -2.02344900

H 5.28050400 -1.57871300 -2.14795000

C 6.11105300 -0.15956100 -0.71468500

H 6.76962400 0.34577000 -1.42996100

H 6.71238500 -0.94089100 -0.22833300

C 5.61022700 0.82609400 0.35077700

H 5.09966300 1.66826800 -0.13415700

H 6.44861100 1.24046200 0.92245300

C 4.62590100 0.14219600 1.30866300

H 4.18239200 0.84918000 2.00927700

H 5.14776800 -0.63660400 1.88424600

C 2.25243100 -0.07561500 0.76792200

O 1.94980700 0.81544600 1.56713000

int5

0 1

O 1.71042000 -0.63319700 -1.35693300

C -2.97929200 -1.07678600 -0.47931400

C -4.29531200 -1.03423300 -0.95400200

C -2.70785300 -1.57009100 0.80383600

C -5.34377500 -1.42962400 -0.12448900

H -4.49605700 -0.72924200 -1.97541400

C -3.76206400 -1.96761100 1.61907700

H -1.68610400 -1.65618300 1.14634900

C -5.08207000 -1.89076300 1.16532200

H -6.36240800 -1.39473600 -0.49773300

H -3.54929700 -2.34566600 2.61393300

H -5.89827500 -2.20731100 1.80707800

N -1.90967200 -0.59973300 -1.30347800

C 0.45056900 -0.15507400 -1.22868300

C -0.54595000 -1.15329200 -1.12443900

C -2.10067300 0.30717000 -2.23870000

O -0.41146200 -2.36283600 -0.90245800

H -2.97815700 0.94262600 -2.19673900

H -1.36207600 0.43199200 -3.01554900

Si 3.07017400 -0.40027400 -0.37177000

C 3.15562700 1.37142500 0.25963500

H 4.10496000 1.54576600 0.77743700

H 2.34623500 1.60082600 0.95881900

H 3.07366000 2.07005100 -0.57745900

C 2.92383900 -1.64661200 1.06590000

C 4.51542200 -0.77931700 -1.50384000

H 5.46656700 -0.76627700 -0.96108300

H 4.56959200 -0.03462800 -2.30403400

H 4.39933700 -1.76402100 -1.96575200

C 4.13326300 -1.49300600 2.01055700

H 4.06997000 -2.22090900 2.83095800

H 4.17825700 -0.49503700 2.46271000

H 5.08369500 -1.67066600 1.49334700

C 2.87501200 -3.07961600 0.49484200

H 3.78632300 -3.32807100 -0.06110200

H 2.01328600 -3.20502600 -0.16840000

H 2.78335500 -3.80917600 1.31178500

C 1.61956400 -1.39303500 1.85176200

H 1.56323100 -2.06520900 2.71938700

H 0.74923200 -1.60094600 1.22207000

H 1.55136000 -0.36671200 2.23321900

N -0.43562800 1.99772600 -0.33252900

C -0.96815300 1.42456000 0.89911500

H -2.06710800 1.38632300 0.84492700

H -0.60841400 0.40243500 0.99150000

C -0.54289000 2.23883200 2.13068000

H 0.53656900 2.11183200 2.28044200

H -1.04097200 1.82511500 3.01534400

C -0.86259500 3.72912600 1.96465900

H -0.49740200 4.29830600 2.82666700

H -1.95337300 3.86524000 1.93344900

C -0.25015700 4.25770800 0.66097000

H 0.84439000 4.20580800 0.71490800

H -0.52159100 5.30717300 0.49852500

C -0.72087100 3.41922000 -0.53131600

H -0.22910100 3.72624000 -1.45348300

H -1.81041600 3.54021800 -0.65625200

C 0.33698100 1.33788600 -1.27125500

O 0.98650000 1.96060400 -2.11747600

ts1

0 1

Rh -3.15833400 -0.93607300 0.54992900

O -3.08659900 0.60256600 1.91135400

C -2.06922800 1.35896800 1.92632400

O -1.04415500 1.26497700 1.16993100

Rh -0.93417100 -0.26457700 -0.20866700

O -0.17326100 -1.52957100 1.22388000

C -0.97328800 -2.20170700 1.95823800

O -2.24091400 -2.16921700 1.91482200

O -1.86316000 0.91069900 -1.62565000

C -3.14086800 0.97959000 -1.63100400

O -3.92875000 0.35446200 -0.86107000

O -1.00598000 -1.84680800 -1.53327400

C -2.05658700 -2.57640200 -1.57118000

O -3.09030400 -2.44041900 -0.85198900

C 1.05854900 0.30738200 -0.75743000

C 1.97435200 -0.47684700 -1.43854200

C 1.29664900 -0.16421100 -2.62292500

O 3.23620200 -0.78781400 -1.30904100

H 0.27906600 -0.47623500 -2.80476000

H 1.85963100 0.30420500 -3.43087300

Si 4.19480600 -1.43458700 -0.00285400

C 4.65019300 -3.13079100 -0.67640500

H 5.38792100 -3.62172300 -0.03162700

H 5.06905600 -3.06676800 -1.68469000

H 3.76385600 -3.77330300 -0.71608800

C 5.70865500 -0.28391400 0.08129700

C 3.22987100 -1.61581400 1.58583900

H 3.65061400 -2.45108900 2.15850600

H 2.17468400 -1.82518800 1.38327600

H 3.25925600 -0.70466900 2.18303600

C 6.55191400 -0.68653500 1.31200600

H 7.44822400 -0.05544300 1.37704700

H 6.89371200 -1.72760400 1.25825700

H 5.99440200 -0.56093700 2.24673100

C 5.27775000 1.19073800 0.21900200

H 4.65197800 1.35895400 1.09849100

H 4.70314500 1.52266600 -0.65251800

H 6.16665800 1.83184000 0.29667500

C 6.55992200 -0.44306400 -1.19647200

H 7.42412600 0.23378900 -1.16019600

H 5.98704400 -0.19733100 -2.09772100

H 6.94713500 -1.46130900 -1.30997800

N 1.41018500 2.71512300 -0.39712400

C 0.48965100 3.09448200 -1.47381100

H 1.04749100 3.71327200 -2.19229200

H 0.13735500 2.20465900 -1.98927100

C -0.70612500 3.88177400 -0.91824400

H -1.30621300 3.19377400 -0.31672800

H -1.32567700 4.22074500 -1.75732000

C -0.24872400 5.07195100 -0.06227800

H -1.11383500 5.57492900 0.38561100

H 0.25023100 5.81463200 -0.70200000

C 0.73034900 4.60901400 1.02814700

H 0.21716300 3.94487300 1.73199100

H 1.11568400 5.46411300 1.59597800

C 1.90176300 3.83661100 0.40909000

H 2.56783000 3.43558000 1.17216500

H 2.48223900 4.49826400 -0.24975800

C 1.61434700 1.44705400 0.04801200

O 2.23573600 1.17220800 1.07854800

C -0.33230400 -3.14575800 2.94917400

H -0.08314900 -4.07982100 2.43408600

H 0.59355800 -2.71367100 3.33355300

H -1.02446200 -3.36918500 3.76176400

C -2.05082800 -3.69335000 -2.58953500

H -2.10169400 -3.26135100 -3.59416500

H -1.11445500 -4.25227100 -2.51748200

H -2.90325400 -4.35505000 -2.43577100

C -3.74708200 1.91386500 -2.65258800

H -3.44093200 2.93884200 -2.42186100

H -3.36177100 1.67136000 -3.64667700

H -4.83450600 1.84188700 -2.64131300

C -2.06145900 2.50399500 2.91260600

H -1.10513000 2.52783700 3.44061400

H -2.16174600 3.44627400 2.36390800

H -2.88647500 2.40716000 3.61841500

ts3

0 1

O -1.73561600 0.05978400 -1.92986200

C -3.37303300 -1.18545000 -0.23075300

C -3.26099400 -0.49948200 1.00923800

C -4.64482100 -1.24084400 -0.84994700

C -4.38345400 0.04525700 1.61216500

H -2.29028200 -0.38427600 1.47951300

C -5.76241500 -0.67849100 -0.24470000

H -4.70904600 -1.74362400 -1.80865000

C -5.63937800 -0.03905900 0.99106700

H -4.28642100 0.55946300 2.56411800

H -6.72974600 -0.73525300 -0.73452700

H -6.50923200 0.40528300 1.46539900

N -2.36884100 -1.82513600 -0.91259300

C -0.68326700 0.20514300 -1.22785700

C -0.32394400 -0.69056500 -0.24034800

C -1.08661400 -2.00225000 -0.30001000

O 0.67581200 -0.45944200 0.64398100

H -0.52463600 -2.76512100 -0.85603900

H -1.20298000 -2.36701700 0.72995300

Si 2.04832900 -1.39453400 1.04311800

C 1.48695500 -3.09265500 1.64057500

H 2.32783100 -3.62680700 2.09585000

H 1.10386800 -3.71462000 0.82606600

H 0.70270100 -3.00335700 2.39965000

C 3.22774500 -1.54058200 -0.44478300

C 2.79635200 -0.40864500 2.45450000

H 3.74931900 -0.83944100 2.77889200

H 2.12285500 -0.39001200 3.31727800

H 2.98096500 0.62566000 2.14818300

C 4.34841400 -2.53735100 -0.07007100

H 5.06595400 -2.61789900 -0.89705200

H 3.95908900 -3.54339900 0.12187500

H 4.91147600 -2.21734700 0.81555300

C 3.85720100 -0.16977500 -0.77269400

H 4.41047700 0.24130000 0.07965000

H 3.10389900 0.55487500 -1.09241600

H 4.56876800 -0.27627900 -1.60236300

C 2.47374400 -2.06159200 -1.68786500

H 3.18349900 -2.23164600 -2.50852500

H 1.73654500 -1.33804100 -2.04724600

H 1.97147900 -3.01793900 -1.49601800

N 0.22269800 2.45141700 -0.92287700

C -0.74361200 2.68718300 0.15368300

H -1.23083400 3.65269800 -0.04308700

H -1.51900300 1.92095400 0.12353100

C -0.03431900 2.72677400 1.51448900

H 0.35880300 1.72659400 1.72452300

H -0.76105100 2.97351200 2.29772000

C 1.11186300 3.75134900 1.49800500

H 1.66256800 3.72227100 2.44499600

H 0.69115800 4.76333900 1.40920100

C 2.06409900 3.50078500 0.31682300

H 2.58289600 2.54334200 0.45194500

H 2.83121600 4.28209300 0.26487500

C 1.29296300 3.44819000 -1.01010100

H 1.93514900 3.17585000 -1.84744700

H 0.83923100 4.42562200 -1.22407800

C 0.33443300 1.28432000 -1.61504100

O 1.18009900 1.07030400 -2.47848500

ts4

0 1

O -0.61161300 -2.34755800 -0.18299300

C 0.19382800 2.32416400 0.40653500

C 0.06252800 3.42178800 1.26230800

C 0.74410500 2.47875500 -0.87227200

C 0.49092800 4.67825300 0.83543000

H -0.35768000 3.27643800 2.25295400

C 1.19183500 3.73421200 -1.27401400

H 0.76674400 1.62795700 -1.54405100

C 1.06711800 4.83897300 -0.42623900

H 0.38811000 5.53146900 1.49968200

H 1.62092200 3.85425000 -2.26438500

H 1.41031500 5.81684200 -0.74930700

N -0.24277100 1.05842700 0.81597200

C -0.80510000 -1.13444100 -0.10179800

C 0.15555000 -0.20716400 0.49740500

C -1.07017500 0.55777900 1.80587200

O 1.41502500 -0.56931300 0.74030400

H -2.13312000 0.79882000 1.79710300

H -0.62513900 0.17809700 2.72187400

Si 2.44279400 -1.68639300 -0.07998900

C 1.91177400 -1.80903400 -1.87911500

H 2.77455000 -1.97223300 -2.53340100

H 1.41593700 -0.88743000 -2.20239300

H 1.20010200 -2.62561000 -2.01760500

C 4.12882000 -0.81765600 0.08755300

C 2.39090400 -3.32786600 0.82139600

H 3.07096700 -4.05146800 0.35870000

H 1.37351200 -3.72363700 0.77517000

H 2.67741800 -3.21858900 1.87190400

C 5.23737900 -1.72465900 -0.48569200

H 6.21304000 -1.22571000 -0.41207800

H 5.07397400 -1.95848000 -1.54445200

H 5.31438800 -2.67228000 0.05966700

C 4.42442100 -0.52354200 1.57279200

H 4.49048100 -1.44210300 2.16737700

H 3.65045600 0.11141400 2.01523100

H 5.38524900 -0.00042000 1.67276700

C 4.09848200 0.51422600 -0.69274800

H 5.06210000 1.03285200 -0.59644900

H 3.32097000 1.18778100 -0.31795000

H 3.91888800 0.35742900 -1.76273000

N -3.23080000 -0.87313000 -0.57934800

C -3.58941400 -1.79880400 0.49694000

H -3.95116300 -2.73514500 0.04753600

H -2.70103200 -2.04287300 1.07698100

C -4.68263700 -1.19480200 1.38782900

H -4.26447600 -0.33676000 1.92973800

H -4.98348400 -1.93561200 2.13733800

C -5.88826500 -0.73685900 0.55578000

H -6.63857700 -0.26170600 1.19716800

H -6.36909800 -1.61554800 0.10271100

C -5.44010500 0.22300400 -0.55544600

H -5.04862100 1.14719400 -0.11152000

H -6.28530500 0.50203000 -1.19471600

C -4.34261500 -0.41355600 -1.41608200

H -3.93674000 0.28660100 -2.14630200

H -4.75066100 -1.27926900 -1.95909000

C -1.96393100 -0.48075900 -0.87029100

O -1.66087600 0.28887700 -1.78241600

ts5

0 1

O 0.79613100 1.31749400 -0.44024500

C -1.74513000 -2.39040000 0.30942900

C -2.46723700 -3.15557300 1.22218300

C -2.19616500 -2.18339600 -0.99467700

C -3.67080200 -3.73289000 0.81560200

H -2.10288500 -3.26852700 2.23816100

C -3.40014700 -2.76048600 -1.38646400

H -1.59733300 -1.59291400 -1.67873600

C -4.13721900 -3.53455600 -0.48446200

H -4.24772800 -4.32386200 1.51967600

H -3.76182600 -2.60828800 -2.39806600

H -5.07796300 -3.97806000 -0.79530600

N -0.48064000 -1.81119000 0.70276200

C 0.89474400 0.03216200 -0.17789700

C -0.26227800 -0.46260700 0.39295400

C 0.43363200 -2.54530800 1.29324700

O -1.23951200 0.38390800 0.57025200

H 0.35217300 -3.62275800 1.24645400

H 1.29298100 -2.05326800 1.72443800

Si -0.79842300 2.23939900 0.15539900

C -0.11809500 3.64535300 -0.97583500

H -0.52468800 4.61844900 -0.68493100

H -0.37504200 3.48624800 -2.03017600

H 0.97225700 3.69848500 -0.91229100

C -2.73265300 2.44081400 -0.16061400

C -0.35981100 2.76115400 1.92137100

H -0.74320400 3.76697800 2.12800700

H 0.72714900 2.79111800 2.05760800

H -0.78191200 2.07577700 2.66161400

C -3.11070900 3.91790000 -0.40359600

H -4.20303100 4.01744800 -0.48247500

H -2.68363900 4.31260300 -1.32939000

H -2.78699500 4.56830900 0.41817300

C -3.56260300 1.94926500 1.04606900

H -3.31666200 2.50859300 1.95682400

H -3.40099500 0.88964800 1.24955500

H -4.63609500 2.09826900 0.85454100

C -3.13923700 1.62867900 -1.40833800

H -4.20475500 1.77537500 -1.64042300

H -2.97996200 0.55755700 -1.25284600

H -2.57103400 1.93424300 -2.29667700

N 3.27480500 -0.20597400 -0.74213300

C 3.67641600 1.01319100 -0.02923500

H 4.18194900 1.67218400 -0.74858400

H 2.79609800 1.54185300 0.32438700

C 4.63321100 0.66753300 1.12080400

H 4.08074400 0.08838700 1.87318100

H 4.96231800 1.59375400 1.60587800

C 5.83658400 -0.14433200 0.62118500

H 6.47630700 -0.44230800 1.45942900

H 6.44961300 0.49129000 -0.03358700

C 5.37102500 -1.37641700 -0.16916000

H 4.85791000 -2.07829500 0.50103800

H 6.22615200 -1.90843600 -0.60179700

C 4.39890100 -0.97412600 -1.28710500

H 3.99321200 -1.84412800 -1.80155200

H 4.91588600 -0.34106200 -2.02177800

C 2.03700500 -0.78215600 -0.72308700

O 1.82502700 -1.91876800 -1.16308100

ts6

0 1

O 1.33690800 -1.62072600 -0.58535600

C -1.08975400 2.37792700 -0.22664300

C -1.79912200 3.46627400 -0.73665400

C -1.15702400 2.05324800 1.12978700

C -2.56265200 4.25218700 0.12658200

H -1.77135200 3.67519500 -1.80164700

C -1.93593900 2.83300300 1.98072600

H -0.59475700 1.20077700 1.49517500

C -2.63609600 3.93593700 1.48405300

H -3.11528500 5.09961600 -0.26720800

H -1.99237400 2.58127900 3.03517000

H -3.24294800 4.54036000 2.15099100

N -0.29652200 1.56687800 -1.10861100

C 1.13355900 -0.37628800 -0.73180100

C -0.13639000 0.18846000 -0.86127600

C 0.52461300 2.08792800 -2.00951500

O -1.30641600 -0.49352900 -0.83032100

H 0.68283400 3.15864400 -2.01672300

H 0.78810000 1.48943100 -2.86815900

Si -1.70797600 -1.98080500 -0.10613300

C -0.89926400 -2.14907900 1.58412900

H -1.38988100 -2.92404100 2.18363600

H -0.95346500 -1.21164400 2.14801500

H 0.15113100 -2.41385600 1.44694700

C -3.60178900 -1.78649900 0.07243900

C -1.27200800 -3.40271500 -1.24789800

H -1.54726100 -4.36682900 -0.80571600

H -0.19314200 -3.38303100 -1.41783500

H -1.78435600 -3.31319300 -2.21105800

C -4.21546400 -3.09883100 0.60096100

H -5.30380400 -2.99630500 0.71264700

H -3.81431300 -3.37413400 1.58366500

H -4.03560700 -3.93780400 -0.08091700

C -4.22539200 -1.44903800 -1.29754000

H -4.05373500 -2.24450900 -2.03187100

H -3.81189800 -0.52121800 -1.70521000

H -5.31315900 -1.32109200 -1.20333600

C -3.91422900 -0.64191400 1.05831700

H -5.00020600 -0.50295400 1.15604900

H -3.48529300 0.30706300 0.72121500

H -3.52123500 -0.84961400 2.06046200

N 3.29415400 0.27674600 0.27816100

C 3.28142500 -0.82143300 1.25672000

H 3.52044300 -0.37794200 2.23468800

H 2.29264200 -1.26478000 1.29698100

C 4.33233000 -1.86828400 0.87945400

H 4.01869600 -2.31067600 -0.07229500

H 4.33928400 -2.66654300 1.63064500

C 5.72184200 -1.22441100 0.75933100

H 6.45841100 -1.95632200 0.40917300

H 6.05319100 -0.89631200 1.75529400

C 5.68897900 -0.01041200 -0.18176000

H 5.49771700 -0.34389400 -1.20943700

H 6.65493300 0.50846900 -0.18232800

C 4.58277900 0.97908600 0.21648700

H 4.49647300 1.79962300 -0.49377800

H 4.79370400 1.40593200 1.20684200

C 2.29965300 0.60314000 -0.57836700

O 2.39255900 1.68256600 -1.23760600

cap-PC

0 1

O -1.31798800 0.38786400 -0.23903200

C 3.47652100 -0.64340100 -0.04538700

C 4.42361200 -1.28487300 -0.85508500

C 3.90975000 0.26302400 0.93402700

C 5.78594600 -1.04766500 -0.66690400

H 4.10192700 -1.95270400 -1.64828100

C 5.26988700 0.50687700 1.09930600

H 3.17897100 0.77517000 1.54595700

C 6.21651100 -0.15056300 0.30888800

H 6.50738300 -1.55697700 -1.29907200

H 5.59257600 1.21081700 1.86078100

H 7.27593900 0.04036600 0.44911800

N 2.09459400 -0.92656300 -0.20391400

C -0.25958900 -0.46843800 -0.11618500

C 1.10087700 0.05740800 -0.13221400

C 1.62749000 -2.22996800 -0.61626700

O 1.35630400 1.26349300 -0.07321700

H 2.42292900 -2.96416100 -0.50314700

H 1.25897000 -2.20805700 -1.65369400

Si -1.49674500 1.97319800 -0.82036600

C -3.32388000 1.93208300 -1.30033400

H -3.65981000 2.89444600 -1.69992700

H -3.96254200 1.68508500 -0.44619800

H -3.48759800 1.17082800 -2.07057700

C -1.19412400 3.27578000 0.56876700

C -0.48714000 2.26336800 -2.38071900

H -0.79957600 3.19441200 -2.86765600

H -0.65151300 1.44693400 -3.09294000

H 0.57942600 2.32466400 -2.15909300

C -2.42863300 4.19571100 0.69882900

H -2.25719100 4.94464100 1.48401800

H -3.33440700 3.64178800 0.96748900

H -2.63634600 4.74276900 -0.22841700

C 0.03524600 4.15300600 0.25103200

H -0.09115200 4.70227300 -0.68951100

H 0.94452000 3.55492300 0.17699600

H 0.17871900 4.89942800 1.04509900

C -0.96783200 2.55480200 1.91448300

H -0.82484800 3.29126800 2.71755800

H -0.07997500 1.91926700 1.87468800

H -1.82483900 1.92967200 2.19335700

N -1.63514500 -2.33283200 0.71043700

C -2.68975800 -1.55289600 1.36454600

H -2.94120900 -2.07330800 2.30113200

H -2.30167700 -0.56919400 1.61961300

C -3.93565700 -1.45250900 0.47759000

H -3.67892200 -0.84980200 -0.39935900

H -4.73009900 -0.92578600 1.01949600

C -4.40870400 -2.84967500 0.04619000

H -5.25527100 -2.77183600 -0.64489400

H -4.77026100 -3.39907200 0.92736900

C -3.26151400 -3.64196800 -0.60016400

H -2.96709200 -3.16311000 -1.54291800

H -3.58013300 -4.66405000 -0.83792300

C -2.03682200 -3.69040100 0.32653700

H -1.19161200 -4.18777400 -0.14900200

H -2.28101800 -4.25301500 1.23879500

C -0.48420300 -1.77696700 0.24936400

O 0.56119800 -2.66577300 0.22385000
